# Demonstrating conservation impacts in California Marine Protected Areas using large-scale participatory science data

**DOI:** 10.1371/journal.pone.0352449

**Published:** 2026-06-25

**Authors:** M. V. Eitzel, Nick Ulle, Ryan Meyer, Ben Goldstein

**Affiliations:** 1 Feminist Research Institute, University of California, Davis, California, United States of America; 2 Center for Citizen and Community Science, University of California, Davis, California, United States of America; 3 DataLab, University of California, Davis, California, United States of America; 4 Department of Forestry and Environmental Resources, North Carolina State University, Raleigh, North Carolina, United States of America; National Kaohsiung University of Science and Technology, TAIWAN

## Abstract

Measuring human interactions with protected areas is a key need in conservation social science, both to assess the potential for continuing negative impacts on sensitive resources and to support positive relationships with important places. Our paper investigates human activities in California Marine Protected Areas (MPAs) using an emerging approach of large-scale participatory data collection coupled with statistical analysis. Twelve organizations across the state developed, standardized, and implemented a data collection protocol for volunteers to record human activities, called “MPA Watch.” From 2012−2020, approximately 1,900 surveyors conducted more than 31,700 surveys, observing more than 1.2 million activities at 104 different sites across California’s coast, both inside and outside MPAs. We analyzed these data using generalized linear mixed models (GLMMs) with zero inflation to account for sampling bias associated with volunteer-driven data. We included covariates regarding weather and tides; time of day, week, and year; and beach type and amenities. We found statistically significantly lower consumptive activities inside MPAs with the strictest protections as compared with non-MPAs, and no significant difference between MPAs and non-MPAs for non-consumptive recreational activities. Some consumptive activities significantly declined over time. We also detected expected use patterns (e.g., for all activity categories, weekend counts were significantly higher than weekday counts). In addition, we noted patterns of interest for future study, including much higher incidence of non-consumptive activities than consumptive activities at a statewide level; higher popularity of some activities at certain individual sites; and the beginnings of the effects of the COVID-19 pandemic in 2020. Our analysis demonstrates that consumptive activities are lower inside California MPAs (indicating compliance with MPA rules), and results at particular sites can inform site-specific management strategies. Further, we show the potential of large-scale participatory science, coupled with appropriate statistical modeling, to monitor and inform conservation policy and management for protected areas.

## Introduction

Protected areas are an area-based conservation strategy in which a “clearly defined geographical space, [is] recognised, dedicated and managed, through legal or other effective means, to achieve the long term conservation of nature with associated ecosystem services and cultural values” [1]. Protected areas are an important and widely used mechanism particularly for conserving resources that are susceptible to overuse (the so-called “tragedy of the commons” [2]). Fisheries and ocean resources can fall into patterns reminiscent of the tragedy of the commons, given their open-access nature [3] and though marine protected areas (MPAs) do not necessarily address the question of the global ocean commons [4], they can address some layers of a hierarchical, nested common governance problem [3]. MPAs have thus emerged as a cornerstone strategy in global ocean conservation efforts, with their implementation expanding significantly over the past two decades [5]. These designated zones, where human activities are regulated to protect marine ecosystems, now cover roughly 7% of the world’s oceans, reflecting growing international commitments to marine conservation [6]. However, MPAs, as a governance tool, must balance different uses and multiple ecological and social objectives: regulations may protect one aspect of the system but have unintended consequences on other aspects of the system, affecting people unequally based on their use of the system – potentially raising equity issues.

Protected areas such as MPAs can therefore be controversial due to the potential to include and exclude people unevenly and distribute benefits inequitably [7]. It then becomes useful to ask not only “what are the effects of protection on use of the common resources that can be depleted or consumed (e.g., fisheries or plant resources)?”, but also “what are the effects on other, non-consumptive uses (e.g., local recreation or tourism)?” As the scale of protected areas – marine or otherwise – is growing worldwide, collection of useful monitoring data that can inform both questions of resource protection and equitable access, as well as supporting MPA implementation and management, is a persistent challenge [8,9]. The effectiveness of MPAs varies considerably depending on design, enforcement, and ecological context, and science-based monitoring is essential to understand MPA performance and to optimize their conservation outcomes along multiple dimensions [10]. At the same time, MPAs are more effective when there is shared governance between government and non-government actors [11], and communication between staff associated with protected areas and nearby communities can be key to enhance these relationships [12]. So one way to find out how people are engaging with protected areas in general (and MPAs specifically) in both consumptive and non-consumptive ways, and to support communities impacted by protected areas, could be to engage directly with these relevant communities.

In order to both engage with community members and to address the challenge of collecting long-term monitoring data – sometimes over a large geographic scale – many have pointed to participatory science as a promising approach [13–15]. In natural resources, participatory science programs leverage public interest in common resources, such as wildlife or parks, and recruit volunteers to conduct surveys, sometimes in synergy with their own recreational activities [16]. Because they depend on volunteer effort, some participatory science programs relax the strict protocols of traditional surveys to allow observers some flexibility in choosing when, where, and how to make observations. To date, participatory science projects have yielded observational datasets that greatly exceed those data collected under structured surveys in both volume and coverage [17,18]. Consequently, the use of these data in scientific research is rapidly increasing [18].

While participatory science data have advantages for monitoring—namely their large volume and coverage relative to cost—these data can be difficult to analyze accurately [19]. The sampling process that generates these data is heterogeneous, as participants vary in their effort, interests, and skill [20–22], which could bias downstream inference [19,23]. Such strategies may therefore rely on the analytical phase to enhance the credibility of the results, after data have been collected [24] – which is a key motivation for this paper. There are two broad approaches to making use of participatory science-generated data. First, the participatory science planners can introduce structure on the design side by requiring volunteers to follow particular methodologies to reduce heterogeneity. Participatory science programs must strike a balance between introducing enough structure to help standardize sampling protocols while also maintaining accessibility and fun for participants. One popular design-level choice is to collect metadata describing their sampling, such as the date and length of time of each survey. Datasets in which sampling heterogeneity is allowed and documented are known as “semi-structured” (in contrast to “structured” data with standardized protocols and also to “unstructured” data where heterogeneity is not documented) [18]. The second broad approach, known as model-based inference, uses statistical modeling to account for heterogeneity in the data generating process during analysis, for example by including observed confounding variables or grouping random effects in a multivariate linear mixed model. Semi-structured datasets, which explicitly document sources of heterogeneity, are well suited to analysis using model-based inference. Our study seeks to use both of these approaches in order to mobilize the potential of a large participatory science dataset on the topic of MPAs.

Specifically, the goal of this study is to use model-based inference with semi-structured participatory science data to answer questions about human activities in MPAs across the state of California during 2012–2020. Surveyors in the MPA Watch program [25] collect data following a set protocol, in predetermined locations, but sampling effort is highly variable across space and time, and some activities may not be observed in a given survey. We used a zero-inflated Generalized Linear Mixed Model (GLMM), including a variety of predictors controlling for seasonality, spatial relationships, and features associated with survey locations, in order to address two key questions for each of seven broad human activity categories: Q1) Are there differences in activity in MPAs with no ‘take’ allowed, MPAs with more varied rules about what visitors can remove, and non-MPAs with no restrictions? Q2) Are there differences over time in activity counts, and do differences depend on level of protection? We also ask: Q3) What can MPA Watch data tell us about potential violations of the Marine Life Protection Act? Q4) What are the seasonal, diurnal, and weekly patterns in activity? Q5) What features of individual locations affect human activities?

## Materials and methods

### Study system: California Marine Protected Areas (MPAs) and MPA watch

In California, a network of 124 marine protected areas have been established in coastal waters under the California’s Marine Life Protection Act (MLPA) [26]. Multiple state agencies and other partner organizations, including federal agencies, private funders, and other organizations, have collaborated to implement and manage the network [27,28]. The MLPA calls for adaptive management of MPAs, for the benefit of ocean health both inside and outside of MPAs. Monitoring is a crucial component of measuring progress toward MLPA objectives, which include opportunities for recreation, education, and scientific study, in addition to improved biodiversity and ecological health [26]. The state has invested millions of dollars in monitoring projects cutting across a wide range of disciplines and focal areas [29]. A notable feature of the state’s approach to MPA monitoring has been the emphasis – from the beginning – on participatory science approaches that can integrate with or supplement traditional professionalized approaches [30–33].

The data underlying our model come from surveys run by the MPA Watch program. MPA Watch is a participatory science network, with chapters run by non-profits, state agencies, and Tribal groups throughout the California Coast. While there have been a handful of state-funded research projects focused on human dimensions of California’s MPAs [34,35], MPA Watch is the only ongoing monitoring program on this topic, and the only one that examines both consumptive and non-consumptive human uses at a statewide scale. Initially, MPA Watch was supported by private funders collaborating with the state on MPA implementation, and more recently the California Ocean Protection Council has provided funding for both capacity building and analyses (including this study). Both private and state funders of the project are active participants in collaborative MPA implementation.

### MPA Watch activity count survey protocol

**Fig 1:**
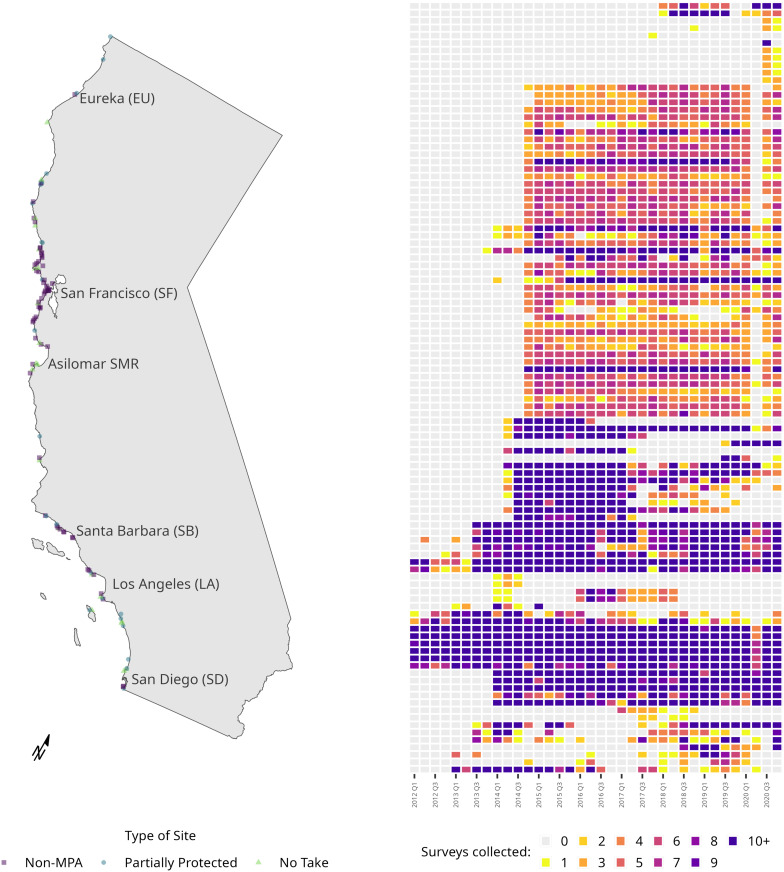
Sites and sampling effort over time. The full California Marine Protected Area network includes over 200 sites across the entire coast; shown at left are the 102 sites with MPA Watch surveys analyzed in this paper. On the right is the number of surveys per quarter for each site (ordered from most northern at the top through most southern at the bottom). Data collection was fairly widespread starting in 2014, with some sites in northern California starting only recently. Note the gap in data collection in Q2 2020, during the shelter-in-place orders of the COVID-19 pandemic. State boundary available from California’s open data repository: https://data.ca.gov/dataset/california-state-boundary.

In the MPA Watch program, each of twelve participating organizations is responsible for training adult (over 18) volunteers, who then follow a statewide protocol designed to consistently gather counts of consumptive and non-consumptive human activities at coastal sites throughout California. The program began in Monterey county in 2011 following the establishment of the new network of MPAs in state waters, and by 2016 had spread to organizations and sites in every coastal county of the state (Fig 1). MPA Watch is intended to inform management and implementation of MPAs, while also educating local residents about marine conservation and engaging them in stewardship – in alignment with the State’s partnership-based approach to MPA implementation [28]. Since agreeing upon a statewide protocol, MPA Watch has gathered more than 49,000 surveys across 203 transects within 103 sites (as of June 2024), amounting to a unique and potentially rich source of data about human activities. MPA Watch data could also be used for more than just enforcement of the MLPA and education of coastal users, so the current study supports these other potential uses by investigating what is possible when analyzing the MPA Watch archive with appropriate model-based inference. Other uses include a variety of human dimensions research topics on coastal human activities as well as local organizations’ and communities’ needs to understand the pressures on and opportunities associated with their coastal places.

In the protocol, MPA Watch surveyors walk a pre-set transect along the coastline, tallying counts of human activities on a datasheet with predefined categories. Counts are largely of individual humans, although there are also categories for domestic animals and boats of various types. Observers record a variety of metadata, including weather conditions, tides, date and time, and site features. Although the sites and transects are pre-defined, surveyors engage in the activity in a timeframe and level of frequency that works best for them personally. Some programs also count human activities from a boat for particular transects, and refrain from counting certain shore-based activities that are difficult to discern from that vantage point. (Some of these sites have corresponding land-based counts as well.) We only included both shore-based and boat-based counts for offshore recreation and recreational boating (which are themselves counts of boats, not people). For a detailed accounting of the data collection protocol, see [36] for the MPA Watch training manual.

In our analysis, the specific categories of activities from the surveys (see example data sheet at [36]) have been grouped into larger categories (Table 1). By aggregating activity categories, we avoid the difficulty with fine classifications of different activities that may vary in different parts of California, and by choosing an appropriate scale of categorization we also avoid some of the statistical difficulties associated with activity categories that are rare. For example, combining multiple board sports (surfing, boogie-boarding, and ‘general board sports’) into the larger ‘offshore recreation’ category avoids any inconsistencies between different programs categorizing these activities either more generally or more granularly. The effect of this aggregation is to increase the sample size for a broad category (e.g., ‘offshore recreation’) and for less-frequented coastal sites this can make it easier to statistically analyze the effect of predictors on activity counts; when there are few occurrences of an activity it can be difficult to estimate the effect of a given predictor.

**Table 1:** Activity categories from the MPA Watch surveys. “Consumptive” refers to the removal of materials from the Marine Protected Area or other coastal place.

Activity Category	Description
Onshore recreation	Shore-based recreation (except tidepooling).
Tidepooling	Shore-based non-consumptive tidepool viewing.
Domestic animals	On- and off-leash domestic animals (mostly dogs); people accompanying the animals are counted in “onshore recreation”.
Offshore recreation	Swimming, non-consumptive SCUBA or snorkeling, non-consumptive diving, and board sports (surfing, boogie-boarding, stand-up paddleboarding, kitesurfing, windsurfing).
Recreational boating^1^	Paddle-operated boats, kayaks, power boats, sail boats, jet skis, dive boats, whale-watching boats, and sport fishing boats. Counts of boats, not people.
Onshore fishing	Shore-based hook & line, trap, net, and spear fishing.
Hand-collection	Collection of biota, e.g., removing invertebrates from a tidepool.
Offshore fishing^1^	Boat-based hook & line, trap, net, spear, and dive fishing; recreational, passenger, and commercial boats; active fishing only (boats not actively fishing are allowed to transit through MPAs). Counts of boats, not people.
Total activities^1,2^	Aggregates all the above categories, plus additional more infrequent activities reported on the survey sheet; see [36] for a full list.

^1^Uses data from both boat-based and land-based surveys.

^2^Not statistically modeled because it is an aggregate of other categories.

One topic of great interest to both the State of California and to MPA managers is compliance with the Marine Life Protection Act (MPLA). In order to inform this question, we pay special attention to the prevalence of consumptive activities (onshore fishing, offshore fishing, and hand collection of biota) specifically in no-take MPAs, where any consumptive activity is a potential violation of the MLPA. These are only potential violations because the protocol does not include mechanisms for determining whether the take was actually legally permitted, e.g., if visitors were engaging in Tribal cultural practices or scientific studies. The full protocol and dataset also include the surveyors’ assessment of whether a violation is occurring, but this was less consistently applied across different observers. This is partly due to not all surveyors having filled out this category on the data sheet, including differences in whether they reported the potential violation to authorities. In addition, surveyors may also have differed more widely in their assessment of whether an activity was a potential violation, because violations are determined by often-complex rules regarding what is allowed in that location (for example restrictions on specific species or fishing equipment, which may be difficult to see). Assessing a potential violation directly therefore requires a different level of judgment from surveyors that can vary more widely than for other categories. Therefore we focus on the definition of a potential violation based on activity category and MPA type because we were able to consistently apply it across the dataset.

#### Predictor variables.

Transects were set up both within MPA sites throughout the length of California’s coast, and in non-MPA sites, to allow comparisons between locations that have been designated as protected versus those that have not. These non-MPA sites are not necessarily chosen to deliberately contrast with specific MPAs, so our analysis does not rely on paired tests and instead treats them as separate groups. This limits the strength of our comparisons, and instead we categorize each transect as within either 1) a non-MPA site, 2) a no-take MPA (State Marine Reserves and no-take State Marine Conservation Areas, because they have the strictest and most consistent rules regarding what visitors are allowed to do), and 3) any other type of MPA which is partially protected and allows some take (considered together as one group). This variable addresses research questions Q1 and Q3. We include the year of the survey as a categorical factor, allowing for individual estimates for each year and addressing Q2. For recreational boating and offshore fishing, we also include boat-based surveys, and include a categorical factor in the statistical models representing the difference in the land-based versus boat-based protocols.

We discussed other potential predictor variables with MPA Watch volunteer managers, and included variables they judged to be important, as well as those that we felt were important to control for based on the experimental design. These variables related to the survey process itself, the timing of the survey, the location of the survey, the characteristics of the transects, and weather and tide conditions at the time of the survey. We standardized all continuous predictors (subtracted the mean and divided by the standard deviation) to improve the optimizer’s ability to find a maximum likelihood estimate.

Table 2 lists all the potential predictors in our models. Not all predictors were included for every activity category; see S1 Appendix for which variables were included for which activity categories, and for additional detail on the data handling and/or construction of the predictor variables.

**Table 2:** Predictors included in models of activity counts. “Scale” refers to whether the variable pertains to one individual survey, all records associated with a given transect, or all records associated with a given site.

Name	Units	Scale	Description
***Key Questions (Q1, Q2 & Q3)***			
MPA status (Q1 & Q3)	Categorical	Site	Non-MPA, no-take MPA, partially-protected MPA
Pattern over time (Q2)	Categorical	Survey	Categorical factor for each year (2012–2020)
Time × MPA status	Categorical	Survey	Interaction between MPA type and categorical year, allowing for different time patterns for each MPA category.
***Site/Transect Features (Q5)***			
Access/amenities index	Unitless	Transect	Index ranging from 2 to 14 (see S1 Appendix)
Beach type	Categorical	Transect	“Sandy,” “Rocky,” or “Sandy and Rocky”
Has tidepooling	Categorical	Transect	Is the transect a site known for tidepooling? (yes/no)
Population density	Persons/km^2^	Site	Mean over 2012–2019 for census tracts within 15 miles
State park proximity	Categorical	Transect	Is the transect immediately adjacent to a State Park? (TRUE/FALSE)
***Spatial Variables (Q5)***			
Latitude	Degrees	Transect	Includes polynomial terms from degree 2 up to 8 depending on the activity category
Site	Categorical	Site	Random effect to reflect site-level variation not captured by other covariates
Transect	Categorical	Transect	Random effect to reflect transect-level variation not captured by other covariates
***Temporal Variables (Q4)***			
Day of week	Categorical	Survey	Sat, Sun, Mon, Tues, Wed, Thu, Fri
Time of day	Hrs & Min	Survey	Time of the survey start; linear and quadratic terms
Seasonality	Day of year	Survey	Represented by two variables (“winterness” and “springness;” see S1 Appendix)
***Survey Method Variables***			
Survey duration	Minutes	Survey	Time spent surveying, typically one hour
Transect length	Miles	Transect	Length of transect as originally planned
Survey type	Categorical	Transect	Boat-based or Land-based (only for some categories, see Table 1)
Program	Categorical	Transect	Random effect to reflect variation from one MPA Watch program to another
***Weather/Tide Conditions***			
Tide level	Unitless	Survey	Scaled tide height (see S1 Appendix)
Clouds	Categorical	Survey	Clear, Partly Cloudy (1–50% cloud cover), or Cloudy (>50% cloud cover)
Precipitation	Categorical	Survey	Yes/no
Visibility	Categorical	Survey	Perfect (to horizon), limited (beyond shore, but not to horizon), or Shore Only

To prepare for modeling, we organized the data into a matrix with each row representing a survey associated with an observed count for each category in Table 1 and values for all predictors listed below (data downloaded on March 14, 2022). We omitted records in which a surveyor did not provide information on all predictors, reducing the number of records from 29,353 to 27,088 for only land-based surveys and from 31,725 to 29,413 when boat-based surveys are included. These omitted records (2,265 for land-based; 2,312 overall; around 7.7% of surveys) were largely in the first year (2012) and for particular sites (e.g., Abalone Cove), and the missing variables tended to be the weather variables – likely due to early efforts to align protocols between sites and programs. This omission is likely to reduce the precision of early-year data and/or estimates for particular sites by reducing the sample size, but other data in those years and for those sites can be used to compensate, and random effects for sites and separate factors for the year will also help control for these differences. We included all transects in our analysis, but in figures we did not display the parameter estimate for transects associated with fewer than 10 surveys.

Note that this study makes use of pre-existing data and does not collect any new data on human subjects. In addition, the data are available publicly online. Any information about volunteer surveyors that collected the data was removed from the database before we analyzed it; and the humans engaging in coastal activities that the surveyors are observing and counting are in public places. Thus, we did not have access to information that could identify individual participants during or after data collection. Given this, we have not sought Institutional Review Board Human Subjects review.

### Generalized linear mixed model implementation

We used Generalized Linear Mixed Models (GLMMs) to model counts of each human activity type. We modeled each activity category separately, following a negative binomial distribution, which can represent counts with dispersion (allowing for outliers). For detailed technical descriptions of how we made specific choices in model implementation, see S1 Appendix.

We estimated model parameters using glmmTMB (version 1.1.10) [37] in R (version 4.4.2) [38] which can accommodate the negative binomial distribution, as well as zero-inflation (having more zero counts than would normally be found in the negative binomial distribution). To assess model fit, choose between possible specific versions of the statistical distribution, and decide whether to include manager-selected variables in certain ways, we used DHARMa (version 0.4.7) [39] to visualize and statistically test model residuals: we checked to ensure that remaining variation in the data after taking into account the model structure (residuals) did not show patterns which would imply the need for other model structures or different statistical distributions.

For our key questions regarding activities inside and outside MPAs based on the MPA’s type of restrictions (Q1 & Q3), and the potential patterns across time (Q2), we also included an interaction between site type and year, in order to see how usage patterns may have changed differently over time for each category of site (no-take MPAs, partially-protected MPAs, and non-MPAs).

#### Dispersion.

By using a negative binomial distribution, we were able to accommodate dispersion in observed counts, when there is more or less variation in counts than would be expected for a more commonly-used distribution (e.g., Poisson). In addition, predictor variables can be included in the dispersion parts of the model – reflecting that some types of sites have greater variation than others. Most activity categories only warranted a model structure with an overall over- or under-dispersion, but for hand collection of biota, assessing the residuals indicated that the beach type and whether it was a known tidepooling site also impacted the dispersion. This reflects that, for example, there could be more variation for a rocky transect than for a sandy one.

#### Zero-inflation.

We also included zero inflation, where the data include additional zero observations beyond what might be expected from the assumed distribution. For example, these “extra” zeroes in MPA Watch surveys could be due to mismatch in timing between surveyors and coastal users (surveyors simply are not present when individuals are engaging in activities), and/or because surveyors check for all activities every time they survey and they could be doing so at a time or place which is appropriate for one activity but not another. This is in addition to the underlying distribution of counts which could include some zeroes as part of the underlying process, for example zeroes occurring by chance or for a site with lower counts overall. For most activity categories, our models included a constant probability that a survey recorded “extra” zeroes. For tidepooling, our assessment of the model residuals indicated that the model needed to account for the fact that sites not known for tidepooling were more likely to have zero tidepoolers than a site that was known for tidepooling. And for onshore recreation, which is a much more common activity than the others that was essentially always present, we did not need to include zero inflation.

#### Random effects.

For all activity categories, we included random effects are intended to account for additional unknown factors that could differ from transect to transect or site to site that were not accommodated by the other covariates in the model. We also included random effects for MPA Watch program for some of the activity categories (when the residuals warranted it), accounting for the possibility that programs may train surveyors in slightly different ways.

#### Statistical inference.

We used a variety of different statistical tests appropriate for each of the covariates (for example, continuous variables, versus categories with two options, versus categories with more than two options) to assess significance within an activity category. Because of the large number of predictors and comparisons there were 624 tests total for all activity categories. Doing a large number of significance tests increases the chance of incorrectly finding a comparison to be significant by chance, so we applied a multiple testing correction to all p-values WITHIN each activity category.

#### Aggregated total activities.

For the “Total Activities” category, which includes all the other categories listed in Table 1 as well as other smaller categories that were too rare to be separately modeled (see [36] for the full datasheet and S1 Appendix for descriptions of all activities), we display visual summaries of modeling results, but do not calculate statistical results. This category is included for illustrative purposes and to give context to the magnitude of the other activity category model predictions, but in order to preserve the validity of p-values, we do not draw statistical conclusions regarding it because it includes data that have already been used for inference for the other categories.

## Results

There was less consumptive activity in MPAs than outside (research question Q1) – especially in no-take MPAs (research question Q3), and we found declines in consumptive activities over some time periods (for example, less onshore fishing in 2015–2019 than in 2013–2014 and 2020; research question Q2). We identified statistically significant logical seasonal patterns, higher midday counts, and higher weekend counts (research question Q4). Rain tended to lower counts, recreation activities were higher for sandy beaches, and low tide favored onshore activities. Greater population density and access/amenities did increase some activities, and onshore fishing was higher immediately next to a state park, while offshore fishing was lower (research question Q5). See below for more detailed, activity category specific results on each research question, and refer to S1 Appendix for specific numerical values.

In terms of general patterns that we observed but did not test statistically, consumptive activity was overall much lower than non consumptive activity throughout the coast, regardless of MPA status. We also observed particular individual site patterns, relative popularity for particular kinds of activity, and regional patterns across space. We see the very beginning of the impacts of the COVID-19 pandemic potentially indicating higher human activity corresponding to the shelter-in-place orders. We first review the statistical results, and then the qualitative patterns.

### Statistical results

**Fig 2:**
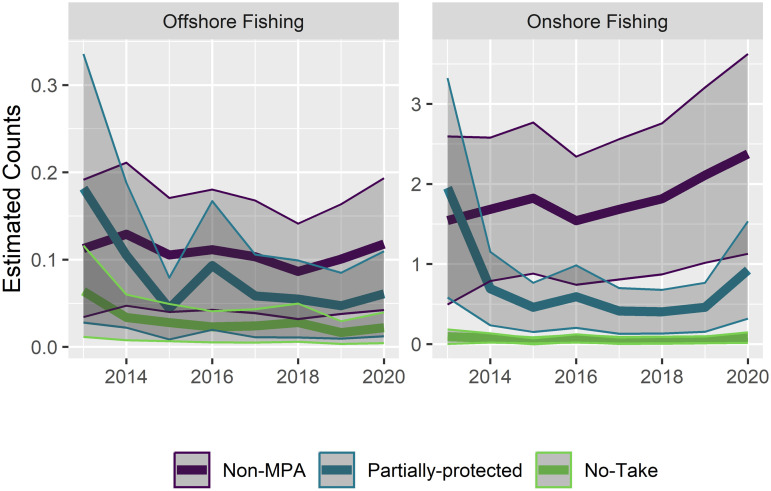
Time patterns for each category of site (No-Take MPAs, “Other” partially-protected MPAs with some take allowed, and non-MPA sites). Grey bands indicate 95% predictive error intervals.

Fig 2 and Tables 3-5 summarize the highlights of the results for each activity. See S1 Appendix for similar tables with all statistical results summarized, and complete tables with all p-values and parameter estimates.

#### Key questions Q1, Q2 and Q3: MPA effects and year-to-year differences.

For many activities, there were no significant differences between MPA types or patterns over time. However, we found that for onshore fishing, no-take MPAs had significantly lower counts than either of the other two categories, and for most years (2015 and 2017–2019), partially-protected MPAs had significantly lower counts than non-MPAs (Fig 2). For offshore fishing, for some of the middle years of the time period (2016 and 2019), there was significantly less fishing inside no-take MPAs than in non-MPAs. Recall that the omission of early records that had incomplete weather data means that precision of the 2012 estimate may be slightly less robust (hence the larger predictive uncertainty range shown in Fig 2). That said, the differences we highlight are still present even taking into account the greater uncertainty range. Time patterns are suggestive and largely qualitatively assessed, though some specific comparisons are statistically significant (see S1 Appendix for all p-values and parameter estimates).

#### Features of transects/sites (Q5).

Sandy beaches, and combinations of sandy and rocky beaches, had greater activity for onshore and offshore recreation as well as domestic animals (Table 3). Recreation, onshore fishing, and domestic animals had higher counts for transects with more amenities and easier access. Tidepool counts were, as expected, higher for sites that were known for their tidepooling (regardless of their MPA status). Higher population density (average people/km over 2012–2019 within 15 miles) was associated with higher counts in recreational boating and domestic animals, but was not significant for other activities (which could indicate that a different metric of population density would be more appropriate for those activities). Finally, being immediately adjacent to a State Park was associated with higher onshore fishing counts and lower offshore fishing counts.

**Table 3:** Results for transect-related features (Q5). Access and amenities were represented as an index; beach type was either sandy, rocky, or sandy and rocky; adjacency to a state park means immediately adjacent; some sites are known for tidepooling; and population density was calculated as persons per square kilometer over 2012-2019 for census tracts within 15 miles. Note that ‘beach type’ was not included in offshore fishing or recreational boating, and whether the site was a known tidepooling site was only considered for onshore recreation, tidepooling, and hand collection of biota. The access and amenities index, population density, and adjacency to a State Park were all considered for all activity categories.

Activity category	Highlights
Onshore Recreation	More access and amenities has a positive effect; sandy beaches have more activity
Offshore Recreation	More access and amenities has a positive effect; sandy beaches have more activity
Onshore Fishing	More access and amenities has a positive effect; being adjacent to a state park has a positive effect
Offshore Fishing	Being adjacent to a state park has a negative effect
Tidepools	Being a known tidepooling site positively affects activity counts; non-tidepooling sites have many more zeroes than tidepooling sites
Recreational Boating	Higher population density has a positive effect
Domestic Animals	More access and amenities has a positive effect; sandy beaches have more activity; higher population density has a positive effect
Hand Collection	For tidepooling sites, there was less variation than for non-tidepooling sites

#### Spatial variables (Q5).

In terms of linear trends in latitude, offshore recreation and hand collection showed higher counts for more southern sites; and recreational boating had a complex relationship with latitude, but with generally higher counts through middle latitudes. In addition, random effect standard deviations for both sites as well as transects within sites were reasonably large, with the most variation occurring in tidepooling, indicating important additional spatial variation at a variety of scales. See S1 Appendix for specific comparisons of activity patterns across the state. Recall that some of the omitted data due to incomplete weather observations was particularly concentrated in the early years of some specific sites, so those random effect estimates are likely affected (e.g., Abalone Cove may have a less-precise random effect estimate because its sample size is smaller).

#### Temporal variables (Q4).

Weekends had greater counts than weekdays for all activities, and for several activities (offshore recreation, recreational boating, and onshore fishing) Fridays were significantly higher than other weekdays (Table 4). For other activity categories, some weekdays were also greater than others. Fishing categories peaked in the morning, and other activities in the afternoon, with domestic animals peaking as late as almost 5 pm. Onshore fishing was highest in late June, and onshore recreation, hand-collection, tidepooling, and animals were highest in early July, recreational boating and offshore recreation in late July, and offshore fishing in mid-September.

**Table 4:** Results for temporal variables (Q4). For all activity categories, the linear and quadratic terms for time of day were statistically significant, so we share an estimate of peak time of day. For our two time-of-year variables, “winterness” and “springness,” one or both were significant for all activity categories so we share an estimate of peak time of year. See S1 Appendix for the specific times of day and days of year.

Activity category	Highlights
Onshore Recreation	Weekends greater than weekdays; peak activity early afternoon and early July
Offshore Recreation	Weekends greater than weekdays & Fridays greater than other weekdays; peak activity early afternoon and late June
Onshore Fishing	Weekends greater than weekdays & Fridays greater than other weekdays; peak activity mid-morning and late June
Offshore Fishing	Weekends greater than weekdays; peak activity mid-morning and mid-September
Tidepools	Weekends greater than weekdays; peak activity early afternoon and early July
Recreational Boating	Weekends greater than weekdays & Fridays greater than other weekdays; peak activity early afternoon and late July
Domestic Animals	Weekends greater than weekdays; peak activity late afternoon/early evening and early July
Hand Collection	Saturday greater than weekdays; peak activity early afternoon and early July

#### Survey method variables.

Including variables representing survey design features (e.g., how long a transect was, whether it was conducted from a boat or from shore) generally improved model fit, and some were statistically significant in predicting counts (see S1 Appendix for parameter estimates). Boat-based surveys observed significantly more offshore fishing than shore-based surveys (which was expected but not necessarily obvious, as shore-based surveyors use binoculars to identify offshore activity). Transect length was not significant across most categories; however, for domestic animals the residuals warranted a squared term, and it was statistically significant and negative – implying that there were larger numbers of animals on transects with intermediate lengths. Survey duration was always significant, with longer duration surveys observing higher counts, and for domestic animals, the same quadratic relationship largely held (medium-duration surveys had higher counts, though longer surveys still had higher counts than shorter surveys).

Random effects standard deviations accounting for variation from program to program were generally smaller than the transect and site random effects, and were highest for recreation, tidepooling, and hand-collection, and small for onshore fishing. This effect could point managers towards places where protocols may need to be realigned between programs. The other three categories’ residuals were not improved by including this effect.

#### Tide level and weather.

Rain negatively impacted all activities but offshore fishing and hand collection, while most shore-based activities were impacted by tide levels (Table 5). Higher counts of onshore recreation, tidepooling, domestic animals, and hand collection were all associated with lower tide levels; while onshore fishing, offshore recreation, and recreational boating showed no relationship.

**Table 5:** Results for tide level and weather. Variables include precipitation (yes/no), visibility level, cloud cover, and tide level. Note that all of the weather variables were included for all activity categories, but tide level was not included for offshore fishing.

Activity category	Highlights
Onshore Recreation	Lower tide increased activities; rain decreased activities; clear weather with limited visibility increased activities
Offshore Recreation	Rain decreased activities; clear weather with limited visibility increased activities
Onshore Fishing	Rain decreased activities
Offshore Fishing	Cloudy weather increased activities, and better visibility increased activities
Tidepools	Lower tide increased activities; rain decreased activities; limited visibility increased activities
Recreational Boating	Rain decreased activities; clear weather and good visibility increased activities
Domestic Animals	Lower tide increased activities; rain decreased activities; clear or partly cloudy weather and good visibility increased activities
Hand Collection	Lower tide increased activities; cloudy weather increased activities

Non-consumptive activities tended to have higher counts with lower cloud cover (clear, or < 50% cover), while consumptive activities tended to have higher counts with higher cloud cover (greater than 50% cover). Specifically, lower cloud cover was associated with more onshore and offshore recreation as well as tidepooling, domestic animals, and recreational boating. Onshore fishing was unaffected by cloud cover, while offshore fishing and hand collection were greater for greater cloud cover.

Limited visibility (either shore only, or some water visible, but not all the way to the horizon) was associated with increased onshore and offshore recreation, as well as tidepooling, while perfect visibility (all the way to the horizon) was associated with increased recreational boating, domestic animals, and offshore fishing. Onshore fishing and hand collection were unaffected by visibility conditions. This finding may reflect that when visibility is poor enough not to see any activity at all, surveyors do not do surveys. When visibility is not perfect, the weather may be more moderate, encouraging coastal visitation, or there could be less glare, improving the ability of surveyors to count activities. These explanations derive from our personal experience and would need to be systematically tested, however.

Generally speaking, the relationship between activity and weather conditions is complex, in which cloud cover and visibility may have counter-intuitive impacts on certain activities, but precipitation generally does result in fewer activity counts.

#### Dispersion and zero-inflation.

Including dispersion improved the qualitative appearance of the residuals for all the categories, and statistical tests of the residuals indicated no additional dispersion. Many of the activity categories had dispersion parameters substantially larger than one (implying that there are many more larger counts than would be typically for the underlying distribution), with tidepooling and both onshore and offshore recreation in particular being strongly overdispersed (see S1 Appendix for parameter estimates). The domestic animals category was challenging to find a good distributional fit for, and therefore the dispersion is not large but also the least well-accounted for. Zero-inflation parameters were quite high for offshore fishing and domestic animals, meaning that there were many more surveys that observed none of these activities than would be expected for the underlying distribution (likely indicating that there were additional reasons not to observe those activities at the times/locations of those surveys). But even for other categories, zero inflation was important in improving model fit (the residuals indicated no additional zero inflation was present once it was accounted for in the model).

### Qualitative patterns and observations

**Fig 3:**
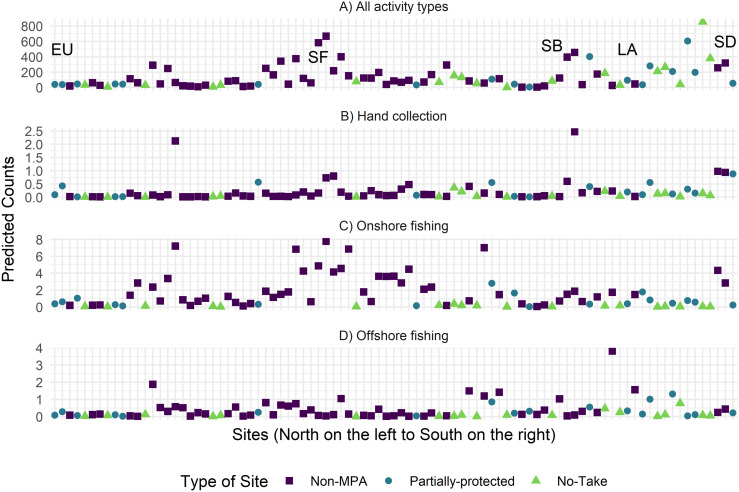
Model-predicted counts across all sites in California for all consumptive activities, under standardized favorable conditions (sunny weather at midday, mid-summer, on a weekend day, with average tide level) – note that these are NOT raw counts from the data. Each point is the average prediction for all transects within that site, for one-hour, one-mile-long transects; sites are arranged from north at the left to south at the right (see Fig 1 for the actual geographical arrangement of sites, but certain locations in California are shown for reference: EU = Eureka, SF = San Francisco Bay Area, SB = Santa Barbara, LA = Los Angeles, SD = San Diego). A) Predictions for all activities on the survey sheet, shown for context with the other activities. Below the total activities are predictions for B) hand collection of biota (e.g., removing invertebrates from a tidepool), C) onshore fishing (using a variety of different gear), and D) offshore fishing (using a variety of different types of gear; note that this is a prediction of number of boats, not individual people).

**Fig 4:**
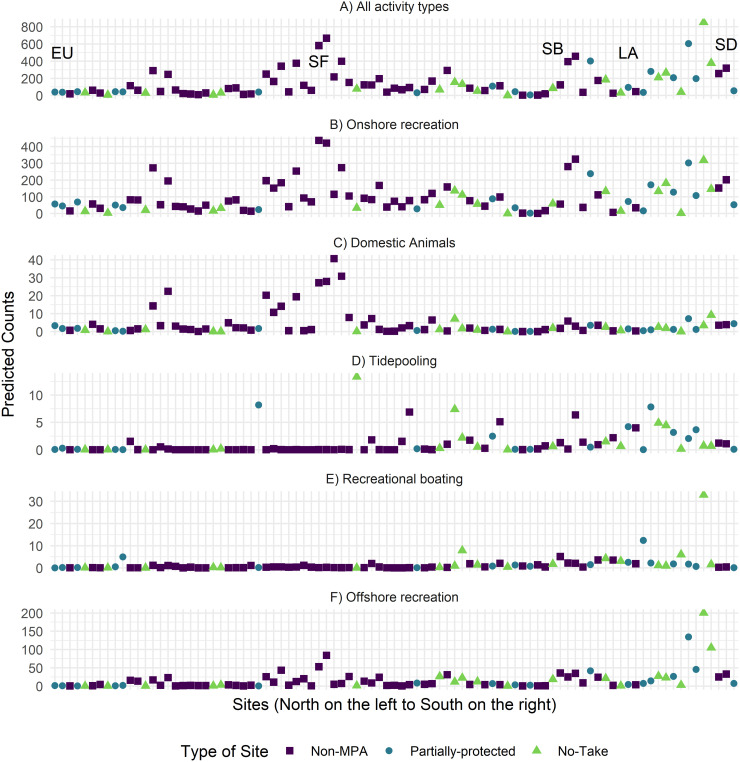
Model-predicted counts across all sites in California for all non-consumptive activities, under standardized favorable conditions (sunny weather at midday, mid-summer, on a weekend day, with average tide level) – note that these are NOT raw counts from the data. Each point is the average prediction for all transects within that site, for one-hour, one-mile-long transects; sites are arranged from north at the left to south at the right (see Fig 1 for the actual geographical arrangement of sites, but certain locations in California are shown for reference: EU = Eureka, SF = San Francisco Bay Area, SB = Santa Barbara, LA = Los Angeles, SD = San Diego). A) Predictions for all activities on the survey sheet, shown for context with the other activities. Below the total activities are predictions for B) onshore recreation, C) domestic animals (a count of the animals themselves, usually dogs; the humans associated with them are counted in onshore recreation), D) tidepooling, C) recreational boating (for example, kayaking; note that this is a prediction of number of boats, not individual people), and D) offshore recreation (for example, SCUBA diving or surfing).

As we observed in the raw data [25], there were more nonconsumptive activities than consumptive activities statewide, regardless of the site’s protection level. Note in Figs 3 and 4 that the order of magnitude of model predictions for nonconsumptive activities are much larger than the consumptive activities.

Some time patterns emerged from our analysis that could be more systematically studied. For example, onshore and offshore recreation increased from 2013 to the middle of the time period, and then increased again in 2020 (See S1 Appendix; possibly due to the COVID-19 pandemic and shelter-in-place orders). A study framed specifically around the pandemic could make use of more recent data and methods developed for modeling the different phases of the pandemic; see the Discussion section for further thoughts. Another possible pattern is the drop in counts in the fishing categories from 2013 to the middle of the time period for the partially-protected MPAs. It is difficult to assess whether this is due to implementation of MPA restrictions phasing in over that time, because no systematic activity data were collected before implementation. However, an analysis that included a variable for “how long has this area been protected” (which would be the longest for many of the no-take MPAs, which have been protected in various ways prior to the MLPA) might enable us to test some of these patterns that may be related to implementation.

Regionally, onshore recreation is high in the San Francisco Bay Area (“Bay Area”) and in the Los Angeles and San Diego areas – but domestic animals are particularly prevalent in the Bay Area and tidepooling is higher in the Los Angeles and San Diego areas (though Duxbury Reef State Marine Conservation Area in the north, and Montara State Marine Reserve and Asilomar State Marine Reserve in the central coast are high, in addition to Crystal Cove State Marine Conservation Area in the South). Onshore fishing is more common in the center of the state and in the north, relative to the south, while the reverse is true of offshore fishing.

There are particular hotspots of total activity in San Francisco (Ocean Beach), near Santa Barbara (Carpinteria), and in San Diego (Matlahuayl State Marine Reserve and Swami’s State Marine Conservation Area). At Matlahuayl, this is driven largely by recreational boating and offshore recreation, and Carpinteria is popular for hand collection of biota (as is Tijuana River in San Diego, and Brazil beach just north of the Bay Area).

## Discussion

Complex participatory science data can be challenging to model but such efforts can be fruitful when appropriate statistical approaches are selected. Our work with the MPA Watch program and its data offers several key insights, stemming from both the collaborative process and the results of the analysis. We highlight them here and then explore further in later sections.

First, the collaborative modeling process does add its own complexity and lengthens the timeline, but also leads to modeling that is better founded in the contextual realities of the system, and results that are more relevant to stakeholders’ questions. Iterative feedback loops between the participatory science network, analysts, and marine protected area managers helped identify appropriate research questions, improved analytical approaches, and increased the utility of results in terms of management actions and potential program improvements.

Second, our analysis allows MPA Watch data to speak to important policy questions. For example, we were able to show that compliance with MPA regulations regarding consumptive activity is high (research question Q3), as well as investigating temporal and spatial patterns of activities (Q4 and Q5): Levels of consumptive activity in no-take MPAs are very low across the state, especially relative to overall activity levels, and the vast majority of human activities along the coast – inside and outside of MPAs – are non-consumptive. These high-level takeaways, along with more granular results for specific sites or activities, can be of use to the broad array of actors engaged in marine conservation in coastal California.

### Caveats and limitations of data and analysis

We first acknowledge the caveats and limitations of our study. One key limitation is that the non-MPA sites were not designed as controls for the MPA sites, limiting the strength of the comparison. Though we include a variety of site features to control for some of the known differences, there may be other factors that could bias the results that could have been avoided with an explicitly paired design. See below for possible ways to use causal inference methods to correct for this limitation. We also modeled each activity category separately, which does not allow us to give statistical significance to any comparison between them and restricts us to qualitative comparisons. There are also some limitations in interpretation due to data omission from incomplete weather observations, though our model structure does reduce the impacts on estimates for those early years and particular sites and uses other data to compensate.

We also note that our study is a social science analysis of human activities, rather than an analysis of impacts on marine resources themselves. A future study could synthesize MPA Watch counts of humans with, e.g., fisheries data or other data sources to establish whether even a small number of people fishing in an MPA causes a large impact on the resources to be protected, and this could give better context to how important even a small number of potential violations might be. Future work could also synthesize MPA Watch data with other data on violations. In addition, this dataset was intended to be used both for understanding activities at local sites and also statewide – but the study is fundamentally observational and while we included as many predictors as were available to control for the wide diversity of the California coast, generalizations across the state may still be limited by site-to-site differences we were unable to account for. The broad conclusions of our work should be general enough to be useful for statewide policymakers, but should be checked with other data sources and validated with communities and managers at local sites to enhance its utility.

### Lessons learned analyzing large participatory science datasets

Our earlier efforts to work with the MPA Watch data centered on an occupancy model [25], which revealed some patterns about human coastal use, but we were unable to accommodate survey-level information (because surveys were aggregated in order to estimate detection probability along with presence/absence of a given activity). This aggregation only allowed us to model very broad patterns (e.g., whether at least one person was doing that activity at some point in a given year in a given location). We embraced the generalized linear mixed model framework in this analysis because it allowed us to include the abundance of activities, as well as variables like time of day, weather and tide conditions, and much more that was elided when the surveys were aggregated in the occupancy model. We found that some of the results were good validation of the method (e.g., weather and time patterns were as expected), thus building confidence in the results that address the key questions about activities inside and outside different types of MPAs. With the GLMM, we were also better able to show differences between sites and to make clearer statements about the potential violations in no-take MPAs. The reason, however, that we had initially used an occupancy model was that some of the predictor variables (for example, visibility) could impact both detection as well as abundance, in which the surveyors may have more difficulty seeing an activity with poor visibility, but also people may choose not to do that activity when visibility is poor. Models that account for the separate impact of predictors on detection and abundance, like N-mixture models, are a potential option for this dataset; however, even more data would be required to parameterize detection via repeat measurements within a given timeframe. Future work could explore how much data might be necessary.

Another potential direction for future work is to use causal inference methods to study whether MPA status causes differences in activity levels over time. MPA Watch constitutes a quasi-experiment [40]: sites were not assigned MPA status (the treatment) randomly. Because MPA Watch began after the establishment of MPAs, there are not pre-treatment data with which to measure a post-treatment effect of MPA assignment. However, each MPA has a different date of establishment, so creating a variable to represent how long the location has been protected could be fruitful as a way to approach the question of the impact of protection. In addition, ongoing MPA Watch data collection does enable year-on-year comparisons in trends between MPA and non-MPA sites, as well as estimation of effects of policy interventions or events (using methods such as regression discontinuities, difference-in-differences [41] or synthetic control [42]). In addition to enabling new inferences, we believe implementing a causal inference approach parallel to our present findings would provide a valuable check on the results. Moreover, asserting a causal model would potentially make results easier to interpret and guide tests for compatibility between the model and the data (e.g., placebo tests and simulation studies). All that said, even without formal causal inference, the consistent patterns across space (MPA vs. non-MPA) and the declining trends in some consumptive activities provide compelling evidence for a treatment effect.

One potential application of causal inference with MPA Watch data would be to investigate the impact of the COVID-19 pandemic in 2020 on human activity at coastal sites. Though we have not found any studies focused specifically on California’s MPA network, the complex impacts of COVID-19 on visitation rates and recreation patterns have been explored in many conservation contexts. Some studies found significant increases in visitation rates due to the pandemic (e.g., [43]) though for many others the effects were mixed, based on a wide range of factors (e.g., [44]), including dynamics related to both distance from population centers and racial identity [45]. Contrasting with increased overall visitation rates, one global study suggested a marked decline in recreational fishing [46]. With COVID-19 serving as a source of a temporal regression discontinuity or other causal inference “experiment” along the entirety of the California Coast, analysis of MPA Watch data could support answering a range of questions about coastal activities related to COVID-19 policies, while accounting for population density, amenities, weather, and other factors suggested by the studies cited above. Such an analysis could also examine the extent to which activity levels have returned to a pre-pandemic baseline.

### Collaborative modeling process

A key feature of this process was the collaborative approach. Co-production of science is widely acknowledged as a time- and resource-intensive approach to science, which can yield benefits such as increased trust among collaborators, improved shared understanding, and greater salience and utility of the resulting scientific products for management, decision making, and other purposes [47–50]. Our own experience on this project aligns with this broader literature. The technical and analytical components of this work would have been of far lower quality without iterative engagement with the organizations responsible for implementing MPA Watch, and far less useful to both MPA Watch and MPA management partners.

Some examples of how our analysis was shaped by these collaborative processes, specifically with the community science managers, include how we handled potential violations and whether we included hand collection of biota as a separate category. Our approach to analyzing potential violations resulted from many conversations, including discussions of the on-the-ground realities of conducting an MPA Watch survey, the variation in surveyor practices with respect to potential violations (e.g., whether they consistently reported them to the authorities), and realities of how this is reflected on individual data sheets and in the centralized database. After these conversations, we adopted the method of focusing on dividing MPAs into categories around allowed level of consumptive activities (“non-MPAs,” “no-take,” and “partially-protected” with some take allowed). Delineating MPAs that do not allow consumption at all yielded a consistent way to identify potential violations (which could then later be checked against the surveyor’s identification of potential violations). In addition to this more general approach to potential violations, we initially only considered fishing (onshore and offshore) in the consumptive activity analysis. After discussions with our community science partners, we discovered that boat based counts also included hand collection along with onshore fishing (which were separate categories on the shore-based data sheet). In response, we tried several different ways of handling this inconsistency in the models. In the end we chose not to include boat-based counts for onshore fishing activities, but because we had investigated hand collection, we continued to include it in our analysis of shore-based counts – and hand-collection turned out to be quite relevant at specific sites like Asilomar and Carpinteria (see next section).

Other aspects of the collaborative process focused on decision makers’ needs. For example, we had discussions around what kinds of specificity and uncertainty were useful or tolerable to state agencies with a direct interest in MPA enforcement and compliance. In general, our experience on this project aligns with general observations that decision makers require iterative engagement with new scientific tools and data products in order to know how they might be useful – and by the same token, scientists must iteratively engage to better understand decision making processes [51]. This may be especially true with respect to human dimensions research in contexts where ecological and biological approaches have been dominant, and building social science expertise is a persistent challenge [52]. In the specific case of California’s MPAs, human dimensions research and monitoring has been mostly focused around detecting impacts on fishing, and broadening this approach to other topic areas has been highlighted as a priority for management agencies [53]. This creates an opportunity for iterative dialog about how MPA Watch can be useful to MPA managers and other partners (e.g., what decisions or management issues to target, or what spatial scales to use), and how to support iterative dialog over time.

The very process of collaborative, participatory modeling generated additional challenges and benefits. Each MPA Watch program already produces a report twice a year showing their data, but our analysis is the first time the managers have engaged with the data aggregated across the state. The complexity of the data meant that it was difficult for the group collecting it to engage effectively with it, particularly at a synthetic, statewide level – and even with an experienced collaborative modeling researcher (Eitzel), the data and the resulting model’s complexity made grasping the results difficult. In the end, however, working through the process together resulted in greater understanding of the potential for the dataset to speak both to specific sites and to statewide patterns. In addition, the iterative process did mean that we often thought we had resolved a particular problem or made a modeling choice that we then found ourselves revisiting later, even up to the end of the analysis phase. This underlines the need for patience and longer timelines in this kind of participatory modeling. Finally, this statewide analysis raised some technical questions regarding the drift of the protocol over time as different programs have begun to train surveyors slightly differently. In the short term, our ability to include random effects for site, transect, and program allowed us to absorb some of these differences. And in the long term, our iterative analysis reinforced the need for the community science managers to realign their protocols (a process they have engaged in already, but one whose importance was re-emphasized by this analysis). These protocol alignment discussions have also highlighted the point that the California coast is wildly varied from north to south, not just in terms of activities, population, and infrastructure but also in terms of geology, ecology, and weather – demonstrating some of the underlying reasons for the challenges inherent in a statewide analysis.

### Implications for conservation

One can find calls for increased integration of social science approaches into conservation going back decades [54,55]. There has been a significant push in this direction in recent years, led by interdisciplinary scholars who focus on both the many kinds of social science that can inform conservation, and the co-production processes that are needed for better informing management and effectively integrating with natural science [56–58]. Here we examine the specific implications of our study for conservation in the context of MPAs in California.

There are many ways that social sciences in general, and MPA Watch data in particular, may inform aspects of MPA governance – beginning with the high level goals laid out in the Marine Life Protection Act (MLPA) that set out a requirement for agencies to establish an MPA network in state waters. These goals point to the protection of “natural diversity and abundance of marine life, and the structure, function and integrity of marine ecosystems,” and the improvement of “recreational, educational and study opportunities provided by marine ecosystems that are subject to minimal human disturbance, and to manage these uses in a manner consistent with protecting biodiversity” [26]. An understanding of both the levels and types of activities at varying spatial and temporal scales can inform management toward these goals. Furthermore, the governance of MPAs has evolved to include significant networks of partnerships and community involvement [28,59], as well as a focus on diversity, equity, and inclusion. [29] MPA Watch data can potentially speak to these issues, while also impacting them through the community-engaged approach that the program embodies – not just recording activities at the coast, but also increasing awareness of and engagement with MPAs among participants and their communities [33,60]. This reciprocal relationship between monitoring through participatory science, and the ecological/management goals that drive that approach, is a key part of the state’s strategy with respect to MPAs [29].

In addition, as we noted earlier, one major ocean conservation question revolves around resource use and recreation. MPA Watch data demonstrates that the vast majority of activity on the California coast is non-consumptive, and although fishing is an important livelihood and pastime, it is not the dominant activity even in unprotected places. For example, Carpinteria in Southern California (a non-MPA site) has a large amount of total activity: the model predicts over 450 people total on a day with clear weather in a popular time of year, and for consumptive activities, the model predicts 2.5 people hand-collecting, 1.9 people fishing onshore, and 0.1 boats offshore fishing. By contrast, the models predict 324 people participating in onshore and 35 participating in offshore recreation (in addition to other non-consumptive uses; see S1 Appendix). We observed these patterns in model predictions across many sites. Therefore the model, after correcting for other possible biases in sampling, confirms the big-picture pattern of overwhelmingly non-consumptive activity, which we observed in the raw data in our earlier analyses [25]. This result, while not surprising at a high level, lends a quantitative entrypoint for both understanding this relationship between consumptive and nonconsumptive uses, and monitoring it over time. Our results also demonstrate that people are recreating in MPAs at the same rates as in other non-MPA sites. This result implies that when people are engaging with protected areas, there is no evident statewide negative effect of the protected areas on recreation patterns, suggesting that in this case, protecting the subtractable resource of the commons (e.g., fishing/consumption) does not, at the statewide level, interfere with the non-subtractable (e.g., recreation/tourism) uses [61]. This is directly relevant to the management goal of state agencies of enhancing recreational opportunities and reflects a key management success: protection has been applied without deterring public enjoyment.

Finally, compliance with MPA regulations is another major interest of state agencies and other partners working together to implement MPAs (research question Q3). For any discussion of compliance, it is important to note that MPA Watch data only reveal “potential” violations (volunteers are not trained in law enforcement, and as a rule do not pursue investigations of any potential violations they observe). With that said, our analysis suggests that overall compliance with the Marine Life Protection Act is quite high: the vast majority of counted activities do not appear to violate MPA regulations. However, there may still be places where attention is warranted to more outreach and enforcement. In another recent innovative use of MPA Watch data [62], we can see that some “charismatic” sites do appear to attract disproportionate numbers of visitors for certain biodiversity-related activities. When considering possible changes to the network of MPAs (as is currently happening in the case of California MPAs), MPA Watch data can thus yield helpful “big picture” perspectives, while also offering the ability to zoom in on very specific places or questions. Our analysis reveals particular locations where potential violations are higher, either relative to rates of other activities at that location, or relative to potential violation rates at other sites. Even in these cases, the scale of the problem is small relative to the overall coastal use at those locations, but these results may still be useful in shaping outreach, education, and enforcement activities.

**Fig 5:**
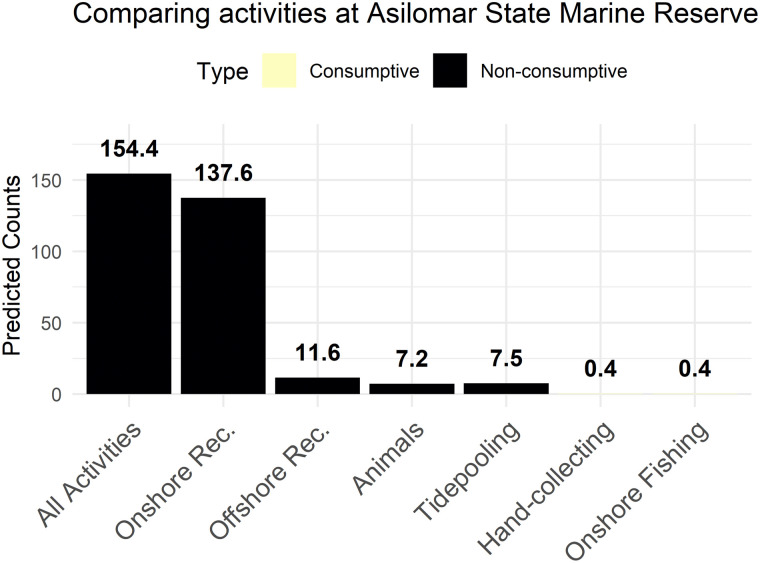
Counts across all activities for Asilomar, showing the relative proportion of different activities. (Recreational boating and offshore fishing not shown because they are counts of boats rather than people.).

Take, for example, Asilomar State Marine Reserve in central California (Fig 5. Similar to Carpinteria, discussed above, the vast majority of activities are non-consumptive. The model predictions show approximately 154 people there (for all activities) on a day with clear weather conditions in a popular time of year, and under those same conditions, the models predict 7 people tidepooling and only 0.4 people hand-collecting. In Carpinteria, 6 people are predicted to be tidepooling, and its lack of protection is evident in the prediction of 2.5 people hand-collecting (relative to Asilomar’s 0.4). Therefore while the presence of hand collection implies a potential need for additional management action, existing efforts do appear to have reduced the relative amount of this activity. Asilomar also has one of the highest predictions for onshore fishing of a no-take MPA, but this is only 0.4 people as well. So while further outreach and/or enforcement might be warranted, the scope of the problem is small relative to overall activities at these sites. Generally, comparing the sites directly shows that domestic animal activity and tidepooling are much higher at Asilomar, and fishing and hand collecting are higher at Carpinteria (see S1 Appendix) – appropriate to their protection status.

These results also highlight that MPA Watch data can be used to help tailor stewardship efforts at specific sites: generally speaking, comparing the different models for different activity categories reveals that some sites have particularly high counts for specific activities, and this could be useful information for MPA managers or other decision makers – whether or not the site has legal restrictions. For example, results could help with discussion about signage (an area of ongoing interest [63]), or the allocation of other public education resources. The existing data and models can also be used as a starting point for moving from studying activities of coastal users to also investigating their attitudes towards MPAs: with support from the California Ocean Protection Council the MPA Watch programs are developing a complementary intercept survey protocol to add to the existing activity count protocol. This intercept survey, now entering a second phase of testing, will allow surveyors to approach coastal users and ask them about their reasons for being at the coast, barriers they may experience in accessing these spaces, and their knowledge of MPAs in California and locally. This process allows managers to better understand motivations for a subset of the large number of people who come to California’s coast, in order to inform a broader agenda of understanding the human dimensions of Marine Protected Areas. The two study protocols together can help inform both bigger patterns as well as understanding specific sites, supporting management both at statewide and local scales.

## Conclusion

Our analysis demonstrates the potential of both large-scale participatory science data collection and the use of appropriately complex statistical models to analyze that data. Further, the analysis is enabled and enhanced by the involvement of the data collectors with the statistical analysis process. Through this iterative, collaborative process, we were able to show that within protected areas, very few consumptive activities occur but there is still robust non-consumptive human presence, and that non-consumptive activities are the majority of activities along the California coast more generally. These conclusions could not be drawn without the hard work of more than a decade of individuals contributing data, nor without the ability to apply statistical models to synthesize this complex source of information. Our study supports the need for collaborations between modelers/statisticians, large-scale participatory science data collection efforts, and decision-makers – underlining what is possible when we work together on these ambitious projects.

## Supporting information

S1 Appendix Additional methods and results. This appendix includes detailed descriptions of the predictor variables, alternative modeling approaches considered and tried, and detailed tables and figures of results for all activity categories.
(PDF)
