# Supplementary material for: Demonstrating conservation impacts in California Marine Protected Areas using large-scale participatory science data
Source: PLoS One. 2026 Jun 25;21(6):e0352449. doi: 10.1371/journal.pone.0352449 (PMC13298793; doi:10.1371/journal.pone.0352449)
Supplement: S1 Appendix — This appendix includes detailed descriptions of the predictor variables, alternative modeling approaches considered and tried, and detailed tables and figures of results for all activity categories. (PDF) [file pone.0352449.s001.pdf]

Appendix Materials: Demonstrating conservation impacts in California Marine Protected Areas using sophisticated statistical analysis of large-scale participatory science data

MV Eitzel[1,2*], Nick Ulle[3], Ryan Meyer[2], Ben Goldstein[4]

1 Feminist Research Institute, University of California, Davis, Davis, CA, United States of America
2 Center for Citizen and Community Science, University of California, Davis, Davis, CA, United States of America
3 DataLab, University of California, Davis, Davis, CA, United States of America
4 Department of Forestry and Environmental Resources, North Carolina State University, Raleigh, NC, United States of America

The goal of this model is to answer questions about Marine Protected Area (MPA) use across the state of California during 2012-2020. Surveyors collect data on human activities in MPAs following a set protocol, but sampling effort is highly variable across space and time, and some activities may not be observed in a given survey.

This appendix includes detailed descriptions of the predictor variables, GLMM implementation, alternative modeling approaches considered and tried, and detailed tables and figures of results for all activity categories.

Details on predictor variables

For all continuous predictors, we standardized the variables (subtracted the mean and divided by the standard deviation) in order to improve the optimizer's ability to find a maximum likelihood. For some of the continuous variables, we included higher order polynomials to allow for more flexible functions of these variables. Based on residuals in DHARMa, most activity categories had only the linear and quadratic terms for e.g. latitude but animals and recreational boating benefited from up to 8th order polynomials.

Access/Amenities Index

For each transect, a variety of variables accounted for the amenities and access characteristics of each location. We constructed an index representing greater or lesser amenities and access, as follows:

For each of the following variables, we added one to the index: "has_development," "has_camping," "has_paths," "has_restrooms," "has_lifeguard," "has_harbors," and "has_jetties." We then added 1 if there was a fee parking lot, 2 if there was fee and free parking, and 3 if there was free parking (0 indicated no parking lot). We then added 2 if there was street parking within a quarter mile of the access point closest to the start of the transect, and 1 if it was greater than a quarter mile away (0 indicated no street parking). Finally, we added 1 for transects with access described as "one point," 2 for "multiple points," and 3 for "primarily open."

Seasonality: "Winterness" and "Springness"

Because seasonality is cyclical, we decomposed the time of year into polar coordinates, by taking the sine and cosine of the proportion of the year that has passed. For any given survey i, we define "winterness" as:

$$[\text{winterness}]_i = \cos\left(2\pi \cdot \frac{[\text{day}]_i + 11.24}{[\text{days in year}]_i}\right)$$

And "springness" as:

$$[\text{springness}]_i = \sin\left(2\pi \cdot \frac{[\text{day}]_i + 11.24}{[\text{days in year}]_i}\right)$$

Where $[\text{day}]_i$ is the day of year on which the survey was collected, and $[\text{days in year}]_i$ is the total number of days in the survey year (accounting for leap years). The offset 11.24 was selected to make winterness and springness have approximate values 1 and 0, respectively, on the winter solstice. Note that these variables range from -1 to 1.

Tide level

We computed a unitless tide level estimate for each survey from the U.S. National Oceanic and Atmospheric Administration's (NOAA) Center for Operational Oceanographic Products and Services (CO-OPS) tide station data[1]. Note that this variable is already unitless and standardized (within a given tide station), so it is not centered and scaled like the other continuous predictors.

We used noaaoceans (version 0.3.0)[2] to collect the locations of tide stations in California active from 2012–2020. We then computed the geospatial distance from each MPA and non-MPA site to each tide station, and used this to match each site (and thereby survey) to its nearest tide station. Next, for each survey, we computed the midpoint between the survey start and end time, and matched it to the nearest tide measurement time (tide measurements are taken hourly) at its matched tide station. We then used noaaoceans to collect the tide height for each survey at the matched tide station and time. In the event that a survey's nearest tide station did not have data for the matched time, we used the second-nearest tide station instead.

Tidal range can vary substantially even between locations within a few miles of each other, so in order to allow comparisons between tide heights at different sites, we centered and scaled each measurement.[3] For each tide station, we collected the diurnal tide level estimate and the great diurnal range estimate for the 1983-2001 National Tidal Datum Epoch, the most recent long-term estimation period as of this report. The diurnal tide level is the mean of the daily midpoint of higher high tide and lower low tide. The great diurnal range is the mean of the daily difference between higher high tide and lower low tide. For each tide height measurement, we subtracted the corresponding station's diurnal tide level and divided by the corresponding station's great diurnal range. As a result, the tide level variable is unitless and ranges from approximately -1 (lowest low tide) to 1 (highest high tide).

[1] U.S. National Oceanic and Atmospheric Administration (2022). Center for Operational Oceanographic Products and Services Data. URL https://api.tidesandcurrents.noaa.gov/api/prod/.

[2] Warlick S (2021). noaaoceans: Collect Ocean Data from NOAA. URL https://CRAN.R-project.org/package=noaaoceans.

[3] This process is different from the centering and scaling of the other continuous variables. Here, the measurement is centered and scaled within the single tide station.

Population Density

We computed estimates of nearby population density for each MPA and non-MPA site from the U.S. Census Bureau's American Community Survey (ACS) 5-year Data[4]. The ACS 5-year Data provides population estimates for each census tract in the U.S. based on 5 years of historical data.

We used tidycensus (version 1.0)[5] to collect ACS 5-year population estimates for each census tract in California and each year in 2012-2019. We then converted these into population density estimates (in persons per square kilometer) by dividing by each tract's land area. For each MPA and non-MPA site, we identified all census tracts within 15 miles of the site's boundaries. Finally, we computed the mean of the population density estimates over the identified tracts and 2012-2019, to get one mean for each site.

Survey Duration

To compute survey duration, we subtracted the survey end time from its start time. We found 17 surveys had duration greater than 3 hours and 222 had zero or negative duration. We believe both cases are due to errors during data entry. Surveys are intended to take only 1 hour, so we capped all duration measurements at 3 hours. We replaced zero or negative duration with the median duration (25 minutes) of the surveys with non-erroneous duration. These alterations collectively represent 0.8-0.9% of the surveys.

Distance to a state park

We computed a binary variable indicating whether each site is adjacent to a California State Park in two steps. First, we obtained official park boundaries from the California State Parks GIS Data portal and computed the great circle distance to the nearest park boundary for each site. We then marked all sites with distance 0 kilometers as adjacent and all other sites as non-adjacent.[6]

Generalized Linear Mixed Model implementation details

We used Generalized Linear Mixed Models (GLMMs) to model counts of each human activity type. We modeled each activity category separately, following a negative binomial distribution, which can represent counts with dispersion using two different parameterizations (linear and quadratic). We included all predictor variables appropriate for each activity category in the "conditional" part of the model represented by the counts.

We estimated models using glmmTMB (version 1.1.10)[7] in R (version 4.4.2)[8]. We ensured that our results come from models with no convergence warnings, indicating that the optimizer successfully found a maximum. To assess model fit and choose between model parameterizations,

[4] U.S. Census Bureau (2020). 2012-2019 American Community Survey 5-year Data. URL https://www.census.gov/data/developers/data-sets/acs-5year.html.

[5] Walker K, Herman M (2021). tidycensus: Load US Census Boundary and Attribute Data as 'tidyverse' and 'sf'-Ready Data Frames. URL https://CRAN.R-project.org/package=tidycensus.

[6] State park boundaries retrieved from `https://www.parks.ca.gov/?page_id=29682` in April 2023. The metadata says they are "Current as of February 28, 2022."

[7] Brooks ME, Kristensen K, van Benthem KJ, Magnusson A, Berg CW, Nielsen A, et al. glmmTMB Balances Speed and Flexibility Among Packages for Zero-inflated Generalized Linear Mixed Modeling. The R Journal. 2017;9(2):378–400. doi:10.32614/RJ-2017-066.

[8] R Core Team. R: A Language and Environment for Statistical Computing; 2025. Available from: https://www.R-project.org/.

we used DHARMa (version 0.4.7)[9] to plot model residuals. We used iterative graphical checks to assess whether there were systematic deviations between the model and the observed data while selecting error families and the form of zero-inflation (see below). While the iterative process was largely qualitative, we made sure to choose models that had non-significant tests for zero-inflation and dispersion (implying that we had accounted for these statistical features within the models we had chosen). For some of the continuous variables, we included higher order polynomials to allow for more flexible functions of these variables; how many terms to include was based on observations of patterns in the residuals.

For our key questions regarding activities inside and outside MPAs based on the MPA's type of restriction, and the potential patterns across time, we also included an interaction between site type and year, in order to see how usage patterns may have changed differently over time for each category of site (no-take MPAs, some-take MPAs, and non-MPAs).

Dispersion & zero-inflation

By using a negative binomial distribution, we also able to account for overdispersion in observed counts, when there is more or less variation in counts than would be expected for a typical distribution (e.g. Poisson). In addition, predictor variables can be included in the zero-inflation and/or dispersion parts of the model – reflecting that some categories of sites have even more zeroes than others, or some categories have greater variation than other categories. For most activities we had only a constant overdispersion parameter. The meaning of the overdispersion parameter is different for different forms of the negative binomial distribution; we clarify which distribution was used for each activity category in the results table on dispersion and zero-inflation. We did consider models with predictors in the overdispersion portion of the model, but for most activity categories, the residuals were not much improved, so we chose to keep those models simpler. For hand collection of biota, we did include the beach type and whether it was a known tidepooling site in the overdispersion model. This reflects that surveys with different values of these predictors had higher or lower variation than others (for example, there could be more variation for a rocky transect than for a sandy one).

We also modified GLMMs to account for zero inflation, where the data include additional zero observations beyond what might be expected from the assumed distribution. For example, additional zeroes in MPA Watch surveys could be due to mismatch in timing between surveyors and coastal users (surveyors simply are not present when individuals are engaging in activities), and/or because surveyors check for all activities every time they survey and they could be doing so at a time or place which is appropriate for one activity but not another.

For most activity categories, we had a constant zero-inflation parameter, which means that there is a constant probability that a survey recorded 'extra' or 'structural' zeroes e.g. due to mismatch in timing between surveyor and activity – in addition to the underlying distribution of counts which could include some zeroes as part of the underlying process. For tidepooling, we did include predictors in the zero-inflation part of the model, which means that for example, sites that were not known for tidepooling were more likely to have zero tidepoolers than a site that was known for tidepooling (in addition to the fact that there might not be any tidepoolers at that site in that particular moment, e.g. if it were a less popular site for tidepooling, with lower counts overall). Onshore recreation, which is a much more common activity than the others that was essentially always present, was not zero-inflated.

[9] Hartig F. DHARMa: Residual Diagnostics for Hierarchical (Multi-Level / Mixed) Regression Models; 2024. Available from: https://CRAN.R-project.org/package=DHARMa.

Random effects

For all activity categories, we included nested Gaussian random effects for site and transect within site. These random effects are intended to account for additional unknown factors that could differ from transect to transect or site to site that were not accommodated by the other covariates in the model. We also included random effects for MPA Watch program (13 programs which include multiple sites; we are accounting for the possibility that programs may train volunteers in slightly different ways). We excluded the MPA Watch program random effect in four cases (offshore fishing, recreational boating, and animals), as including all three random effects resulted in overfitting (as observed in the DHARMa residuals).

Statistical inference

We used Wald tests to determine the statistical significance of the continuous predictor covariates and categorical variables with only two levels (e.g. yes/no) in the count, dispersion, the zero-inflation portions of the model. We used R's built-in "drop1" function to do likelihood ratio tests for categorical variables with more than two levels (e.g. three different MPA types or days of the week) and Tukey tests (R function "glht" in package "multcomp" (version 1.4.28)[10] for multiple comparisons between different levels of categorical variables (e.g. the differences between days of the week and differences between MPA types). Because this resulted in 624 tests total for all analyses, we applied a multiple testing correction to all p-values WITHIN a given activity category (not across all 624 tests) using the Benjamini-Hochberg "false discovery rate" correction.[11] This reduces the possibility that a particular test would appear significant by chance.

Aggregated Total Activities

For the "Total Activities" category, which includes all the other categories listed in the main manuscript as well as other smaller categories that were too rare to be separately modeled, we display visual summaries of modeling results, but do not calculate statistical results. This category is included for illustrative purposes and to give context to the magnitude of the other category model predictions but in order to preserve the validity of p-values, we do not draw statistical conclusions regarding it because it includes data that have already been used for inference.

Inputs for predictive figures

For all of the figures showing model results, we used the 'predict' function in R. This required input values for all of the predictor variables, which we list below:

For the time trends/MPA type, seasonality plots, and daily/diurnal plots, these are the inputs (if the variable was included for that activity category):

- Average access/amenities index
- Average population density
- Clear weather: no clouds, no precipitation, and perfect visibility

[10] Hothorn T, Bretz F, Westfall P. Simultaneous Inference in General Parametric Models. Biometrical Journal. 2008;50(3):346–363. doi:10.1002/bimj.200810425.

[11] Benjamini Y, Hochberg Y. Controlling the False Discovery Rate: A Practical and Powerful Approach to Multiple Testing. Journal of the Royal Statistical Society: Series B (Methodological). 1995;57(1):289–300. doi:10.1111/j.2517-6161.1995.tb02031.x.

- Average tide level
- Average transect, site, and latitude (corresponding to the middle of the state)
- Noon on Summer solstice on a Saturday in 2016
- A Land-based one-mile-long transect with an hour-long survey
- A Sandy transect with known tidepooling adjacent to a State Park

For the transect-level (spatial/geographical) plots, the same inputs were used for the weather, tide level, survey duration, and timing, but the MPA type, access/amenities, population density, beach type, tidepooling status, adjacency to State Parks, transect length, survey type (land/boat), transect, program, site, and latitude were drawn from the data.

Alternative modeling choices

N-Mixture models

In addition to GLMMs using glmmTMB, we considered N-Mixture models in NIMBLE[12,13] and JAGS.[14] These models are similar to our previous occupancy model, in that they explicitly model a detection probability as well as an underlying count or abundance. However, the assumptions of these models were not satisfied for our data. In particular, the assumption of 'closure' (that an individual is always present within the temporal/spatial unit of analysis and the count is therefore constant in that unit of analysis[15]) is difficult to justify for our data for the temporal/spatial units of analysis necessary to also guarantee multiple observations in order to estimate detection probability. In addition, glmmTMB allows for more easily incorporating dispersion and zero-inflation. We also tried Poisson and negative binomial distributions in other R packages for comparison (e.g. lme4, glmer, glmer.nb) and found glmmTMB to be the most flexible.

Handling duplicate records

The other alternate modeling choice we tried was two different ways to handle duplicate surveys. There are approximately 400 records that are duplicated in the dataset, and at least some of them are true duplicates: For some surveys, teams of surveyors went out together and divided up the tallying task, then aggregated their counts into one data sheet. For a subset of these, all the surveyors submitted their own individual copies of that same survey, duplicating the records for that transect at that time. This is not always true, however, and addressing these discrepancies would involve either a case-by case approach, or a more sophisticated automated way of resolving them. Instead, we fit the models both with and without these duplicates. The results in the main

[12] de Valpine, P., D. Turek, C.J. Paciorek, C. Anderson-Bergman, D. Temple Lang, and R. Bodik. 2017. Programming with models: writing statistical algorithms for general model structures with NIMBLE. Journal of Computational and Graphical Statistics 26: 403-413. ¡DOI:10.1080/10618600.2016.1172487¿.

[13] de Valpine P, Paciorek C, Turek D, Michaud N, Anderson-Bergman C, Obermeyer F, Wehrhahn Cortes C, Rodrìguez A, Temple Lang D, Paganin S (2022). NIMBLE User Manual. https://doi.org/10.5281/zenodo.1211190, R package manual version 0.12.2, https://r-nimble.org.

[14] Plummer, M. (2003). JAGS: A Program for Analysis of Bayesian Graphical Models Using Gibbs Sampling. Proceedings of the 3rd International Workshop on Distributed Statistical Computing (DSC 2003), Vienna, 20-22 March 2003, 1-10.

[15] Kéry, M. & Royle, J.A. 2021. Applied hierarchical modeling in ecology—Modeling distribution, abundance and species richness using R and BUGS.

manuscript INCLUDES all the duplicates, but when removing them and re-running the models, there were only small changes. A handful of pairwise comparisons changed significance level; some of the random effects, zero inflation, and dispersion parameters changed typically in the third decimal place; and predicted peak times of day were largely unchanged but onshore fishing changed about 15 minutes, and predicted peak times of year often shifted by a day, or were unchanged. Based on the small degree of change (particularly at the level we ultimately summarized in the main paper), we chose to keep the data minimally altered and included the duplicates in our analysis.

Detailed results

We first share tables summarizing results for all variables for all activity categories, and then below we include figures showing all activity categories, and more detailed tables including parameter estimates, standard errors, p-values, and corrected p-values for all predictors for all activity categories.

Summary tables for all variables

Tables 1- 6 summarize the results for each activity. 'NS' indicates not significant ($P > 0.05$), while an empty cell indicates that variable was not included for that activity category. '(+)' indicates a positive effect, while '(-)' indicates a negative effect.

Table 1: **Results for transect- and site-related features (Q5).** Note that 'beach type' was not included in offshore fishing or recreational boating, and whether the site was a known tidepooling site was only considered for onshore recreation, tidepooling, and hand collection of biota. The access and amenities index, population density, and adjacency to a State Park were all considered for all activity categories.

Activity category	Access/ amenities	Beach type	Tidepooling	Population density	State Parks
Onshore Recreation	(+)	Sandy/Rocky > Rocky Sandy > Rocky	NS	NS	NS
Offshore Recreation	(+)	Sandy/Rocky > Rocky Sandy > Rocky		NS	NS
Onshore Fishing	(+)	NS		NS	(+)
Offshore Fishing	NS			NS	(-)
Tidepools	NS	NS	(+)[1]	NS	NS
Recreational Boating	NS			(+)	NS
Domestic Animals	(+)	Sandy/Rocky > Rocky		(+)	NS
Hand Collection	NS	NS	NS[2]	NS	NS

[1] The 'known tidepooling site' factor was not significant in the zero-inflation term, but we included it in the final models for theoretical reasons and due to better model fit via evaluation of residuals.
[2] The 'known tidepooling site' factor was significant in the dispersion term of the model for hand collection of biota, though not in the count portion of the model.

Table 2: **Results for spatial variables (Q5).**

Activity category	Latitude linear	Latitude squared	Latitude higher-order[1]	Site Random Effect	Transect Random effect
Onshore Recreation	NS	NS		0.8185	0.6867
Offshore Recreation	(-)	NS		1.0925	0.8786
Onshore Fishing	NS	NS		0.5765	0.9386
Offshore Fishing	NS	NS		0.9969	0.7840
Tidepools	NS	NS		1.6945	1.0613
Recreational Boating	NS	(-)	4th (+), 6th (-)	0.8446	0.9779
Domestic Animals	NS	NS	NS	0.9371	0.9556
Hand Collection	(-)	NS		0.8272	1.0005

[1] Domestic animals and recboating had terms up to 8th order, based on residual patterns.

Table 3: **Results for temporal variables (Q4).** For all activity categories, the linear and quadratic terms for time of day were statistically significant; we share an estimate of peak time of day. For our two time-of-year variables, "winterness" and "springness," one or both were significant for all activity categories so we share an estimate of peak time of year.

Activity category	Day of Week	Time of Day	Seasonality
Onshore Recreation	Weekend > Weekday<br>Fri > Tue, Wed, Thu<br>Mon > Thu	2:40 pm	Jul 9
Offshore Recreation	Weekend > Weekday<br>Fri > other weekdays	1:40 pm	Jul 30
Onshore Fishing	Weekend > Weekday<br>Fri > other weekdays	10:30 am	Jun 26
Offshore Fishing	Weekend > Weekday	10:00 am	Sep 14
Tidepools	Weekend > Weekday	2:10 pm	Jul 4
Recreational Boating	Weekend > Weekday<br>Fri > other weekdays	12:10 pm	Jul 27
Domestic Animals	Weekend > Weekday<br>Fri > Tue<br>Mon > Tue, Wed > Tue	4:50 pm	Jul 2
Hand Collection	Sat > Weekday<br>Sun > Mon, Wed, Thu	12:40 pm	Jul 6

Table 4: **Results for survey method variables.** Note that only recreational boating and offshore fishing use both boat and shore-based surveys.

Activity category	Survey Type	Transect Length	Survey Duration	Program Random Effect
Onshore Recreation		NS	(+)	0.4648
Offshore Recreation		NS	(+)	0.6828
Onshore Fishing		NS	(+)	0.0003
Offshore Fishing	Boat > Land	NS	(+)	
Tidepools		NS	(+)	0.4362
Recreational Boating	NS	NS	(+)	
Domestic Animals		Medium-length = higher[1]	Medium-duration = higher[2]	
Hand Collection		NS	(+)	0.2925

[1] For domestic animals, the linear term was not significant, but we also included a quadratic term, which was significant.
[2] Both linear and quadratic terms were significant.

Table 5: **Results for tide level and weather.** Note that all of the weather variables were included for all activity categories, but tide level was not included for offshore fishing.

Activity category	Tide level	Clouds	Precipitation	Visibility
Onshore Recreation	(-)	clear > partly cloudy<br>partly cloudy > cloudy<br>clear > cloudy	(-)	limited > perfect
Offshore Recreation	NS	Clear > partly cloudy<br>Partly cloudy > cloudy<br>clear > cloudy	(-)	limited > perfect
Onshore Fishing	NS	NS	(-)	NS
Offshore Fishing		cloudy > partly cloudy<br>cloudy > clear	NS	limited > shore only<br>perfect > shore only<br>perfect > limited
Tidepools	(-)	clear > cloudy	(-)	limited > perfect
Recreational Boating	NS	Clear > partly cloudy<br>partly cloudy > cloudy<br>clear > cloudy	(-)	perfect > limited
Domestic Animals	(-)	partly cloudy > cloudy<br>clear > cloudy	(-)	perfect > limited
Hand Collection	(-)	cloudy > clear	NS	NS

Table 6: **Results for dispersion and zero-inflation.** Note that Onshore recreation is a common enough activity that it was not zero-inflated.

Activity category	Distribution	Dispersion parameter	Dispersion residual p-value	Zero-Inflation parameter	Zero-inflation residual p-value
Onshore Recreation	nbinom1	29.67	0.608	NA	0.176
Offshore Recreation	nbinom1	18.48	0.264	-3.458	0.696
Onshore Fishing	nbinom1	3.098	0.576	-2.193	0.912
Offshore Fishing	nbinom2	0.3029	0.480	-17.92	0.464
Tidepools	nbinom1	14.18	0.448	-1.398	0.984
Recreational Boating	nbinom1	2.762	0.952	-2.001	0.992
Domestic Animals	nbinom2	1.023	0.056	-16.06	0.880
Hand Collection	nbinom1	2.117	0.128	0.4304	0.888

Figures

We first show the comparative results for Asilomar State Marine Reserve and Carpenteria beach (Figure 1), and then the same calculations normalized by the total predicted activities, and then the ratio of Asilomar to Carpenteria (Figure 2), which shows that animal activity and tidepooling are much higher at asilomar, and fishing and hand collecting are higher at Carpenteria.

We show figures for all activity categories for time trends and MPA effects (Figure 3), seasonal patterns (Figure 4), and daily/diurnal pattern (Figure 5).

Figure 1: Comparison between Asilomar and Carpenteria, with specific predictions from models for each activity.

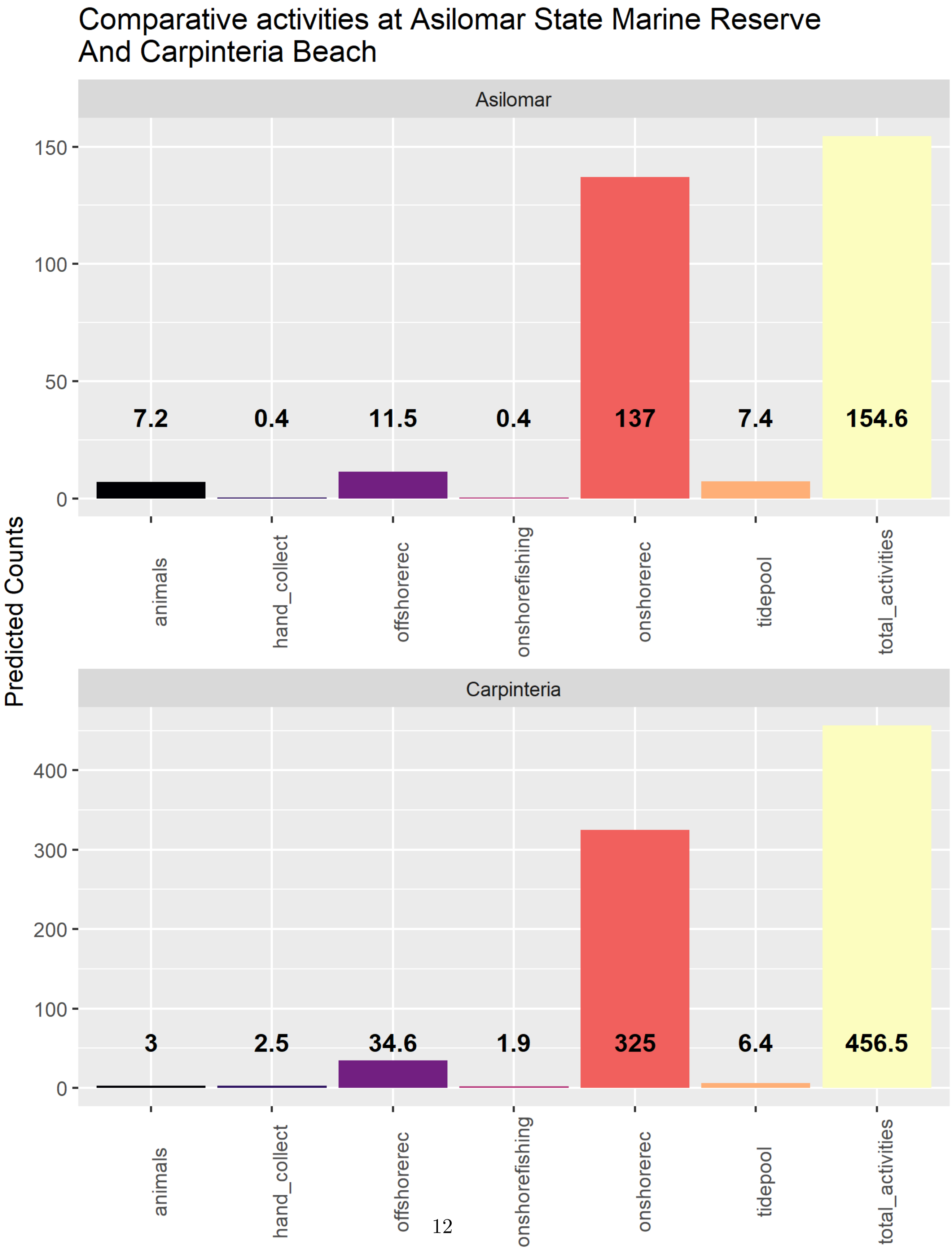

Figure 2: Comparison between Asilomar and Carpenteria, normalized and showing the ratio between the two.

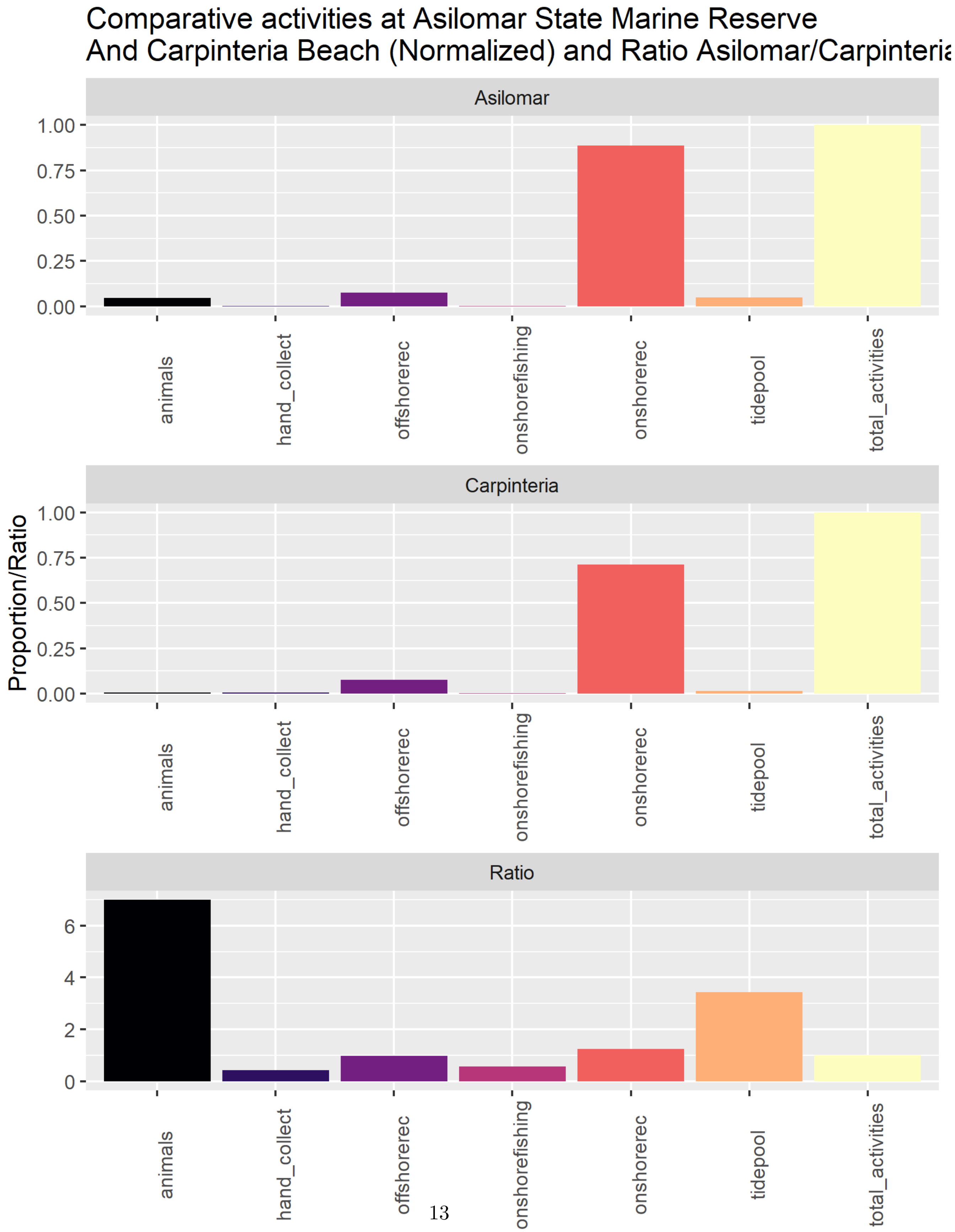

Figure 3: Time trends (days since first survey) for each category of site (No-Take MPAs, “Other” MPAs with some take, and non-MPA sites with effectively “any take.”

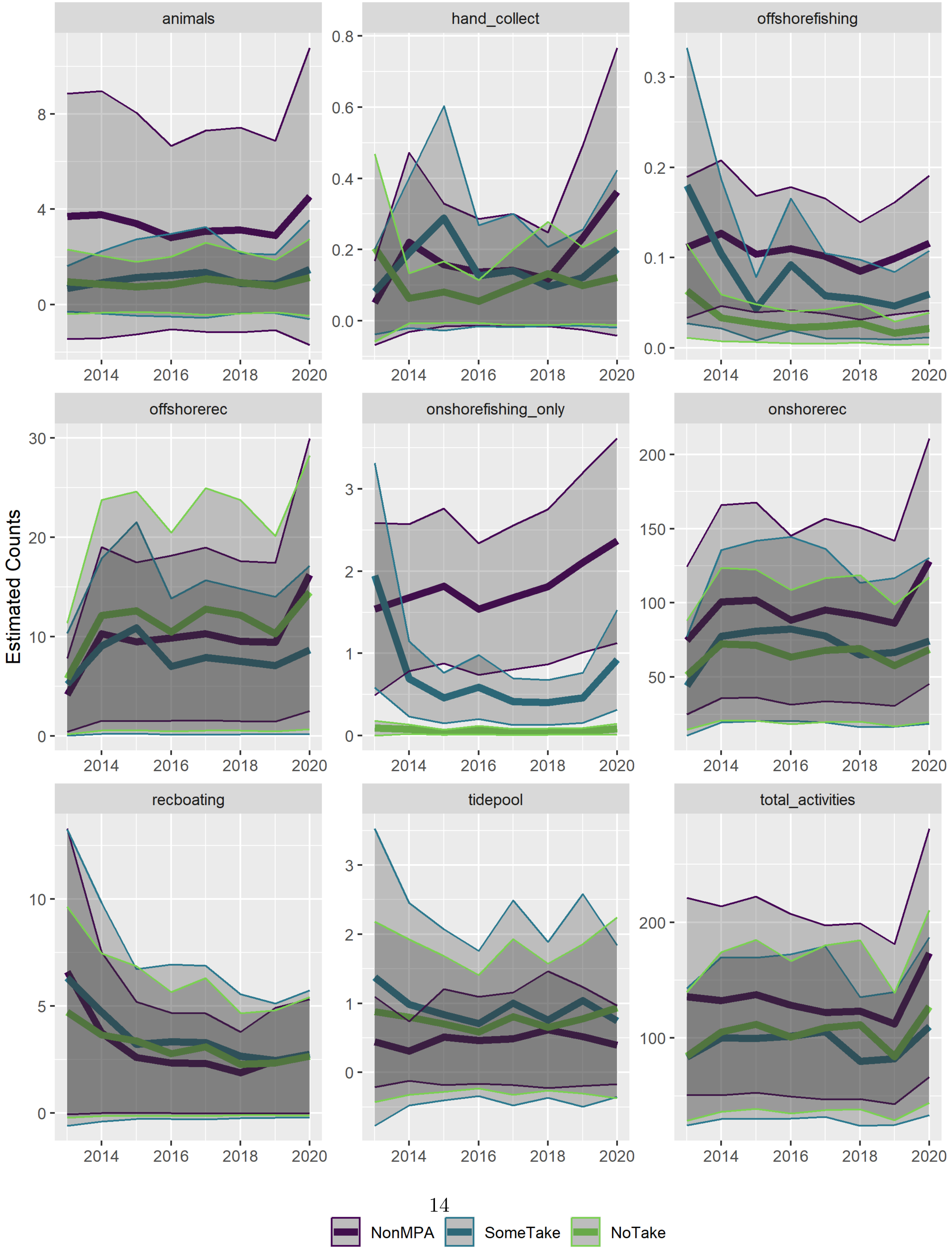

Figure 4: Seasonal patterns for each activity category, with the maximum value marked with a vertical dotted line.

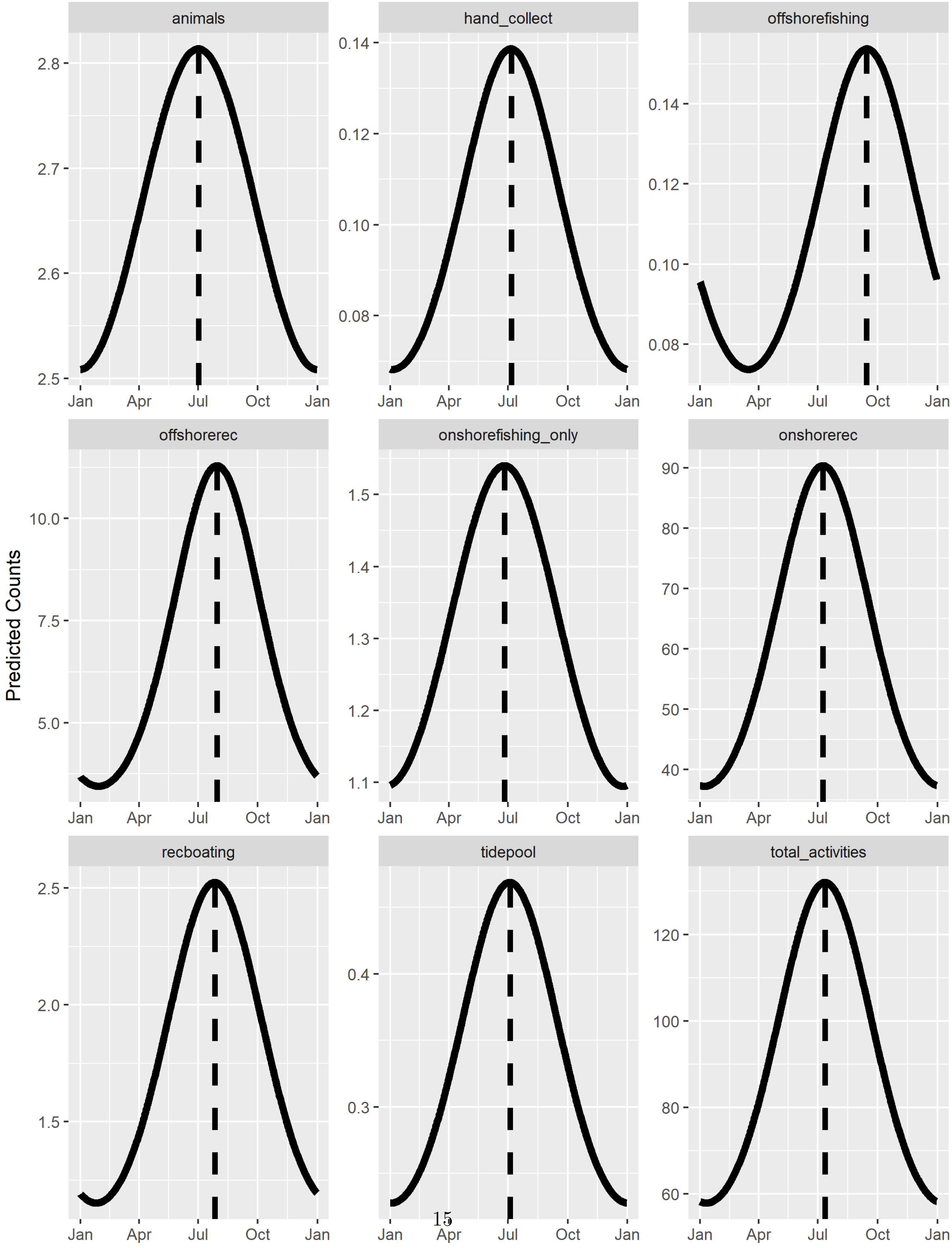

Figure 5: Diurnal patterns of activity throughout the day for each category.

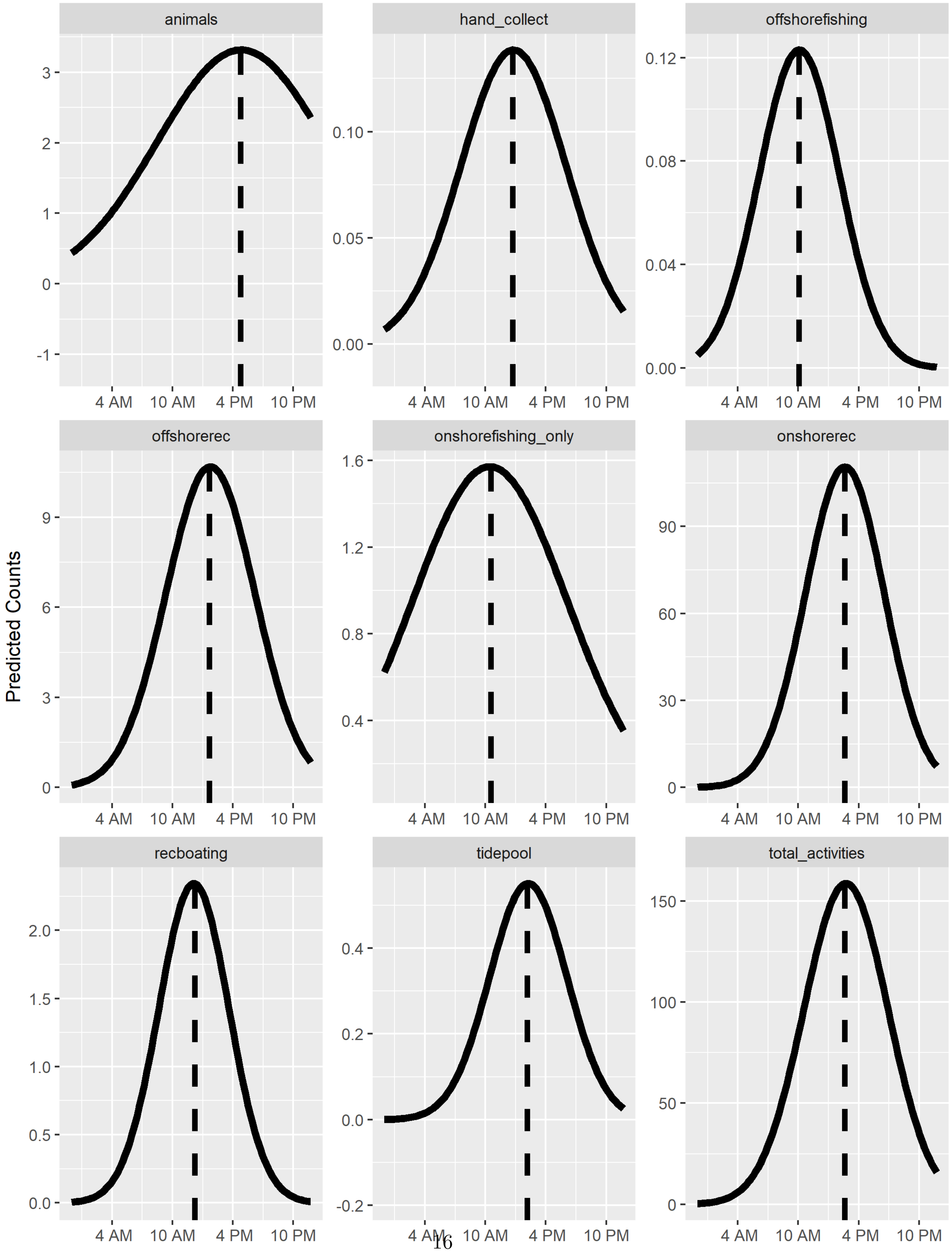

Parameter Estimates and Corrected p-values

Below we summarize p-values for all tests, both uncorrected and corrected, for all activity categories. When a categorical variable has only two levels, R names the variable after the non-reference level, for example, Precipitation's reference level is ''no' so the p-value and parameter estimate are for 'PrecipitationYes.' Parameters for zero inflation have 'zi-' pre-pended to the variable name.

'(Intercept)' is the parameter estimate corresponding to the average amenity value (about 7/14), average population density (about 2,300-2,400 people per square km), a day which is clear, has no rain, and has perfect visibility, average tide height (standardized within each tide station), average latitude (about 35.6 degrees), Saturday, winterness and springness = 0, the beginning of the surveys, an average time of day (slightly after noon), average duration (around 37 minutes, average transect length (just over a mile long), and for a non-MPA site. For categories that include the boat-based surveys (offshore fishing and recreational boating), the reference level is 'land' rather than 'boat' -based surveys. For those that include beach type, the reference is 'Sandy' and for those that include being a known tidepooling site, the reference is 'yes' Because some of these combinations do not make sense physically, we only report parameter estimates in order to compare magnitudes, and when we talk about the effect size, we choose compatible values for these variables using R's 'predict' function (this was the method used for the figures shown in the previous section).

Table 7: P-values for tidepool from glht Tukey test. Bold typeface indicates p<0.05; adjusted p-values have had the Benjamini and Hochberg 'false discovery rate' correction applied to them.

Variable(s)	Parameter Estimate	Standard Error	Raw p-value	Adjusted p-value
NoTake - NonMPA	0.6866	0.7391	0.6217	1.0000
SomeTake - NonMPA	1.1344	0.7934	0.3253	0.7876
SomeTake - NoTake	0.4477	0.7855	0.8360	1.0000
Rocky - Sandy	0.0595	0.5821	0.9940	1.0000
Sand and Rocky - Sandy	0.4224	0.3132	0.3569	0.8188
Sand and Rocky - Rocky	0.3629	0.5538	0.7826	1.0000
cloudy - clear	-0.1945	0.0577	0.0024	**0.0098**
partly cloudy - clear	-0.0750	0.0492	0.2778	0.7222
partly cloudy - cloudy	0.1194	0.0548	0.0744	0.2232
limited - perfect	0.1829	0.0566	0.0030	**0.0116**
shore only - perfect	0.1501	0.1622	0.6053	1.0000
shore only - limited	-0.0328	0.1643	0.9768	1.0000
Sun - Sat	0.0137	0.0623	1.0000	1.0000
Mon - Sat	-0.5083	0.0759	0.0000	**0.0000**
Tue - Sat	-0.6519	0.0811	0.0000	**0.0000**
Wed - Sat	-0.7003	0.0773	0.0000	**0.0000**
Thu - Sat	-0.6121	0.0753	0.0000	**0.0000**
Fri - Sat	-0.5433	0.0763	0.0000	**0.0000**
Mon - Sun	-0.5220	0.0722	0.0000	**0.0000**
Tue - Sun	-0.6656	0.0784	0.0000	**0.0000**
Wed - Sun	-0.7140	0.0743	0.0000	**0.0000**
Thu - Sun	-0.6258	0.0719	0.0000	**0.0000**
Fri - Sun	-0.5570	0.0729	0.0000	**0.0000**
Tue - Mon	-0.1436	0.0853	0.6241	1.0000
Wed - Mon	-0.1920	0.0824	0.2271	0.6109
Thu - Mon	-0.1038	0.0812	0.8602	1.0000
Fri - Mon	-0.0350	0.0818	0.9995	1.0000
Wed - Tue	-0.0485	0.0869	0.9979	1.0000
Thu - Tue	0.0398	0.0859	0.9993	1.0000
Fri - Tue	0.1086	0.0874	0.8759	1.0000
Thu - Wed	0.0882	0.0817	0.9331	1.0000
Fri - Wed	0.1570	0.0833	0.4862	1.0000
Fri - Thu	0.0688	0.0817	0.9801	1.0000

Variable(s)	Parameter Estimate	Standard Error	Raw p-value	Adjusted p-value
2014 - 2013	-0.3546	0.3361	0.9613	1.0000
2015 - 2013	0.1468	0.3250	0.9998	1.0000
2016 - 2013	0.0478	0.3275	1.0000	1.0000
2017 - 2013	0.0966	0.3427	1.0000	1.0000
2018 - 2013	0.3344	0.3346	0.9713	1.0000
2019 - 2013	0.1627	0.3524	0.9998	1.0000
2020 - 2013	-0.1086	0.4125	1.0000	1.0000
2015 - 2014	0.5014	0.1782	0.0823	0.2378
2016 - 2014	0.4024	0.1837	0.3332	0.7876
2017 - 2014	0.4512	0.2133	0.3795	0.8458
2018 - 2014	0.6890	0.2065	0.0168	0.0595
2019 - 2014	0.5173	0.2333	0.3177	0.7876
2020 - 2014	0.2460	0.3197	0.9938	1.0000
2016 - 2015	-0.0990	0.1391	0.9962	1.0000
2017 - 2015	-0.0502	0.1758	1.0000	1.0000
2018 - 2015	0.1876	0.1702	0.9512	1.0000
2019 - 2015	0.0159	0.2050	1.0000	1.0000
2020 - 2015	-0.2554	0.3013	0.9889	1.0000
2017 - 2016	0.0488	0.1735	1.0000	1.0000
2018 - 2016	0.2866	0.1683	0.6623	1.0000
2019 - 2016	0.1149	0.2066	0.9992	1.0000
2020 - 2016	-0.1564	0.3025	0.9995	1.0000
2018 - 2017	0.2377	0.1889	0.9043	1.0000
2019 - 2017	0.0661	0.2274	1.0000	1.0000
2020 - 2017	-0.2052	0.3162	0.9979	1.0000
2019 - 2018	-0.1717	0.2185	0.9930	1.0000
2020 - 2018	-0.4430	0.3097	0.8282	1.0000
2020 - 2019	-0.2713	0.3238	0.9896	1.0000

Table 10: P-values for tidepool from Wald test. Bold typeface indicates p<0.05; adjusted p-values have had the Benjamini and Hochberg 'false discovery rate' correction applied to them.

Variable(s)	Parameter Estimate	Standard Error	Raw p-value	Adjusted p-value
(Intercept)	-1.5902	0.7353	0.0306	0.0993
access	0.1797	0.1474	0.2229	0.6109
popden	0.0769	0.2385	0.7471	1.0000
precipitationyes	-0.7893	0.1515	0.0000	**0.0000**
tide standard	-2.3294	0.0774	0.0000	**0.0000**
lat	-0.9045	0.4105	0.0276	0.0935
lat^2	0.0548	0.2807	0.8452	1.0000
adjacent to parkTRUE	0.4499	0.5345	0.3999	0.8665
winterness	-0.3510	0.0309	0.0000	**0.0000**
springness	-0.0825	0.0321	0.0101	**0.0377**
tod	0.3771	0.0267	0.0000	**0.0000**
tod^2	-0.2791	0.0225	0.0000	**0.0000**
duration	0.4082	0.0243	0.0000	**0.0000**
length miles	-0.0500	0.2470	0.8397	1.0000
has tidepoolingNo	-1.4419	0.3974	0.0003	**0.0012**
zi-(Intercept)	-1.3979	0.1218	0.0000	**0.0000**
zi-has tidepoolingNo	0.5596	0.2939	0.0569	0.1775

Table 12: P-values for onshore fishing from glht Tukey test. Bold typeface indicates p<0.05; adjusted p-values have had the Benjamini and Hochberg 'false discovery rate' correction applied to them.

Variable(s)	Parameter Estimate	Standard Error	Raw p-value	Adjusted p-value
NoTake - NonMPA	-2.8065	0.5142	0.0000	**0.0000**
SomeTake - NonMPA	0.2352	0.4064	0.8302	1.0000
SomeTake - NoTake	3.0418	0.5065	0.0000	**0.0000**
Rocky - Sandy	0.7083	0.3775	0.1405	0.2738
Sand and Rocky - Sandy	-0.1558	0.2328	0.7765	1.0000
Sand and Rocky - Rocky	-0.8641	0.3714	0.0499	0.1149
cloudy - clear	0.0395	0.0444	0.6453	1.0000
partly cloudy - clear	0.0882	0.0402	0.0720	0.1582
partly cloudy - cloudy	0.0487	0.0397	0.4366	0.7214
limited - perfect	-0.0925	0.0492	0.1312	0.2623
shore only - perfect	-0.1885	0.1233	0.2594	0.4585
shore only - limited	-0.0959	0.1282	0.7203	1.0000
Sun - Sat	-0.0076	0.0475	1.0000	1.0000
Mon - Sat	-0.8750	0.0604	0.0000	**0.0000**
Tue - Sat	-0.9590	0.0674	0.0000	**0.0000**
Wed - Sat	-0.9550	0.0647	0.0000	**0.0000**
Thu - Sat	-0.9549	0.0622	0.0000	**0.0000**
Fri - Sat	-0.6212	0.0551	0.0000	**0.0000**
Mon - Sun	-0.8674	0.0588	0.0000	**0.0000**
Tue - Sun	-0.9514	0.0661	0.0000	**0.0000**
Wed - Sun	-0.9475	0.0642	0.0000	**0.0000**
Thu - Sun	-0.9474	0.0611	0.0000	**0.0000**
Fri - Sun	-0.6136	0.0540	0.0000	**0.0000**
Tue - Mon	-0.0840	0.0723	0.9060	1.0000
Wed - Mon	-0.0800	0.0711	0.9183	1.0000
Thu - Mon	-0.0799	0.0692	0.9081	1.0000
Fri - Mon	0.2538	0.0638	0.0013	**0.0040**
Wed - Tue	0.0040	0.0767	1.0000	1.0000
Thu - Tue	0.0041	0.0753	1.0000	1.0000
Fri - Tue	0.3379	0.0706	0.0000	**0.0001**
Thu - Wed	0.0001	0.0733	1.0000	1.0000
Fri - Wed	0.3339	0.0682	0.0000	**0.0001**
Fri - Thu	0.3338	0.0656	0.0000	**0.0000**

Variable(s)	Parameter Estimate	Standard Error	Raw p-value	Adjusted p-value
2014 - 2013	0.0865	0.2306	0.9999	1.0000
2015 - 2013	0.1668	0.2279	0.9953	1.0000
2016 - 2013	-0.0008	0.2292	1.0000	1.0000
2017 - 2013	0.0874	0.2310	0.9999	1.0000
2018 - 2013	0.1619	0.2302	0.9964	1.0000
2019 - 2013	0.3128	0.2300	0.8604	1.0000
2020 - 2013	0.4316	0.2326	0.5526	0.8750
2015 - 2014	0.0803	0.0841	0.9774	1.0000
2016 - 2014	-0.0873	0.0873	0.9707	1.0000
2017 - 2014	0.0009	0.0923	1.0000	1.0000
2018 - 2014	0.0754	0.0916	0.9905	1.0000
2019 - 2014	0.2263	0.0918	0.1900	0.3611
2020 - 2014	0.3451	0.0996	0.0104	**0.0281**
2016 - 2015	-0.1676	0.0623	0.1112	0.2348
2017 - 2015	-0.0795	0.0673	0.9290	1.0000
2018 - 2015	-0.0049	0.0664	1.0000	1.0000
2019 - 2015	0.1459	0.0670	0.3384	0.5765
2020 - 2015	0.2647	0.0786	0.0148	**0.0388**
2017 - 2016	0.0881	0.0696	0.8998	1.0000
2018 - 2016	0.1627	0.0687	0.2334	0.4326
2019 - 2016	0.3135	0.0697	0.0001	**0.0005**
2020 - 2016	0.4323	0.0808	0.0000	**0.0000**
2018 - 2017	0.0746	0.0713	0.9625	1.0000
2019 - 2017	0.2254	0.0721	0.0321	0.0763
2020 - 2017	0.3442	0.0825	0.0007	**0.0021**
2019 - 2018	0.1509	0.0695	0.3414	0.5765
2020 - 2018	0.2697	0.0805	0.0155	**0.0394**
2020 - 2019	0.1188	0.0794	0.7897	1.0000

Table 15: P-values for onshore fishing from Wald test. Bold typeface indicates $p<0.05$; adjusted p-values have had the Benjamini and Hochberg 'false discovery rate' correction applied to them.

Variable(s)	Parameter Estimate	Standard Error	Raw p-value	Adjusted p-value
(Intercept)	-0.3577	0.3170	0.2591	0.4585
access	0.3398	0.1064	0.0014	**0.0041**
popden	0.1583	0.1009	0.1166	0.2394
precipitationyes	-0.4674	0.0898	0.0000	**0.0000**
tide standard	0.1030	0.0574	0.0729	0.1582
lat	-0.0001	0.1471	0.9995	1.0000
lat^2	-0.0312	0.1240	0.8014	1.0000
adjacent to parkTRUE	0.6765	0.2453	0.0058	**0.0164**
winterness	-0.1700	0.0240	0.0000	**0.0000**
springness	-0.0155	0.0241	0.5192	0.8395
tod	-0.0910	0.0196	0.0000	**0.0000**
tod^2	-0.0695	0.0164	0.0000	**0.0001**
duration	0.1416	0.0216	0.0000	**0.0000**
length miles	0.2672	0.1237	0.0308	0.0756
zi-(Intercept)	-2.1930	0.1894	0.0000	**0.0000**

Table 17: P-values for offshorerec from glht Tukey test. Bold typeface indicates p<0.05; adjusted p-values have had the Benjamini and Hochberg ‘false discovery rate’ correction applied to them.

Variable(s)	Parameter Estimate	Standard Error	Raw p-value	Adjusted p-value
NoTake - NonMPA	0.3312	0.4270	0.7178	1.0000
SomeTake - NonMPA	0.2309	0.4561	0.8681	1.0000
SomeTake - NoTake	-0.1003	0.4621	0.9743	1.0000
Rocky - Sandy	-1.7751	0.4025	0.0002	**0.0004**
Sand and Rocky - Sandy	-0.4725	0.2246	0.0844	0.1457
Sand and Rocky - Rocky	1.3026	0.3849	0.0018	**0.0033**
cloudy - clear	-0.2838	0.0254	0.0000	**0.0000**
partly cloudy - clear	-0.1182	0.0214	0.0000	**0.0000**
partly cloudy - cloudy	0.1656	0.0247	0.0000	**0.0000**
limited - perfect	0.0680	0.0249	0.0143	**0.0259**
shore only - perfect	0.0114	0.0840	0.9890	1.0000
shore only - limited	-0.0566	0.0850	0.7683	1.0000
Sun - Sat	-0.0491	0.0301	0.6618	0.9490
Mon - Sat	-0.4903	0.0330	0.0000	**0.0000**
Tue - Sat	-0.4540	0.0335	0.0000	**0.0000**
Wed - Sat	-0.5517	0.0332	0.0000	**0.0000**
Thu - Sat	-0.4702	0.0328	0.0000	**0.0000**
Fri - Sat	-0.3239	0.0314	0.0000	**0.0000**
Mon - Sun	-0.4412	0.0334	0.0000	**0.0000**
Tue - Sun	-0.4048	0.0342	0.0000	**0.0000**
Wed - Sun	-0.5026	0.0339	0.0000	**0.0000**
Thu - Sun	-0.4211	0.0333	0.0000	**0.0000**
Fri - Sun	-0.2748	0.0321	0.0000	**0.0000**
Tue - Mon	0.0364	0.0357	0.9495	1.0000
Wed - Mon	-0.0614	0.0354	0.5919	0.8654
Thu - Mon	0.0201	0.0351	0.9975	1.0000
Fri - Mon	0.1664	0.0340	0.0000	**0.0001**
Wed - Tue	-0.0977	0.0359	0.0933	0.1575
Thu - Tue	-0.0163	0.0357	0.9993	1.0000
Fri - Tue	0.1301	0.0347	0.0033	**0.0061**
Thu - Wed	0.0814	0.0352	0.2375	0.3760
Fri - Wed	0.2278	0.0344	0.0000	**0.0000**
Fri - Thu	0.1463	0.0338	0.0003	**0.0007**

Variable(s)	Parameter Estimate	Standard Error	Raw p-value	Adjusted p-value
2014 - 2013	0.9095	0.1545	0.0000	**0.0000**
2015 - 2013	0.8294	0.1535	0.0000	**0.0000**
2016 - 2013	0.8673	0.1544	0.0000	**0.0000**
2017 - 2013	0.9098	0.1575	0.0000	**0.0000**
2018 - 2013	0.8359	0.1553	0.0000	**0.0000**
2019 - 2013	0.8248	0.1565	0.0000	**0.0000**
2020 - 2013	1.3661	0.1583	0.0000	**0.0000**
2015 - 2014	-0.0802	0.0776	0.9667	1.0000
2016 - 2014	-0.0422	0.0791	0.9994	1.0000
2017 - 2014	0.0003	0.0849	1.0000	1.0000
2018 - 2014	-0.0737	0.0820	0.9848	1.0000
2019 - 2014	-0.0847	0.0841	0.9711	1.0000
2020 - 2014	0.4566	0.0874	0.0000	**0.0000**
2016 - 2015	0.0379	0.0639	0.9988	1.0000
2017 - 2015	0.0804	0.0695	0.9388	1.0000
2018 - 2015	0.0065	0.0675	1.0000	1.0000
2019 - 2015	-0.0045	0.0705	1.0000	1.0000
2020 - 2015	0.5368	0.0739	0.0000	**0.0000**
2017 - 2016	0.0425	0.0693	0.9986	1.0000
2018 - 2016	-0.0314	0.0675	0.9998	1.0000
2019 - 2016	-0.0424	0.0706	0.9987	1.0000
2020 - 2016	0.4988	0.0746	0.0000	**0.0000**
2018 - 2017	-0.0739	0.0720	0.9677	1.0000
2019 - 2017	-0.0849	0.0748	0.9444	1.0000
2020 - 2017	0.4563	0.0779	0.0000	**0.0000**
2019 - 2018	-0.0110	0.0718	1.0000	1.0000
2020 - 2018	0.5303	0.0760	0.0000	**0.0000**
2020 - 2019	0.5413	0.0765	0.0000	**0.0000**

Table 20: P-values for offshorerec from Wald test. Bold typeface indicates p<0.05; adjusted p-values have had the Benjamini and Hochberg ‘false discovery rate’ correction applied to them.

Variable(s)	Parameter Estimate	Standard Error	Raw p-value	Adjusted p-value
(Intercept)	0.5042	0.4541	0.2668	0.4138
access	0.3498	0.1041	0.0008	**0.0015**
popden	0.2795	0.1318	0.0340	0.0601
precipitationyes	-0.3231	0.0558	0.0000	**0.0000**
tide standard	0.0405	0.0326	0.2133	0.3525
lat	-1.1001	0.2671	0.0000	**0.0001**
lat^2	0.1709	0.1913	0.3715	0.5647
adjacent to parkTRUE	0.4265	0.3508	0.2240	0.3622
winterness	-0.4581	0.0137	0.0000	**0.0000**
springness	-0.3763	0.0144	0.0000	**0.0000**
tod	0.2129	0.0103	0.0000	**0.0000**
tod^2	-0.2102	0.0087	0.0000	**0.0000**
duration	0.1814	0.0189	0.0000	**0.0000**
length miles	0.0843	0.1574	0.5921	0.8654
zi-(Intercept)	-3.4575	0.1530	0.0000	**0.0000**

Table 22: P-values for animals from glht Tukey test. Bold typeface indicates p<0.05; adjusted p-values have had the Benjamini and Hochberg 'false discovery rate' correction applied to them.

Variable(s)	Parameter Estimate	Standard Error	Raw p-value	Adjusted p-value
NoTake - NonMPA	-1.3462	0.3981	0.0021	**0.0052**
SomeTake - NonMPA	-1.7119	0.4322	0.0002	**0.0008**
SomeTake - NoTake	-0.3657	0.4415	0.6850	0.8790
Rocky - Sandy	-0.8358	0.3748	0.0623	0.1163
Sand and Rocky - Sandy	0.5030	0.2003	0.0297	0.0624
Sand and Rocky - Rocky	1.3388	0.3730	0.0011	**0.0029**
cloudy - clear	-0.1435	0.0271	0.0000	**0.0000**
partly cloudy - clear	-0.0540	0.0236	0.0569	0.1112
partly cloudy - cloudy	0.0895	0.0258	0.0014	**0.0038**
limited - perfect	-0.1008	0.0286	0.0009	**0.0025**
shore only - perfect	0.0629	0.0762	0.6711	0.8790
shore only - limited	0.1637	0.0781	0.0816	0.1459
Sun - Sat	0.0953	0.0326	0.0535	0.1070
Mon - Sat	-0.4399	0.0360	0.0000	**0.0000**
Tue - Sat	-0.6037	0.0386	0.0000	**0.0000**
Wed - Sat	-0.3964	0.0366	0.0000	**0.0000**
Thu - Sat	-0.4873	0.0359	0.0000	**0.0000**
Fri - Sat	-0.3703	0.0346	0.0000	**0.0000**
Mon - Sun	-0.5351	0.0360	0.0000	**0.0000**
Tue - Sun	-0.6990	0.0387	0.0000	**0.0000**
Wed - Sun	-0.4917	0.0369	0.0000	**0.0000**
Thu - Sun	-0.5825	0.0360	0.0000	**0.0000**
Fri - Sun	-0.4655	0.0349	0.0000	**0.0000**
Tue - Mon	-0.1638	0.0400	0.0009	**0.0025**
Wed - Mon	0.0434	0.0383	0.9176	1.0000
Thu - Mon	-0.0474	0.0379	0.8738	0.9961
Fri - Mon	0.0696	0.0371	0.4938	0.7031
Wed - Tue	0.2073	0.0406	0.0000	**0.0000**
Thu - Tue	0.1165	0.0404	0.0595	0.1135
Fri - Tue	0.2334	0.0396	0.0000	**0.0000**
Thu - Wed	-0.0908	0.0383	0.2095	0.3259
Fri - Wed	0.0262	0.0375	0.9927	1.0000
Fri - Thu	0.1170	0.0368	0.0247	0.0532

Variable(s)	Parameter Estimate	Standard Error	Raw p-value	Adjusted p-value
2014 - 2013	0.0213	0.1363	1.0000	1.0000
2015 - 2013	-0.0862	0.1342	0.9980	1.0000
2016 - 2013	-0.2748	0.1355	0.4402	0.6488
2017 - 2013	-0.1828	0.1374	0.8775	0.9961
2018 - 2013	-0.1664	0.1369	0.9203	1.0000
2019 - 2013	-0.2430	0.1379	0.6246	0.8458
2020 - 2013	0.2058	0.1404	0.8116	0.9544
2015 - 2014	-0.1076	0.0647	0.6915	0.8790
2016 - 2014	-0.2961	0.0670	0.0002	**0.0007**
2017 - 2014	-0.2041	0.0713	0.0729	0.1331
2018 - 2014	-0.1878	0.0709	0.1270	0.2092
2019 - 2014	-0.2644	0.0729	0.0061	**0.0138**
2020 - 2014	0.1845	0.0785	0.2484	0.3725
2016 - 2015	-0.1885	0.0510	0.0048	**0.0112**
2017 - 2015	-0.0965	0.0552	0.6343	0.8458
2018 - 2015	-0.0802	0.0548	0.8125	0.9544
2019 - 2015	-0.1568	0.0573	0.1023	0.1754
2020 - 2015	0.2921	0.0650	0.0001	**0.0005**
2017 - 2016	0.0920	0.0561	0.7072	0.8790
2018 - 2016	0.1083	0.0564	0.5130	0.7182
2019 - 2016	0.0317	0.0587	0.9994	1.0000
2020 - 2016	0.4806	0.0663	0.0000	**0.0000**
2018 - 2017	0.0163	0.0588	1.0000	1.0000
2019 - 2017	-0.0602	0.0609	0.9734	1.0000
2020 - 2017	0.3886	0.0681	0.0000	**0.0000**
2019 - 2018	-0.0766	0.0599	0.8979	1.0000
2020 - 2018	0.3722	0.0677	0.0000	**0.0000**
2020 - 2019	0.4488	0.0672	0.0000	**0.0000**

Table 25: P-values for animals from Wald test. Bold typeface indicates p<0.05; adjusted p-values have had the Benjamini and Hochberg 'false discovery rate' correction applied to them.

Variable(s)	Parameter Estimate	Standard Error	Raw p-value	Adjusted p-value
(Intercept)	1.1917	0.7175	0.0967	0.1693
access	0.3290	0.1074	0.0022	**0.0053**
popden	0.4707	0.1351	0.0005	**0.0015**
precipitationyes	-0.4168	0.0538	0.0000	**0.0000**
tide standard	-0.3892	0.0345	0.0000	**0.0000**
lat	1.2375	0.9039	0.1710	0.2710
lat^2	-2.9628	2.4151	0.2199	0.3358
lat^3	-2.9372	2.0392	0.1498	0.2419
lat^4	4.1331	2.6083	0.1131	0.1899
lat^5	1.0297	1.7852	0.5641	0.7768
lat^6	-1.4208	0.7013	0.0428	0.0876
lat^7	0.1379	0.5994	0.8180	0.9544
lat^8	0.0407	0.1099	0.7116	0.8790
winterness	-0.0563	0.0143	0.0001	**0.0003**
springness	-0.0111	0.0149	0.4566	0.6613
tod	0.1846	0.0112	0.0000	**0.0000**
tod^2	-0.0587	0.0093	0.0000	**0.0000**
duration	0.4163	0.0291	0.0000	**0.0000**
$duration^2$	-0.0269	0.0049	0.0000	**0.0000**
length miles	0.4203	0.1863	0.0241	0.0532
length $miles^2$	-0.2471	0.0804	0.0021	**0.0052**
adjacent to parkTRUE	-0.0775	0.3315	0.8151	0.9544
zi-(Intercept)	-16.0618	535.6621	0.9761	1.0000

Table 27: P-values for recboating from glht Tukey test. Bold typeface indicates p<0.05; adjusted p-values have had the Benjamini and Hochberg 'false discovery rate' correction applied to them.

Variable(s)	Parameter Estimate	Standard Error	Raw p-value	Adjusted p-value
NoTake - NonMPA	-0.3365	0.3700	0.6339	0.8451
SomeTake - NonMPA	-0.0441	0.4122	0.9937	1.0000
SomeTake - NoTake	0.2924	0.4071	0.7524	0.9554
cloudy - clear	-0.2096	0.0378	0.0000	**0.0000**
partly cloudy - clear	-0.1072	0.0315	0.0020	**0.0045**
partly cloudy - cloudy	0.1023	0.0363	0.0132	**0.0258**
limited - perfect	-0.1170	0.0408	0.0094	**0.0193**
shore only - perfect	-0.3267	0.1700	0.1157	0.2012
shore only - limited	-0.2098	0.1716	0.4113	0.5986
Sun - Sat	-0.0333	0.0407	0.9826	1.0000
Mon - Sat	-0.7137	0.0546	0.0000	**0.0000**
Tue - Sat	-0.7258	0.0556	0.0000	**0.0000**
Wed - Sat	-0.8504	0.0517	0.0000	**0.0000**
Thu - Sat	-0.6968	0.0503	0.0000	**0.0000**
Fri - Sat	-0.4911	0.0420	0.0000	**0.0000**
Mon - Sun	-0.6804	0.0550	0.0000	**0.0000**
Tue - Sun	-0.6925	0.0563	0.0000	**0.0000**
Wed - Sun	-0.8171	0.0524	0.0000	**0.0000**
Thu - Sun	-0.6635	0.0516	0.0000	**0.0000**
Fri - Sun	-0.4579	0.0442	0.0000	**0.0000**
Tue - Mon	-0.0121	0.0652	1.0000	1.0000
Wed - Mon	-0.1367	0.0620	0.2869	0.4414
Thu - Mon	0.0169	0.0616	1.0000	1.0000
Fri - Mon	0.2226	0.0560	0.0013	**0.0033**
Wed - Tue	-0.1246	0.0625	0.4115	0.5986
Thu - Tue	0.0290	0.0621	0.9992	1.0000
Fri - Tue	0.2347	0.0570	0.0007	**0.0020**
Thu - Wed	0.1536	0.0588	0.1190	0.2025
Fri - Wed	0.3592	0.0532	0.0000	**0.0000**
Fri - Thu	0.2057	0.0521	0.0014	**0.0034**

Variable(s)	Parameter Estimate	Standard Error	Raw p-value	Adjusted p-value
2014 - 2013	-0.5645	0.1087	0.0000	**0.0000**
2015 - 2013	-0.9331	0.1098	0.0000	**0.0000**
2016 - 2013	-1.0372	0.1151	0.0000	**0.0000**
2017 - 2013	-1.0467	0.1247	0.0000	**0.0000**
2018 - 2013	-1.2536	0.1297	0.0000	**0.0000**
2019 - 2013	-0.9895	0.1179	0.0000	**0.0000**
2020 - 2013	-0.9129	0.1312	0.0000	**0.0000**
2015 - 2014	-0.3686	0.0910	0.0013	**0.0033**
2016 - 2014	-0.4727	0.0968	0.0000	**0.0001**
2017 - 2014	-0.4822	0.1109	0.0004	**0.0011**
2018 - 2014	-0.6891	0.1165	0.0000	**0.0000**
2019 - 2014	-0.4251	0.1050	0.0014	**0.0034**
2020 - 2014	-0.3484	0.1215	0.0772	0.1404
2016 - 2015	-0.1041	0.0924	0.9499	1.0000
2017 - 2015	-0.1136	0.1069	0.9633	1.0000
2018 - 2015	-0.3205	0.1132	0.0855	0.1519
2019 - 2015	-0.0565	0.1028	0.9994	1.0000
2020 - 2015	0.0202	0.1195	1.0000	1.0000
2017 - 2016	-0.0095	0.1099	1.0000	1.0000
2018 - 2016	-0.2164	0.1169	0.5791	0.7852
2019 - 2016	0.0476	0.1076	0.9998	1.0000
2020 - 2016	0.1243	0.1236	0.9730	1.0000
2018 - 2017	-0.2069	0.1266	0.7250	0.9412
2019 - 2017	0.0571	0.1164	0.9997	1.0000
2020 - 2017	0.1338	0.1300	0.9694	1.0000
2019 - 2018	0.2640	0.1230	0.3800	0.5735
2020 - 2018	0.3407	0.1366	0.1938	0.3100
2020 - 2019	0.0767	0.1247	0.9987	1.0000

Table 30: P-values for recboating from Wald test. Bold typeface indicates p<0.05; adjusted p-values have had the Benjamini and Hochberg 'false discovery rate' correction applied to them.

Variable(s)	Parameter Estimate	Standard Error	Raw p-value	Adjusted p-value
(Intercept)	1.9083	0.8910	0.0322	0.0613
access	0.1192	0.1111	0.2832	0.4414
popden	0.3745	0.1409	0.0079	**0.0166**
precipitationyes	-0.4371	0.0990	0.0000	**0.0000**
tide standard	0.0965	0.0515	0.0609	0.1132
lat	-1.2360	0.9148	0.1767	0.2884
lat^2	-7.2003	2.3392	0.0021	**0.0046**
lat^3	-0.1426	2.1002	0.9459	1.0000
lat^4	7.7727	2.5835	0.0026	**0.0057**
lat^5	-1.0881	1.8558	0.5577	0.7692
lat^6	-2.3544	0.7007	0.0008	**0.0021**
lat^7	0.9299	0.6223	0.1351	0.2251
lat^8	-0.0933	0.1159	0.4208	0.6011
winterness	-0.3176	0.0200	0.0000	**0.0000**
springness	-0.2298	0.0214	0.0000	**0.0000**
adjacent to parkTRUE	-0.1151	0.3326	0.7294	0.9412
tod	-0.0455	0.0181	0.0120	**0.0239**
tod^2	-0.3356	0.0168	0.0000	**0.0000**
duration	0.3559	0.0206	0.0000	**0.0000**
length miles	0.0214	0.1350	0.8740	1.0000
survey site typeLand	-0.2952	0.5019	0.5564	0.7692
zi-(Intercept)	-2.0010	0.1307	0.0000	**0.0000**

Table 32: P-values for offshorefishing from glht Tukey test. Bold typeface indicates p<0.05; adjusted p-values have had the Benjamini and Hochberg ‘false discovery rate’ correction applied to them.

Variable(s)	Parameter Estimate	Standard Error	Raw p-value	Adjusted p-value
NoTake - NonMPA	-0.5647	0.4278	0.3832	0.8477
SomeTake - NonMPA	0.4794	0.4612	0.5512	1.0000
SomeTake - NoTake	1.0440	0.4870	0.0808	0.2106
cloudy - clear	0.1952	0.0609	0.0038	**0.0138**
partly cloudy - clear	-0.1381	0.0551	0.0325	0.0990
partly cloudy - cloudy	-0.3333	0.0585	0.0000	**0.0000**
limited - perfect	-0.3052	0.0666	0.0000	**0.0000**
shore only - perfect	-1.0359	0.2048	0.0000	**0.0000**
shore only - limited	-0.7306	0.2099	0.0011	**0.0043**
Sun - Sat	0.0933	0.0733	0.8624	1.0000
Mon - Sat	-0.5077	0.0881	0.0000	**0.0000**
Tue - Sat	-0.5869	0.0924	0.0000	**0.0000**
Wed - Sat	-0.3890	0.0848	0.0001	**0.0004**
Thu - Sat	-0.4645	0.0825	0.0000	**0.0000**
Fri - Sat	-0.3258	0.0721	0.0001	**0.0005**
Mon - Sun	-0.6011	0.0892	0.0000	**0.0000**
Tue - Sun	-0.6803	0.0942	0.0000	**0.0000**
Wed - Sun	-0.4824	0.0870	0.0000	**0.0000**
Thu - Sun	-0.5579	0.0855	0.0000	**0.0000**
Fri - Sun	-0.4191	0.0770	0.0000	**0.0000**
Tue - Mon	-0.0792	0.1007	0.9861	1.0000
Wed - Mon	0.1187	0.0947	0.8712	1.0000
Thu - Mon	0.0432	0.0952	0.9993	1.0000
Fri - Mon	0.1819	0.0887	0.3787	0.8477
Wed - Tue	0.1979	0.0991	0.4129	0.8866
Thu - Tue	0.1224	0.0992	0.8792	1.0000
Fri - Tue	0.2612	0.0935	0.0753	0.2035
Thu - Wed	-0.0755	0.0918	0.9824	1.0000
Fri - Wed	0.0632	0.0859	0.9902	1.0000
Fri - Thu	0.1387	0.0831	0.6325	1.0000

Variable(s)	Parameter Estimate	Standard Error	Raw p-value	Adjusted p-value
2014 - 2013	0.1322	0.1947	0.9974	1.0000
2015 - 2013	-0.0691	0.1879	1.0000	1.0000
2016 - 2013	-0.0138	0.1908	1.0000	1.0000
2017 - 2013	-0.0906	0.1943	0.9998	1.0000
2018 - 2013	-0.2670	0.1968	0.8704	1.0000
2019 - 2013	-0.1143	0.1924	0.9989	1.0000
2020 - 2013	0.0432	0.2034	1.0000	1.0000
2015 - 2014	-0.2013	0.1176	0.6682	1.0000
2016 - 2014	-0.1460	0.1203	0.9241	1.0000
2017 - 2014	-0.2228	0.1306	0.6720	1.0000
2018 - 2014	-0.3992	0.1342	0.0555	0.1621
2019 - 2014	-0.2465	0.1316	0.5569	1.0000
2020 - 2014	-0.0890	0.1484	0.9988	1.0000
2016 - 2015	0.0552	0.1003	0.9993	1.0000
2017 - 2015	-0.0215	0.1121	1.0000	1.0000
2018 - 2015	-0.1979	0.1165	0.6766	1.0000
2019 - 2015	-0.0452	0.1143	0.9999	1.0000
2020 - 2015	0.1123	0.1349	0.9907	1.0000
2017 - 2016	-0.0767	0.1127	0.9973	1.0000
2018 - 2016	-0.2531	0.1183	0.3760	0.8477
2019 - 2016	-0.1004	0.1158	0.9881	1.0000
2020 - 2016	0.0571	0.1363	0.9999	1.0000
2018 - 2017	-0.1764	0.1237	0.8380	1.0000
2019 - 2017	-0.0237	0.1211	1.0000	1.0000
2020 - 2017	0.1338	0.1403	0.9793	1.0000
2019 - 2018	0.1527	0.1252	0.9223	1.0000
2020 - 2018	0.3102	0.1441	0.3686	0.8477
2020 - 2019	0.1575	0.1381	0.9448	1.0000

Table 35: P-values for offshorefishing from Wald test. Bold typeface indicates p<0.05; adjusted p-values have had the Benjamini and Hochberg ‘false discovery rate’ correction applied to them.

Variable(s)	Parameter Estimate	Standard Error	Raw p-value	Adjusted p-value
(Intercept)	-1.6273	0.3093	0.0000	**0.0000**
access	-0.0676	0.0967	0.4847	0.9829
popden	-0.0156	0.1186	0.8957	1.0000
precipitationyes	-0.0223	0.1159	0.8476	1.0000
lat	-0.4081	0.1787	0.0224	0.0709
lat^2	0.1118	0.1565	0.4750	0.9829
adjacent to parkTRUE	-0.8176	0.3024	0.0069	**0.0238**
winterness	-0.0319	0.0326	0.3265	0.8219
springness	-0.3664	0.0331	0.0000	**0.0000**
tod	-0.3963	0.0312	0.0000	**0.0000**
tod^2	-0.2568	0.0288	0.0000	**0.0000**
duration	0.3706	0.0378	0.0000	**0.0000**
length miles	0.2108	0.1175	0.0729	0.2035
survey site typeBoat	1.0741	0.4191	0.0104	**0.0345**
zi-(Intercept)	-17.9151	730.3133	0.9804	1.0000

Table 37: P-values for onshorerec from glht Tukey test. Bold typeface indicates p<0.05; adjusted p-values have had the Benjamini and Hochberg 'false discovery rate' correction applied to them.

Variable(s)	Parameter Estimate	Standard Error	Raw p-value	Adjusted p-value
NoTake - NonMPA	-0.3760	0.3065	0.4369	0.5645
SomeTake - NonMPA	-0.5298	0.3243	0.2311	0.3444
SomeTake - NoTake	-0.1539	0.3354	0.8903	0.9530
Rocky - Sandy	-1.1577	0.2905	0.0002	**0.0005**
Sand and Rocky - Sandy	-0.2875	0.1790	0.2361	0.3451
Sand and Rocky - Rocky	0.8702	0.2767	0.0044	**0.0094**
cloudy - clear	-0.3132	0.0145	0.0000	**0.0000**
partly cloudy - clear	-0.0991	0.0123	0.0000	**0.0000**
partly cloudy - cloudy	0.2141	0.0138	0.0000	**0.0000**
limited - perfect	0.0986	0.0142	0.0000	**0.0000**
shore only - perfect	0.0278	0.0449	0.7968	0.8797
shore only - limited	-0.0708	0.0456	0.2451	0.3515
Sun - Sat	-0.0235	0.0167	0.7987	0.8797
Mon - Sat	-0.4811	0.0189	0.0000	**0.0000**
Tue - Sat	-0.5455	0.0198	0.0000	**0.0000**
Wed - Sat	-0.5122	0.0189	0.0000	**0.0000**
Thu - Sat	-0.5486	0.0188	0.0000	**0.0000**
Fri - Sat	-0.4453	0.0185	0.0000	**0.0000**
Mon - Sun	-0.4576	0.0190	0.0000	**0.0000**
Tue - Sun	-0.5220	0.0199	0.0000	**0.0000**
Wed - Sun	-0.4887	0.0191	0.0000	**0.0000**
Thu - Sun	-0.5251	0.0189	0.0000	**0.0000**
Fri - Sun	-0.4218	0.0186	0.0000	**0.0000**
Tue - Mon	-0.0644	0.0208	0.0325	0.0549
Wed - Mon	-0.0311	0.0201	0.7158	0.8243
Thu - Mon	-0.0675	0.0201	0.0140	**0.0268**
Fri - Mon	0.0358	0.0199	0.5485	0.6834
Wed - Tue	0.0333	0.0209	0.6878	0.8141
Thu - Tue	-0.0031	0.0210	1.0000	1.0000
Fri - Tue	0.1002	0.0209	0.0000	**0.0001**
Thu - Wed	-0.0364	0.0201	0.5415	0.6834
Fri - Wed	0.0669	0.0200	0.0141	**0.0268**
Fri - Thu	0.1033	0.0199	0.0000	**0.0000**

Variable(s)	Parameter Estimate	Standard Error	Raw p-value	Adjusted p-value
2014 - 2013	0.2990	0.0914	0.0211	**0.0372**
2015 - 2013	0.3102	0.0903	0.0118	**0.0236**
2016 - 2013	0.1682	0.0910	0.5617	0.6886
2017 - 2013	0.2431	0.0920	0.1269	0.2009
2018 - 2013	0.2041	0.0918	0.3151	0.4276
2019 - 2013	0.1442	0.0924	0.7550	0.8564
2020 - 2013	0.5399	0.0938	0.0000	**0.0000**
2015 - 2014	0.0113	0.0387	1.0000	1.0000
2016 - 2014	-0.1308	0.0397	0.0195	**0.0353**
2017 - 2014	-0.0559	0.0421	0.8769	0.9520
2018 - 2014	-0.0949	0.0423	0.3027	0.4183
2019 - 2014	-0.1548	0.0440	0.0088	**0.0180**
2020 - 2014	0.2409	0.0470	0.0000	**0.0000**
2016 - 2015	-0.1420	0.0298	0.0000	**0.0001**
2017 - 2015	-0.0671	0.0317	0.3761	0.5014
2018 - 2015	-0.1062	0.0320	0.0181	**0.0335**
2019 - 2015	-0.1661	0.0345	0.0000	**0.0001**
2020 - 2015	0.2296	0.0383	0.0000	**0.0000**
2017 - 2016	0.0749	0.0324	0.2642	0.3718
2018 - 2016	0.0358	0.0327	0.9524	0.9916
2019 - 2016	-0.0240	0.0356	0.9973	1.0000
2020 - 2016	0.3716	0.0394	0.0000	**0.0000**
2018 - 2017	-0.0390	0.0338	0.9376	0.9897
2019 - 2017	-0.0989	0.0362	0.1010	0.1633
2020 - 2017	0.2968	0.0400	0.0000	**0.0000**
2019 - 2018	-0.0599	0.0362	0.6963	0.8141
2020 - 2018	0.3358	0.0401	0.0000	**0.0000**
2020 - 2019	0.3957	0.0408	0.0000	**0.0000**

Table 40: P-values for onshorerec from Wald test. Bold typeface indicates p<0.05; adjusted p-values have had the Benjamini and Hochberg 'false discovery rate' correction applied to them.

Variable(s)	Parameter Estimate	Standard Error	Raw p-value	Adjusted p-value
(Intercept)	3.4091	0.3137	0.0000	**0.0000**
access	0.3145	0.0755	0.0000	**0.0001**
popden	0.1965	0.0969	0.0427	0.0706
precipitationyes	-0.3513	0.0313	0.0000	**0.0000**
tide standard	-0.1275	0.0186	0.0000	**0.0000**
lat	-0.4646	0.2041	0.0228	**0.0394**
lat^2	0.1716	0.1358	0.2064	0.3138
winterness	-0.4210	0.0077	0.0000	**0.0000**
springness	-0.1389	0.0081	0.0000	**0.0000**
tod	0.4329	0.0064	0.0000	**0.0000**
tod^2	-0.2693	0.0052	0.0000	**0.0000**
duration	0.1570	0.0092	0.0000	**0.0000**
length miles	0.0856	0.1105	0.4382	0.5645
adjacent to parkTRUE	0.3610	0.2503	0.1493	0.2315
has tidepoolingYes	0.0964	0.1906	0.6130	0.7395

Table 42: P-values for hand collection from glht Tukey test. Bold typeface indicates p<0.05; adjusted p-values have had the Benjamini and Hochberg 'false discovery rate' correction applied to them.

Variable(s)	Parameter Estimate	Standard Error	Raw p-value	Adjusted p-value
NoTake - NonMPA	1.3945	1.1727	0.4515	0.8707
SomeTake - NonMPA	0.4821	1.2279	0.9165	1.0000
SomeTake - NoTake	-0.9125	0.7578	0.4426	0.8707
Rocky - Sandy	-1.0356	0.5837	0.1706	0.4187
Sand and Rocky - Sandy	-0.2683	0.3249	0.6785	1.0000
Sand and Rocky - Rocky	0.7672	0.5854	0.3785	0.8068
cloudy - clear	0.3078	0.1043	0.0088	**0.0377**
partly cloudy - clear	0.1347	0.0946	0.3278	0.7176
partly cloudy - cloudy	-0.1731	0.0961	0.1687	0.4187
limited - perfect	-0.2214	0.1049	0.0776	0.2329
shore only - perfect	-0.2917	0.3031	0.5810	1.0000
shore only - limited	-0.0703	0.3053	0.9691	1.0000
Sun - Sat	-0.2200	0.1171	0.4909	0.9016
Mon - Sat	-0.7633	0.1452	0.0000	**0.0000**
Tue - Sat	-0.6655	0.1396	0.0000	**0.0004**
Wed - Sat	-0.7600	0.1395	0.0000	**0.0000**
Thu - Sat	-0.7971	0.1412	0.0000	**0.0000**
Fri - Sat	-0.5771	0.1312	0.0002	**0.0016**
Mon - Sun	-0.5433	0.1447	0.0033	**0.0178**
Tue - Sun	-0.4456	0.1386	0.0219	0.0788
Wed - Sun	-0.5400	0.1399	0.0022	**0.0138**
Thu - Sun	-0.5771	0.1416	0.0009	**0.0059**
Fri - Sun	-0.3571	0.1339	0.1052	0.3042
Tue - Mon	0.0978	0.1585	0.9962	1.0000
Wed - Mon	0.0033	0.1597	1.0000	1.0000
Thu - Mon	-0.0338	0.1614	1.0000	1.0000
Fri - Mon	0.1862	0.1557	0.8947	1.0000
Wed - Tue	-0.0944	0.1549	0.9965	1.0000
Thu - Tue	-0.1315	0.1571	0.9808	1.0000
Fri - Tue	0.0885	0.1500	0.9971	1.0000
Thu - Wed	-0.0371	0.1567	1.0000	1.0000
Fri - Wed	0.1829	0.1510	0.8888	1.0000
Fri - Thu	0.2200	0.1521	0.7739	1.0000

Variable(s)	Parameter Estimate	Standard Error	Raw p-value	Adjusted p-value
2014 - 2013	1.4688	1.0569	0.8437	1.0000
2015 - 2013	1.1287	1.0561	0.9569	1.0000
2016 - 2013	0.9917	1.0572	0.9790	1.0000
2017 - 2013	1.0279	1.0650	0.9754	1.0000
2018 - 2013	0.8306	1.0676	0.9931	1.0000
2019 - 2013	1.5271	1.0620	0.8190	1.0000
2020 - 2013	1.9651	1.0673	0.5586	0.9836
2015 - 2014	-0.3401	0.2843	0.9228	1.0000
2016 - 2014	-0.4771	0.2890	0.6902	1.0000
2017 - 2014	-0.4409	0.3159	0.8408	1.0000
2018 - 2014	-0.6383	0.3286	0.4871	0.9016
2019 - 2014	0.0583	0.3095	1.0000	1.0000
2020 - 2014	0.4962	0.3336	0.7917	1.0000
2016 - 2015	-0.1370	0.2120	0.9978	1.0000
2017 - 2015	-0.1008	0.2462	0.9999	1.0000
2018 - 2015	-0.2981	0.2600	0.9376	1.0000
2019 - 2015	0.3984	0.2382	0.6756	1.0000
2020 - 2015	0.8364	0.2697	0.0345	0.1163
2017 - 2016	0.0362	0.2322	1.0000	1.0000
2018 - 2016	-0.1612	0.2498	0.9978	1.0000
2019 - 2016	0.5354	0.2299	0.2501	0.5788
2020 - 2016	0.9733	0.2605	0.0039	**0.0198**
2018 - 2017	-0.1974	0.2774	0.9960	1.0000
2019 - 2017	0.4992	0.2596	0.5009	0.9016
2020 - 2017	0.9371	0.2861	0.0197	0.0759
2019 - 2018	0.6965	0.2638	0.1240	0.3450
2020 - 2018	1.1345	0.2965	0.0026	**0.0150**
2020 - 2019	0.4380	0.2661	0.6934	1.0000

Table 45: P-values for hand collection from Wald test. Bold typeface indicates p<0.05; adjusted p-values have had the Benjamini and Hochberg 'false discovery rate' correction applied to them.

Variable(s)	Parameter Estimate	Standard Error	Raw p-value	Adjusted p-value
(Intercept)	-3.2064	1.1296	0.0045	**0.0216**
access	0.3030	0.1327	0.0224	0.0788
popden	0.2140	0.1421	0.1320	0.3450
precipitationyes	0.0315	0.1825	0.8631	1.0000
tide standard	-1.7896	0.1368	0.0000	**0.0000**
lat	-0.6923	0.2526	0.0061	**0.0276**
lat^2	0.3289	0.1782	0.0649	0.2103
adjacent to parkTRUE	0.4087	0.3383	0.2271	0.5411
winterness	-0.3428	0.0603	0.0000	**0.0000**
springness	-0.0921	0.0609	0.1301	0.3450
has tidepoolingYes	0.2624	0.3321	0.4295	0.8697
tod	0.0488	0.0438	0.2646	0.5955
tod^2	-0.1504	0.0389	0.0001	**0.0010**
duration	0.3586	0.0446	0.0000	**0.0000**
length miles	0.3435	0.1916	0.0729	0.2273
zi-(Intercept)	0.4304	0.1785	0.0159	0.0643
disp-(Intercept)	2.1168	0.1342	0.0000	**0.0000**
disp-has tidepoolingYes	-0.7193	0.1911	0.0002	**0.0014**
disp-beach typeRocky	-0.1580	0.3343	0.6364	1.0000
disp-beach typeSand and Rocky	-0.1543	0.1883	0.4125	0.8567

Table 47: P-values for total activities from glht Tukey test. Bold typeface indicates p<0.05; adjusted p-values have had the Benjamini and Hochberg 'false discovery rate' correction applied to them.

Variable(s)	Parameter Estimate	Standard Error	Raw p-value	Adjusted p-value
NoTake - NonMPA	-0.4768	0.2688	0.1781	0.2743
SomeTake - NonMPA	-0.4831	0.2915	0.2215	0.3280
SomeTake - NoTake	-0.0063	0.2911	0.9997	1.0000
Rocky - Sandy	-0.8262	0.2905	0.0119	**0.0219**
Sand and Rocky - Sandy	-0.2016	0.1800	0.4935	0.6667
Sand and Rocky - Rocky	0.6246	0.2732	0.0551	0.0903
cloudy - clear	-0.2520	0.0154	0.0000	**0.0000**
partly cloudy - clear	-0.0965	0.0133	0.0000	**0.0000**
partly cloudy - cloudy	0.1555	0.0146	0.0000	**0.0000**
limited - perfect	0.0038	0.0160	0.9670	1.0000
shore only - perfect	-0.1397	0.0450	0.0043	**0.0084**
shore only - limited	-0.1435	0.0459	0.0040	**0.0081**
Sun - Sat	0.0246	0.0190	0.8525	1.0000
Mon - Sat	-0.5453	0.0207	0.0000	**0.0000**
Tue - Sat	-0.6627	0.0218	0.0000	**0.0000**
Wed - Sat	-0.6520	0.0208	0.0000	**0.0000**
Thu - Sat	-0.6775	0.0204	0.0000	**0.0000**
Fri - Sat	-0.5347	0.0191	0.0000	**0.0000**
Mon - Sun	-0.5699	0.0208	0.0000	**0.0000**
Tue - Sun	-0.6873	0.0220	0.0000	**0.0000**
Wed - Sun	-0.6767	0.0211	0.0000	**0.0000**
Thu - Sun	-0.7022	0.0207	0.0000	**0.0000**
Fri - Sun	-0.5593	0.0197	0.0000	**0.0000**
Tue - Mon	-0.1174	0.0225	0.0000	**0.0000**
Wed - Mon	-0.1068	0.0217	0.0000	**0.0000**
Thu - Mon	-0.1323	0.0216	0.0000	**0.0000**
Fri - Mon	0.0106	0.0208	0.9987	1.0000
Wed - Tue	0.0106	0.0225	0.9992	1.0000
Thu - Tue	-0.0149	0.0225	0.9945	1.0000
Fri - Tue	0.1280	0.0218	0.0000	**0.0000**
Thu - Wed	-0.0255	0.0214	0.8976	1.0000
Fri - Wed	0.1174	0.0208	0.0000	**0.0000**
Fri - Thu	0.1429	0.0204	0.0000	**0.0000**

Variable(s)	Parameter Estimate	Standard Error	Raw p-value	Adjusted p-value
2014 - 2013	-0.0265	0.0708	0.9999	1.0000
2015 - 2013	0.0130	0.0683	1.0000	1.0000
2016 - 2013	-0.0566	0.0687	0.9911	1.0000
2017 - 2013	-0.1077	0.0702	0.7777	0.9817
2018 - 2013	-0.0971	0.0700	0.8555	1.0000
2019 - 2013	-0.1915	0.0697	0.1014	0.1627
2020 - 2013	0.2452	0.0722	0.0145	**0.0260**
2015 - 2014	0.0395	0.0397	0.9731	1.0000
2016 - 2014	-0.0301	0.0406	0.9953	1.0000
2017 - 2014	-0.0811	0.0438	0.5669	0.7399
2018 - 2014	-0.0706	0.0437	0.7281	0.9344
2019 - 2014	-0.1649	0.0441	0.0043	**0.0084**
2020 - 2014	0.2717	0.0485	0.0000	**0.0000**
2016 - 2015	-0.0696	0.0324	0.3673	0.5142
2017 - 2015	-0.1207	0.0357	0.0153	**0.0262**
2018 - 2015	-0.1101	0.0358	0.0405	0.0678
2019 - 2015	-0.2045	0.0365	0.0000	**0.0000**
2020 - 2015	0.2322	0.0420	0.0000	**0.0000**
2017 - 2016	-0.0511	0.0359	0.8389	1.0000
2018 - 2016	-0.0405	0.0364	0.9513	1.0000
2019 - 2016	-0.1349	0.0370	0.0060	**0.0112**
2020 - 2016	0.3018	0.0424	0.0000	**0.0000**
2018 - 2017	0.0106	0.0386	1.0000	1.0000
2019 - 2017	-0.0838	0.0389	0.3654	0.5142
2020 - 2017	0.3528	0.0439	0.0000	**0.0000**
2019 - 2018	-0.0944	0.0386	0.2073	0.3131
2020 - 2018	0.3423	0.0439	0.0000	**0.0000**
2020 - 2019	0.4366	0.0430	0.0000	**0.0000**

Table 50: P-values for total activities from Wald test. Bold typeface indicates p<0.05; adjusted p-values have had the Benjamini and Hochberg ‘false discovery rate’ correction applied to them.

Variable(s)	Parameter Estimate	Standard Error	Raw p-value	Adjusted p-value
(Intercept)	3.9149	0.2912	0.0000	**0.0000**
access	0.3238	0.0757	0.0000	**0.0000**
popden	0.2170	0.0892	0.0149	**0.0261**
precipitationyes	-0.4232	0.0301	0.0000	**0.0000**
tide standard	-0.1914	0.0198	0.0000	**0.0000**
lat	-0.6333	0.1902	0.0009	**0.0018**
lat^2	0.1453	0.1256	0.2475	0.3596
adjacent to parkTRUE	0.3323	0.2326	0.1531	0.2406
winterness	-0.3840	0.0081	0.0000	**0.0000**
springness	-0.1490	0.0086	0.0000	**0.0000**
has tidepoolingYes	0.1095	0.1855	0.5549	0.7367
tod	0.3937	0.0065	0.0000	**0.0000**
tod^2	-0.2332	0.0051	0.0000	**0.0000**
duration	0.3619	0.0106	0.0000	**0.0000**
length miles	-0.0738	0.0900	0.4124	0.5671
survey site typeBoat	-2.2687	0.5857	0.0001	**0.0002**
