# Supplementary material for: Demonstrating conservation impacts in California Marine Protected Areas using large-scale participatory science data
Source: PLoS One. 2026 Jun 25;21(6):e0352449. doi: 10.1371/journal.pone.0352449 (PMC13298793; doi:10.1371/journal.pone.0352449)
Supplement: Attachment — Submitted filename: PONE-D-25-25720.pdf [file pone.0352449.s003.pdf]

PLOS ONE

Demonstrating conservation impacts in California Marine Protected Areas using sophisticated statistical analysis of large-scale participatory science data

--Manuscript Draft--

Manuscript Number:	PONE-D-25-25720
Article Type:	Research Article
Full Title:	Demonstrating conservation impacts in California Marine Protected Areas using sophisticated statistical analysis of large-scale participatory science data
Short Title:	Conservation impacts in Marine Protected Areas from analysis of large scale participatory science data
Corresponding Author:	M.V. Eitzel<br>University of California Davis<br>Davis, CA UNITED STATES OF AMERICA
Keywords:	Marine Protected Areas; Citizen Science; community science; Participatory Sciences; California; Coastal use; human activities; hierarchical statistical modeling; zero-inflation; dispersion; generalized linear mixed models; semi-structured data; model-based inference; recreation; fishing
Abstract:	Measuring human interactions with protected areas is a key need in conservation social science, both to assess the potential for continuing negative impacts on sensitive resources and to support positive relationships with important places. Our paper investigates human activities in California Marine Protected Areas (MPAs) using an emerging approach of large-scale participatory data collection coupled with statistical analysis. Twelve organizations across the state developed, standardized, and implemented a data collection protocol for volunteers to record human activities, called ``MPA Watch.'' From 2012-2020, approximately 1,900 surveyors conducted more than 31,700 surveys, observing more than 1.2 million activities at 104 different sites across California's coast, both inside and outside MPAs. We analyzed these data using generalized linear mixed models with zero inflation to account for sampling bias associated with volunteer-driven data. We included covariates regarding weather and tides; time of day, week, and year; and beach type and amenities. We found statistically significantly lower consumptive activities inside MPAs with the strictest protections as compared with non-MPAs, and no significant difference between MPAs and non-MPAs for non-consumptive recreational activities. Some consumptive activities significantly declined over time. We also detected typical use patterns (e.g. for all activity categories, weekend counts were significantly higher than weekday counts). In addition, we noted patterns of interest for future study, including much higher incidence of non-consumptive activities than consumptive activities at a statewide level; higher popularity of some activities at certain individual sites; and the beginnings of the effects of the COVID-19 pandemic in 2020. Our analysis demonstrates that consumptive activities are lower inside California MPAs, and results at particular sites can inform site-specific management strategies. Further, we show the power and potential of large-scale participatory science, coupled with appropriate statistical modeling, to monitor and inform conservation policy and management for protected areas.
Order of Authors:	M.V. Eitzel
	Nick Ulle
	Ryan Meyer
	Ben Goldstein
Additional Information:	
Question	Response
Financial Disclosure<br><br>Enter a financial disclosure statement that	Yes

Powered by Editorial Manager® and ProduXion Manager® from Aries Systems Corporation

describes the sources of funding for the work included in this submission. Review the submission guidelines for detailed requirements. View published research articles from *PLOS ONE* for specific examples.<br><br>This statement is required for submission and **will appear in the published article** if the submission is accepted. Please make sure it is accurate.<br><br>**Funded studies**<br>Enter a statement with the following details:<br>• Initials of the authors who received each award<br>• Grant numbers awarded to each author<br>• The full name of each funder<br>• URL of each funder website<br>• Did the sponsors or funders play any role in the study design, data collection and analysis, decision to publish, or preparation of the manuscript?<br><br>Did you receive funding for this work?	
Please add funding details.<br>as follow-up to "**Financial Disclosure**<br><br>Enter a financial disclosure statement that describes the sources of funding for the work included in this submission. Review the submission guidelines for detailed requirements. View published research articles from *PLOS ONE* for specific examples.<br><br>This statement is required for submission and **will appear in the published article** if the submission is accepted. Please make sure it is accurate.	RM received funding from the Resources Legacy Fund (grant numbers 13884 and 17582, https://resourceslegacyfund.org/)<br><br>The funder was involved in framing the questions of the study and the presentation of results, but did not exert undue influence that would downplay or bias against the presentation of neutral or negative results, which are also included in the manuscript. In addition, positive claims about Marine Protected Area effectiveness were highly supported by the data.

Powered by Editorial Manager® and ProduXion Manager® from Aries Systems Corporation

**Funded studies**<br>Enter a statement with the following details:<br>• Initials of the authors who received each award<br>• Grant numbers awarded to each author<br>• The full name of each funder<br>• URL of each funder website<br>• Did the sponsors or funders play any role in the study design, data collection and analysis, decision to publish, or preparation of the manuscript?<br><br>Did you receive funding for this work?"	
Please select the country of your main research funder (please select carefully as in some cases this is used in fee calculation).<br>as follow-up to "**Financial Disclosure**<br><br>Enter a financial disclosure statement that describes the sources of funding for the work included in this submission. Review the submission guidelines for detailed requirements. View published research articles from *PLOS ONE* for specific examples.<br><br>This statement is required for submission and **will appear in the published article** if the submission is accepted. Please make sure it is accurate.<br><br>**Funded studies**<br>Enter a statement with the following details:<br>• Initials of the authors who received each award<br>• Grant numbers awarded to each author<br>• The full name of each funder<br>• URL of each funder website<br>• Did the sponsors or funders play any role in the study design, data collection and analysis, decision to publish, or preparation of the manuscript?<br><br>Did you receive funding for this work?"	UNITED STATES - US
**Competing Interests**	The project involved close collaborations with funders, decision makers, and

Use the instructions below to enter a competing interest statement for this submission. On behalf of all authors, disclose any competing interests that could be perceived to bias this work—acknowledging all financial support and any other relevant financial or non-financial competing interests.<br><br>This statement is required for submission and **will appear in the published article** if the submission is accepted. Please make sure it is accurate and that any funding sources listed in your Funding Information later in the submission form are also declared in your Financial Disclosure statement.<br><br>View published research articles from *PLOS ONE* for specific examples.<br><br>**NO authors have competing interests**<br><br>Enter: *The authors have declared that no competing interests exist.*<br><br>**Authors with competing interests**<br><br>Enter competing interest details beginning with this statement:<br><br>*I have read the journal's policy and the authors of this manuscript have the following competing interests: [insert competing interests here]*<br><br>* typeset	community groups that want to improve and publicize the effectiveness of marine conservation. Therefore many conversations focused on results that could be useful in advancing that objective. All authors were compensated from the grants supporting this work, but the funder did not exert undue influence that would downplay or bias against the presentation of neutral or negative results, which are also included in the manuscript, and positive claims about Marine Protected Area effectiveness were highly supported by the data.
**Ethics Statement**<br><br>Enter an ethics statement for this submission. This statement is required if the study involved:<br><br>• Human participants<br>• Human specimens or tissue<br>• Vertebrate animals or cephalopods<br>• Vertebrate embryos or tissues	This study makes use of pre-existing data and does not collect any new data on human subjects. In addition, the data are available publicly online. Any information about volunteer surveyors that collected the data was removed from the database before we analyzed it; and the humans engaging in coastal activities that the surveyors are observing and counting are in public places. Thus, we did not have access to information that could identify individual participants during or after data collection. Given this, we have not sought Institutional Review Board Human Subjects review.

Powered by Editorial Manager® and ProduXion Manager® from Aries Systems Corporation

• Field research<br><br>Write "N/A" if the submission does not require an ethics statement.<br><br>General guidance is provided below. Consult the submission guidelines for detailed instructions. **Make sure that all information entered here is included in the Methods section of the manuscript.**	

Powered by Editorial Manager® and ProduXion Manager® from Aries Systems Corporation

**Format for specific study types**<br><br>**Human Subject Research (involving human participants and/or tissue)**<br>• Give the name of the institutional review board or ethics committee that approved the study<br>• Include the approval number and/or a statement indicating approval of this research<br>• Indicate the form of consent obtained (written/oral) or the reason that consent was not obtained (e.g. the data were analyzed anonymously)<br><br>**Animal Research (involving vertebrate animals, embryos or tissues)**<br>• Provide the name of the Institutional Animal Care and Use Committee (IACUC) or other relevant ethics board that reviewed the study protocol, and indicate whether they approved this research or granted a formal waiver of ethical approval<br>• Include an approval number if one was obtained<br>• If the study involved *non-human primates*, add *additional details* about animal welfare and steps taken to ameliorate suffering<br>• If anesthesia, euthanasia, or any kind of animal sacrifice is part of the study, include briefly which substances and/or methods were applied<br><br>**Field Research**<br><br>Include the following details if this study involves the collection of plant, animal, or other materials from a natural setting:<br>• Field permit number<br>• Name of the institution or relevant body that granted permission	
**Data Availability**<br><br>Authors are required to make all data underlying the findings described fully available, without restriction, and from the time of publication. PLOS allows rare exceptions to address legal and ethical concerns. See the PLOS Data Policy and FAQ for detailed information.	Yes - all data are fully available without restriction

Powered by Editorial Manager® and ProduXion Manager® from Aries Systems Corporation

A Data Availability Statement describing where the data can be found is required at submission. Your answers to this question constitute the Data Availability Statement and **will be published in the article**, if accepted.<br><br>**Important:** Stating 'data available on request from the author' is not sufficient. If your data are only available upon request, select 'No' for the first question and explain your exceptional situation in the text box.<br><br>Do the authors confirm that all data underlying the findings described in their manuscript are fully available without restriction?	
**Describe where the data may be found in full sentences. If you are copying our sample text, replace any instances of XXX with the appropriate details.**<br><br>• If the data are **held or will be held in a public repository**, include URLs, accession numbers or DOIs. If this information will only be available after acceptance, indicate this by ticking the box below. For example: *All XXX files are available from the XXX database (accession number(s) XXX, XXX.*).<br>• If the data are all contained **within the manuscript and/or Supporting Information files**, enter the following: *All relevant data are within the manuscript and its Supporting Information files.*<br>• If neither of these applies but you are able to provide **details of access elsewhere**, with or without limitations, please do so. For example:<br><br>*Data cannot be shared publicly because of [XXX]. Data are available from the XXX Institutional Data Access / Ethics Committee (contact via XXX) for researchers who meet the criteria for access to confidential data.*<br><br>*The data underlying the results presented in the study are available from (include the name of the third party*	The data underlying the results of this paper are available at Dryad under accession number [TBD]. The code used to generate results and figures are available with a GPL3 license on GitHub at [repository address TBD].

Powered by Editorial Manager® and ProduXion Manager® from Aries Systems Corporation

and contact information or URL).<br>• This text is appropriate if the data are owned by a third party and authors do not have permission to share the data.<br><br>* typeset	
Additional data availability information:	Tick here if the URLs/accession numbers/DOIs will be available only after acceptance of the manuscript for publication so that we can ensure their inclusion before publication.

Powered by Editorial Manager® and ProduXion Manager® from Aries Systems Corporation

Manuscript

Click here to access/download;Manuscript;MPAWatchCountAnalysisPaper.pd

Demonstrating conservation impacts in California Marine Protected Areas using sophisticated statistical analysis of large-scale participatory science data

MV Eitzel[1,2*], Nick Ulle[3], Ryan Meyer[2], Ben Goldstein[4]

1 Feminist Research Institute, University of California, Davis, Davis, CA, United States of America
2 Center for Citizen and Community Science, University of California, Davis, Davis, CA, United States of America
3 DataLab, University of California, Davis, Davis, CA, United States of America
4 Department of Forestry and Environmental Resources, North Carolina State University, Raleigh, NC, United States of America

* mveitzel@ucdavis.edu

Abstract

Measuring human interactions with protected areas is a key need in conservation social science, both to assess the potential for continuing negative impacts on sensitive resources and to support positive relationships with important places. Our paper investigates human activities in California Marine Protected Areas (MPAs) using an emerging approach of large-scale participatory data collection coupled with statistical analysis. Twelve organizations across the state developed, standardized, and implemented a data collection protocol for volunteers to record human activities, called “MPA Watch.” From 2012-2020, approximately 1,900 surveyors conducted more than 31,700 surveys, observing more than 1.2 million activities at 104 different sites across California’s coast, both inside and outside MPAs. We analyzed these data using generalized linear mixed models with zero inflation to account for sampling bias associated with volunteer-driven data. We included covariates regarding weather and tides; time of day, week, and year; and beach type and amenities. We found statistically significantly lower consumptive activities inside MPAs with the strictest protections as compared with non-MPAs, and no significant difference between MPAs and non-MPAs for non-consumptive recreational activities. Some consumptive activities significantly declined over time. We also detected typical use patterns (e.g. for all activity categories, weekend counts were significantly higher than weekday counts). In addition, we noted patterns of interest for future study, including much higher incidence of non-consumptive activities than consumptive activities at a statewide level; higher popularity of some activities at certain individual sites; and the beginnings of the effects of the COVID-19 pandemic in 2020. Our analysis demonstrates that consumptive activities are lower inside California MPAs, and results at particular sites can inform site-specific management strategies. Further, we show the power and potential of large-scale participatory science, coupled with appropriate statistical modeling, to monitor and inform conservation policy and management for protected areas.

Introduction

Protected areas are an important and widely used mechanism for conserving resources that are susceptible to overuse (the so-called and oft-disproven "tragedy of the commons" [1]). Fisheries and ocean resources can fall into patterns reminiscent of the tragedy of the commons, given their open-access nature [2] and though marine protected areas (MPAs) do not necessarily address the question of the global ocean commons [3], they can address some layers of a hierarchical, nested common governance problem [2]. MPAs have thus emerged as a cornerstone strategy in global ocean conservation efforts, with their implementation expanding significantly over the past two decades [4]. These designated zones, where human activities are regulated to protect marine ecosystems, now cover roughly 7% of the world's oceans, reflecting growing international commitments to marine conservation [5].

However, protected areas can be controversial due to the potential to include and exclude people unevenly and distribute benefits inequitably. [6] Therefore it becomes useful to ask not only what are the effects of protection on both use of the common resources that can be depleted or consumed (e.g. fisheries or plant resources), but also what are the effects on other, non-consumptive uses (e.g. local recreation or tourism)? As the scale of protected areas – marine or otherwise – is growing worldwide, collection of useful monitoring data that can inform both questions of resource protection and equitable access, as well as supporting MPA implementation and management, is a persistent challenge. [7, 8] The effectiveness of MPAs varies considerably depending on design, enforcement, and ecological context, and science-based monitoring is essential to understand MPA performance and optimize their conservation outcomes along multiple dimensions [9]. At the same time, MPAs are more effective when there is shared governance between government and non-government actors [10], and communication between staff associated with protected areas and nearby communities can be key to enhance these relationships [11]. So one way to find out how people are engaging with protected areas in general (and MPAs specifically) in both consumptive and non-consumptive ways, and to support communities impacted by protected areas, could be to engage directly with the relevant communities.

In order to both engage with community members and to address the challenge of collecting long-term monitoring data – sometimes over a large geographic scale – many have pointed to community and citizen science (CCS) as a promising approach [12–14]. In natural resources, CCS programs leverage public interest in common resources, such as wildlife or parks, and recruit volunteers to conduct surveys, sometimes in synergy with their own recreational activities [15]. Because they depend on volunteer effort, some CCS programs relax the strict protocols of traditional surveys to allow observers some flexibility in choosing when, where, and how to make observations. To date, CCS projects have yielded observational datasets that greatly exceed those data collected under structured surveys in both volume and coverage [16, 17]. Consequently, the use of these data in scientific research is rapidly increasing [17].

While CCS data have advantages for monitoring—namely their large volume and coverage relative to cost—these data can be difficult to analyze accurately [18]. The sampling process that generates these data is heterogenous, as participants vary in their effort, interests, and skill [19–21], which could bias downstream inference [18, 22]. Such strategies therefore shift efforts at developing credibility to the analytical phase, after data have been collected [23]. There are two broad approaches to making use of CCS-generated data. First, the CCS planners can introduce structure on the design side by requiring volunteers to follow particular methodologies to reduce heterogeneity. CCS programs must strike a balance between introducing enough structure to help standardize sampling protocols and maintaining accessibility and fun for participants. One popular design-level choice is to collect metadata describing their sampling, such as

the date and length of time of each survey. Datasets in which sampling heterogeneity is allowed and documented are known as “semi-structured” (in contrast to “structured” data with standardized protocols and also to “unstructured” data where heterogeneity is not documented) [17]. The second broad approach, known as model-based inference, uses statistical modeling to account for heterogeneity in the data generating process during analysis, for example by including observed confounding variables or grouping random effects in a multivariate linear mixed model. Semi-structured datasets, which explicitly document sources of heterogeneity, are well suited to analysis using model-based inference.

The goal of this study is to use model-based inference with semi-structured CCS data to answer questions about human activities in MPAs across the state of California during 2012-2020. Surveyors in the MPA Watch program [24] collect data following a set protocol, in predetermined locations, but sampling effort is highly variable across space and time, and some activities may not be observed in a given survey. We used a zero-inflated Generalized Linear Mixed Model (GLMM), including a variety of predictors controlling for seasonality, spatial relationships, and features associated with survey locations, in order to address two key questions for each of seven broad human activity categories: 1) Are there differences in activity in MPAs with no ‘take’ allowed, MPAs with more varied rules about what visitors can remove, and non-MPAs with no restrictions? 2) Are there differences over time in activity counts, and do differences depend on MPA type? We also ask: 3) What can MPA Watch data tell us about potential violations of the Marine Life Protection Act? 4) What are the seasonal, diurnal, and weekly patterns in actvity? 5) What features of individual locations affect human activities?

Materials and methods

Study system: California Marine Protected Areas (MPAs) and MPA Watch

In California, a network of 126 marine protected areas have been established in coastal waters, under the auspices of California’s Marine Life Protection Act (MLPA) [25]. Multiple state agencies and other partner organizations, including federal agencies, private funders, and other organizations, have collaborated to implement and manage the network [26, 27]. The MLPA calls for adaptive management of MPAs, for the benefit of ocean health both inside and outside of MPAs. Monitoring is a crucial component of measuring progress toward MLPA objectives, which include opportunities for recreation, education, and scientific study, in addition to improved biodiversity and ecological health [25]. The state has invested millions of dollars in monitoring projects cutting across a wide range of disciplines and focal areas [28]. A notable feature of the state’s approach to MPA monitoring has been the emphasis – from the beginning – on CCS approaches that can integrate with or supplement traditional professionalized approaches [29–32].

The data underlying our model come from surveys run by the Marine Protected Area (MPA) Watch program. MPA Watch is a community science network, with participation by non-profits, state agencies, and tribal groups throughout the California Coast. In its first decade of operation MPA Watch did not receive funding directly from the state as part of the MPA monitoring program, but it has received considerable support from private funders that collaborate with the state on the shared project of MPA implementation. While there have been a handful of state-funded research projects focused on human dimensions of California’s MPAs [33, 34], MPA Watch is the only ongoing monitoring program on this topic, and the only one that examines both

consumptive and non-consumptive human uses at a statewide scale. In recent years the California Ocean Protection Council has supported MPA Watch through grants that support capacity building and analysis, including the study reported on here. It is important to note that both state and private funders of this project are also active participants in the collaborative implementation of MPAs.

MPA Watch activity count survey protocol

In the MPA Watch program, each of twelve participating organizations is responsible for training adult (over 18) volunteers, who then follow a statewide protocol designed to consistently gather counts of consumptive and non-consumptive human activities at coastal sites throughout California. The program began in Monterey county in 2011 following the establishment of the new network of MPAs in state waters (under the auspices of the 1999 MLPA), and by 2016 had spread to organizations and sites in every county of the state (Fig 1). MPA Watch is intended to inform management and implementation of MPAs, while also educating local residents about marine conservation and engaging them in stewardship – in alignment with the State's partnership-based approach to MPA implementation [27]. Since agreeing upon a statewide protocol, MPA Watch has gathered more than 49,000 surveys across 203 transects within 103 sites (as of June 2024), amounting to a unique and rich source of data about human activities. MPA Watch data could also be used for more than just enforcement of the MLPA and for education of coastal users; analyses could potentially speak to a wide range of human dimensions research topics on coastal human actvities. Local organizations and communities, too, could make use of MPA Watch data to understand the pressures on and opportunities associated with their coastal places. The current study supports these potential goals by investigating what is possible when analyzing the MPA Watch archive with appropriate model-based inference.

Fig 1. Sites and sampling effort over time. The full California Marine Protected Area network includes over 200 sites across the entire coast; shown at left are the 102 sites with MPA Watch surveys analyzed in this paper. On the right is the number of surveys per quarter for each site (ordered from most northern at the top through most southern at the bottom). Data collection was fairly widespread starting in 2014, with some sites in northern California starting only recently. Note the gap in data collection in Q2 2020, during the shelter-in-place orders of the COVID-19 pandemic.

Table 1. Activity categories from the MPA Watch surveys. "Consumptive" refers to the removal of materials from the Marine Protected Area or other coastal place.

Activity Category	Description
Onshore recreation	Shore-based recreation (except tidepooling).
Domestic animals	On- and off-leash domestic animals (mostly dogs); people accompanying the animals are counted in "onshore recreation".
Tidepooling	Shore-based non-consumptive tidepooling.
Onshore fishing	Shore-based hook & line, trap, net, and spear fishing.
Offshore recreation	Swimming, non-consumptive SCUBA or snorkeling, non-consumptive diving, and board sports (surfing, boogie-boarding, stand-up paddleboarding, kitesurfing, windsurfing).
Offshore fishing[1]	Boat-based hook & line, trap, net, spear, and dive fishing; recreational, passenger, and commercial; active only (boats not actively fishing are allowed to transit through MPAs). Counts of boats, not people.
Recreational boating[1]	Paddle-operated boats, kayaks, power boats, sail boats, jet skis, dive boats, whale-watching boats, and sport fishing boats. Counts of boats, not people.
Hand-collection	Collection of biota, e.g. removing invertebrates from a tidepool.
Total activities[1,2]	Aggregates all the above categories, plus additional more infrequent activities reported on the survey sheet; see for a full list.

[1] Uses data from both boat-based and land-based surveys.
[2] Not statistically modeled because it is an aggregate of other categories.

In the protocol, MPA Watch surveyors walk a pre-set transect along the coastline, tallying counts of human activities on a datasheet with predefined categories. Counts are largely of individual humans, although there are also categories for domestic animals and boats of various types. Observers record a variety of metadata, including weather conditions, tides, date and time, and site features. Although the sites and transects are pre-defined, surveyors engage in the activity in a timeframe and level of frequency that works best for them personally. Some programs also survey human activities from a boat for particular transects, and refrain from counting certain shore-based activities that are difficult to discern from that vantage point. (Some of these sites have corresponding land-based surveys as well.) We only included boat-based surveys for offshore recreation and recreational boating (which are themselves counts of boats, not people). For a detailed accounting of the data collection protocol, see for the MPA Watch training manual.

In our analysis, the specific categories of activities from the surveys (see example data sheet in) have been grouped into larger categories (Table 1). By aggregating activity categories, we avoid the difficulty with fine classifications of different activities that may vary in different parts of California, and by choosing an appropriate scale of categorization we also avoid some of the statistical difficulties associated with activity categories that are rare.

One topic of great interest to both the State of California and to MPA managers is compliance with the Marine Life Protection Act (MPLA). In order to inform this question, we pay special attention to the prevalence of consumptive activities (onshore fishing, offshore fishing, and hand collection of biota) specifically in no-take MPAs,

where any consumptive activity is a potential violation of the MLPA. These are only potential violations because the protocol does not include mechanisms for determining whether the take was actually legally permitted, e.g. if they were engaging in Tribal cultural practices or scientific studies. The full protocol and dataset also include the surveyors' assessment of whether a violation is occurring, but this was less consistently applied across different observers. Therefore we focus on this definition of a potential violation because we were able to consistently apply it across the dataset.

Predictor variables

Transects were set up both within MPA sites throughout the length of California's coast, and in non-MPA sites, to allow comparisons between locations that have been designated as protected versus those that have not. These non-MPA sites are not necessarily chosen to deliberately contrast with specific MPAs, so our analysis does not rely on paired tests and instead treats them as separate groups. Instead we categorize each transect as within either 1) a non-MPA site, 2) a no-take MPA (State Marine Reserves and no-take State Marine Conservation Areas, because they have the strictest and most consistent rules regarding what visitors are allowed to do), and 3) any other type of MPA (considered together as one group). We also include the year of the survey as a factor. For recreational boating and offshore fishing, we also include boat-based surveys, and include a factor in the statistical models to control for the difference in the land-based versus boat-based protocols.

We discussed potential predictor variables with MPA Watch volunteer managers, and included variables they judged to be important, as well as those that we felt were important to control for based on the experimental design. These variables related to the survey process itself, the timing of the survey, the location of the survey, the characteristics of the transects, and weather and tide conditions at the time of the survey. We standardized all continuous predictors (subtracted the mean and divided by the standard deviation) to improve the optimizer's ability to find a maximum likelihood estimate.

Table 2 lists all the potential predictors in our models (not all predictors were included for every activity; see the results section for which variables were included for which activity categories). See for additional detail on the data handling or construction of the predictor variables.

To prepare for modeling, we organized the data into a matrix with each row representing a survey associated with an observed count for each category in Table 1 and values for all predictors listed below (data downloaded on March 14, 2022). We omitted records in which a surveyor did not provide information on all predictors (e.g. weather conditions) reducing the number of records from 29,353 to 27,088 for only land-based surveys and from 31,725 to 29,413 when boat-based surveys are included. We included all transects in our analysis, but in figures we did not display the parameter estimate for transects associated with fewer than 10 surveys.

Note that this study makes use of pre-existing data and does not collect any new data on human subjects. In addition, the data are available publicly online. Any information about volunteer surveyors that collected the data was removed from the database before we analyzed it; and the humans engaging in coastal activities that the surveyors are observing and counting are in public places. Thus, we did not have access to information that could identify individual participants during or after data collection. Given this, we have not sought Institutional Review Board Human Subjects review.

Table 2. Predictors included in models of activity counts. "Scale" refers to whether the variable pertains to one individual survey, all records associated with a given transect, or all records associated with a given site.

Name	Units	Scale	Description
Key Questions			
MPA status	Categorical	Site	Non-MPA, no-take MPA, some-take MPA
Pattern over time	Categorical	Survey	Categorical factor for each year (2012 through 2020)
Time × MPA status	Categorical	Survey	Interaction between MPA type and categorical year, allowing for different time patterns for each category.
Site/Transect Features			
Access/amenities index	Unitless	Transect	Index ranging from 2 to 14 (see below and in)
Beach type	Categorical	Transect	"Sandy," "Rocky," or "Sandy and Rocky"
Has tidepooling	Categorical	Transect	Is the transect a site known for tidepooling? (yes/no)
Population density	Persons/km^2	Site	Mean over 2012-2019 for census tracts within 15 miles
State park proximity	Categorical	Transect	Is the transect immediately adjacent to a State Park? (TRUE/FALSE)
Spatial Variables			
Latitude	Degrees	Transect	Includes polynomial terms from degree 2 up to 8 depending on the activity
Site	Categorical	Site	Random effect to reflect site-level variation not captured by other covariates
Transect	Categorical	Transect	Random effect to reflect transect-level variation not captured by other covariates
Temporal Variables			
Day of week	Categorical	Survey	Sat, Sun, Mon, Tues, Wed, Thu, Fri
Time of day	Hrs & Min	Survey	Time of the survey start; linear and quadratic terms
Seasonality	Day of year	Survey	Represented by two variables ("winterness" and "springness;" see below and in)
Survey Method Variables			
Survey duration	Minutes	Survey	Time spent surveying, typically one hour
Transect length	Miles	Transect	Length of transect as originally planned
Survey type	Categorical	Transect	Boat-based or Land-based (only for some categories, see Table 1)
Program	Categorical	Transect	Random effect to reflect variation from one MPA Watch program to another
Weather/Tide Conditions			
Tide level	Unitless	Survey	Scaled tide height (see below and in)
Clouds	Categorical	Survey	Clear, Partly Cloudy (1-50% cloud cover), or Cloudy (>50% cloud cover)
Precipitation	Categorical	Survey	Yes/no
Visibility	Categorical	Survey	Perfect (to horizon), limited (beyond shore, but not to horizon), or Shore Only

Generalized Linear Mixed Model implementation

We used Generalized Linear Mixed Models (GLMMs) to model counts of each human activity type. We modeled each activity category separately, following a negative binomial distribution, which can represent counts with dispersion using two different parameterizations (linear and quadratic). We included all predictor variables appropriate for each activity category in the "conditional" part of the model represented by the counts.

We estimated models using glmmTMB (version 1.1.10) [35] in R (version 4.4.2) [36]. We ensured that our results come from models with no convergence warnings, indicating

that the optimizer successfully found a maximum. To assess model fit and choose between model parameterizations, we used DHARMa (version 0.4.7) [37] to plot model residuals. We used iterative graphical checks to assess whether there were systematic deviations between the model and the observed data while selecting error families and the form of zero-inflation (see below). While the iterative process was largely qualitative, we made sure to choose models that had non-significant tests for zero-inflation and dispersion (implying that we had accounted for these statistical features within the models we had chosen). For some of the continuous variables, we included higher order polynomials to allow for more flexible functions of these variables; how many terms to include was based on observations of patterns in the residuals.

For our key questions regarding activities inside and outside MPAs based on the MPA's type of restriction, and the potential patterns across time, we also included an interaction between site type and year, in order to see how usage patterns may have changed differently over time for each category of site (no-take MPAs, some-take MPAs, and non-MPAs).

Dispersion & zero-inflation

By using a negative binomial distribution, we also able to account for overdispersion in observed counts, when there is more or less variation in counts than would be expected for a typical distribution (e.g. Poisson). In addition, predictor variables can be included in the zero-inflation and/or dispersion parts of the model – reflecting that some categories of sites have even more zeroes than others, or some categories have greater variation than other categories. For most activities we had only a constant overdispersion parameter. The meaning of the overdispersion parameter is different for different forms of the negative binomial distribution; we clarify which distribution was used for each activity category in the results table on dispersion and zero-inflation. We did consider models with predictors in the overdispersion portion of the model, but for most activity categories, the residuals were not much improved, so we chose to keep those models simpler. For hand collection of biota, we did include the beach type and whether it was a known tidepooling site in the overdispersion model. This reflects that surveys with different values of these predictors had higher or lower variation than others (for example, there could be more variation for a rocky transect than for a sandy one).

We also modified GLMMs to account for zero inflation, where the data include additional zero observations beyond what might be expected from the assumed distribution. For example, additional zeroes in MPA Watch surveys could be due to mismatch in timing between surveyors and coastal users (surveyors simply are not present when individuals are engaging in activities), and/or because surveyors check for all activities every time they survey and they could be doing so at a time or place which is appropriate for one activity but not another.

For most activity categories, we had a constant zero-inflation parameter, which means that there is a constant probability that a survey recorded 'extra' or 'structural' zeroes e.g. due to mismatch in timing between surveyor and activity – in addition to the underlying distribution of counts which could include some zeroes as part of the underlying process. For tidepooling, we did include predictors in the zero-inflation part of the model, which means that for example, sites that were not known for tidepooling were more likely to have zero tidepoolers than a site that was known for tidepooling (in addition to the fact that there might not be any tidepoolers at that site in that particular moment, e.g. if it were a less popular site for tidepooling, with lower counts overall). Onshore recreation, which is a much more common activity than the others that was essentially always present, was not zero-inflated.

Random effects

For all activity categories, we included nested Gaussian random effects for site and transect within site. These random effects are intended to account for additional unknown factors that could differ from transect to transect or site to site that were not accommodated by the other covariates in the model. We also included random effects for MPA Watch program (13 programs which include multiple sites; we are accounting for the possibility that programs may train volunteers in slightly different ways). We excluded the MPA Watch program random effect in four cases (offshore fishing, recreational boating, and animals), as including all three random effects resulted in overfitting (as observed in the DHARMa residuals).

Statistical inference

We used Wald tests to determine the statistical significance of the continuous predictor covariates and categorical variables with only two levels (e.g. yes/no) in the count, dispersion, the zero-inflation portions of the model. We used R's built-in "drop1" function to do likelihood ratio tests for categorical variables with more than two levels (e.g. three different MPA types or days of the week) and Tukey tests (R function "glht" in package "multcomp" (version 1.4.28) [38] for multiple comparisons between different levels of categorical variables (e.g. the differences between days of the week and differences between MPA types). Because this resulted in 624 tests total between all activity categories, we applied a multiple testing correction to all p-values within a given activity category using the Benjamini-Hochberg "false discovery rate" correction. [39] This reduces the possibility that a particular test would appear significant by chance.

Aggregated Total Activities

For the "Total Activities" category, which includes all the other categories listed in Table 1 as well as other smaller categories that were too rare to be separately modeled, we display visual summaries of modeling results, but do not calculate statistical results. This category is included for illustrative purposes and to give context to the magnitude of the other category model predictions but in order to preserve the validity of p-values, we do not draw statistical conclusions regarding it because it includes data that have already been used for inference.

Results

We identified statistically significant logical seasonal patterns, higher midday counts, and higher weekend counts. Rain tended to lower counts, recreation activities were higher for sandy beaches, and low tide favored onshore activities. Population density and access/amenities did increase some activities, and onshore fishing was higher immediately next to a state park, while offshore fishing was lower. There was less consumptive activity in MPAs than outside, and we found declines in consumptive activities over some time periods.

In terms of general patterns that we observed but did not test, consumptive activity was overall much lower than nonconsumptive activity throughout the coast, regardless of MPA status. We also observed particular individual site patterns, relative popularity for particular kinds of activity, and regional patterns across space. We see the very beginning of the impacts of the COVID-19 pandemic potentially indicating higher human activity corresponding to the shelter-in-place orders. We first review the statistical results, and then the qualitative patterns.

Statistical results

Tables 3- 8 summarize the results for each activity. 'NS' indicates not significant (P>0.05), while an empty cell indicates that variable was not included for that activity category. '(+)' indicates a positive effect, while '(-)' indicates a negative effect. See for tables with p-values and parameter estimates.

Key questions: MPA effects and year-to-year differences

For many activities, there were no significant differences between MPA types or patterns over time. However, we found that for onshore fishing, no-take MPAs had significantly lower counts than either of the other two categories, and for most years, some-take MPAs had significantly lower counts than non-MPAs (Fig 2). For some-take MPAs, there were significantly fewer predicted onshore fishing counts in the middle of the time period than at the very beginning. For offshore fishing, for some of the middle years of the time period, there was significantly less fishing inside no-take MPAs than in non-MPAs.

Fig 2. Time trends (days since first survey) for each category of site (No-Take MPAs, "Other" MPAs with some take, and non-MPA sites). Grey bands indicate 95% predictive error intervals.

Features of transects/sites

Table 3 summarizes the results for access and amenities, beach type, whether the transect is known for tidepooling, whether it is adjacent to a State Park, and what the nearby population density is. Sandy beaches, and combinations of sandy and rocky beaches, had greater activity for onshore and offshore recreation as well as domestic animals. Recreation, onshore fishing, and domestic animals had higher counts for transects with more amenities and easier access. Tidepool counts were, as expected, higher for sites that were known for their tidepooling (regardless of their MPA status). Higher population density (average people/km over 2012-2019 within 15 miles) was associated with higher counts in recreational boating and domestic animals, but was not significant for other activities (which could indicate that a different metric of population density would be more appropriate for those activities). Finally, being immediately adjacent to a State Park was associated with higher onshore fishing counts and lower offshore fishing counts.

Spatial variables

In terms of linear trends in latitude, offshore recreation and hand collection showed higher counts for more southern sites; and recreational boating had a complex relationship with latitude, but with generally higher counts through middle latitudes (Table 4). In addition, random effect standard deviations for both sites as well as transects within sites were reasonably large, with the most variation occurring in tidepooling, indicating important additional spatial variation at a variety of scales. See below for specific comparisons of activity patterns across the state.

Temporal variables

Table 5 shows results for temporal variables. Weekends had greater counts than weekdays for all activities, and for several activities (offshore recreation, recreational boating, and onshore fishing) Fridays were significantly higher than other weekdays. For

Table 3. Results for transect-related features. Note that 'beach type' was not included in offshore fishing or recreational boating, and whether the site was a known tidepooling site was only considered for onshore recreation, tidepooling, and hand collection of biota. The access and amenities index, population density, and adjacency to a State Park were all considered for all activity categories.

Activity category	Access/ amenities	Beach type	Tidepooling	Population density	State Parks
Onshore Recreation	(+)	Sandy/Rocky > Rocky<br>Sandy > Rocky	NS	NS	NS
Offshore Recreation	(+)	Sandy/Rocky > Rocky<br>Sandy > Rocky		NS	NS
Onshore Fishing	(+)	NS		NS	(+)
Offshore Fishing	NS			NS	(-)
Tidepools	NS	NS	(+)[1]	NS	NS
Recreational Boating	NS			(+)	NS
Domestic Animals	(+)	Sandy/Rocky > Rocky		(+)	NS
Hand Collection	NS	NS	NS[2]	NS	NS

[1] The 'known tidepooling site' factor was not significant in the zero-inflation term, but we included it in the final models for theoretical reasons and due to better model fit via evaluation of residuals.
[2] The 'known tidepooling site' factor was significant in the dispersion term of the model for hand collection of biota, though not in the count portion of the model.

Table 4. Results for spatial variables.

Activity category	Latitude linear	Latitude squared	Latitude higher-order[1]	Site Random Effect	Transect Random effect
Onshore Recreation	NS	NS		0.8185	0.6867
Offshore Recreation	(-)	NS		1.0925	0.8786
Onshore Fishing	NS	NS		0.5765	0.9386
Offshore Fishing	NS	NS		0.9969	0.7840
Tidepools	NS	NS		1.6945	1.0613
Recreational Boating	NS	(-)	4th (+), 6th (-)	0.8446	0.9779
Domestic Animals	NS	NS	NS	0.9371	0.9556
Hand Collection	(-)	NS		0.8272	1.0005

[1] Domestic animals and recboating had terms up to 8th order, based on residual patterns.

other activity categories, some weekdays were also greater than others. Fishing categories peaked in the morning, and other activities in the afternoon, with domestic animals peaking as late as almost 5 pm. Onshore fishing was highest in late June, and onshore recreation, hand-collection, tidepooling, and animals were highest in early July, recreational boating and offshore recreation in late July, and offshore fishing in mid-September.

Survey method variables

Including variables representing survey design features (e.g. how long a transect was, whether it was conducted from a boat or from shore) generally improved model fit, and some were statistically significant in predicting counts (Table 6). Boat-based surveys observed significantly more offshore fishing than shore-based surveys. Transect length was not significant across most categories; however, for domestic animals the residuals warranted a squared term, and it was statistically significant and negative – implying that there were larger numbers of animals on transects with intermediate lengths. Survey duration was always significant, with longer duration surveys observing higher

Table 5. Results for temporal variables. For all activity categories, the linear and quadratic terms for time of day were statistically significant; we share an estimate of peak time of day. For our two time-of-year variables, "winterness" and "springness," one or both were significant for all activity categories so we share an estimate of peak time of year.

Activity category	Day of Week	Time of Day	Seasonality
Onshore Recreation	Weekend > Weekday<br>Fri > Tue, Wed, Thu<br>Mon > Thu	2:40 pm	Jul 9
Offshore Recreation	Weekend > Weekday<br>Fri > other weekdays	1:40 pm	Jul 30
Onshore Fishing	Weekend > Weekday<br>Fri > other weekdays	10:30 am	Jun 26
Offshore Fishing	Weekend > Weekday	10:00 am	Sep 14
Tidepools	Weekend > Weekday	2:10 pm	Jul 4
Recreational Boating	Weekend > Weekday<br>Fri > other weekdays	12:10 pm	Jul 27
Domestic Animals	Weekend > Weekday<br>Fri > Tue<br>Mon > Tue, Wed > Tue	4:50 pm	Jul 2
Hand Collection	Sat > Weekday<br>Sun > Mon, Wed, Thu	12:40 pm	Jul 6

counts, and for domestic animals, the same quadratic relationship largely held (medium-duration surveys had higher counts, though longer surveys still had higher counts than shorter surveys).

Random effects standard deviations accounting for variation from program to program were generally smaller than the transect and site random effects, and were highest for recreation, tidepooling, and hand-collection, and small for onshore fishing. This effect could point managers towards places where protocols may need to be realigned between programs. The other three categories' residuals were not improved by including this effect.

Table 6. Results for survey method variables. Note that only recreational boating and offshore fishing use both boat and shore-based surveys.

Activity category	Survey Type	Transect Length	Survey Duration	Program Random Effect
Onshore Recreation		NS	(+)	0.4648
Offshore Recreation		NS	(+)	0.6828
Onshore Fishing		NS	(+)	0.0003
Offshore Fishing	Boat > Land	NS	(+)	
Tidepools		NS	(+)	0.4362
Recreational Boating	NS	NS	(+)	
Domestic Animals		Medium-length = higher[1]	Medium-duration = higher[2]	
Hand Collection		NS	(+)	0.2925

[1] For domestic animals, the linear term was not significant, but we also included a quadratic term, which was significant.
[2] Both linear and quadratic terms were significant.

Tide level and weather

Table 7 summarizes the results for tide level, cloud cover, precipitation, and visibility.

Rain negatively impacted all activities but offshore fishing and hand collection, while most shore-based activities were impacted by tide levels. Higher counts of onshore recreation, tidepooling, domestic animals, and hand collection were all associated with lower tide levels; while onshore fishing, offshore recreation, and recreational boating showed no relationship.

Non-consumptive activities tended to have higher counts with lower cloud cover (clear, or $< 50\%$ cover), while consumptive activities tended to have higher counts with higher cloud cover (greater than 50% cover). Specifically, lower cloud cover was associated with more onshore and offshore recreation as well as tidepooling, domestic animals, and recreational boating. Onshore fishing was unaffected by cloud cover, while offshore fishing and hand collection were greater for greater cloud cover.

Limited visibility (either shore only, or some water visible, but not all the way to the horizon) was associated with increased onshore and offshore recreation, as well as tidepooling, while perfect visibility (all the way to the horizon) was associated with increased recreational boating, domestic animals, and offshore fishing. Onshore fishing and hand collection were unaffected by visibility conditions. This finding may reflect that when visibility is poor enough not to see any activity at all, surveyors do not do surveys. When visibility is not perfect, the weather may be more moderate, encouraging coastal visitation, or there could be less glare, improving the ability of surveyors to count activities. These ideas would need to be systematically tested, however.

Generally speaking, the relationship between activity and weather conditions is complex, in which cloud cover and visibility may have counterintuitive impacts on certain activities, but precipitation generally does result in fewer activity counts.

Table 7. Results for tide level and weather. Note that all of the weather variables were included for all activity categories, but tide level was not included for offshore fishing.

Activity category	Tide level	Clouds	Precipitation	Visibility
Onshore Recreation	(-)	clear > partly cloudy<br>partly cloudy > cloudy<br>clear > cloudy	(-)	limited > perfect
Offshore Recreation	NS	Clear > partly cloudy<br>Partly cloudy > cloudy<br>clear > cloudy	(-)	limited > perfect
Onshore Fishing	NS	NS	(-)	NS
Offshore Fishing		cloudy > partly cloudy<br>cloudy > clear	NS	limited > shore only<br>perfect > shore only<br>perfect > limited
Tidepools	(-)	clear > cloudy	(-)	limited > perfect
Recreational Boating	NS	Clear > partly cloudy<br>partly cloudy > cloudy<br>clear > cloudy	(-)	perfect > limited
Domestic Animals	(-)	partly cloudy > cloudy<br>clear > cloudy	(-)	perfect > limited
Hand Collection	(-)	cloudy > clear	NS	NS

Dispersion and zero-inflation

Many of the activity categories had dispersion parameters substantially larger than one (implying that there are many more larger counts than would be typicaly for the underlying distribution), with tidepooling and both onshore and offshore recreation in particular being strongly overdispersed (Table 8). Including dispersion (as a negative

binomial parameterized one of two different ways) improved the qualitative appearance of the residuals for all the categories, and statistical tests of the residuals indicated no additional dispersion. The domestic animals category was challenging to find a good distributional fit for, and therefore the dispersion is not large but also the least well-accounted for. Zero-inflation parameters were quite high for offshore fishing and domestic animals, meaning that there were many more surveys that observed none of these activities than would be expected for the underlying distribution (probably indicating that there were additional reasons not to observe those activities at those times/locations). But even for other categories, zero inflation was important in improving model fit (the residuals indicated no additional zero inflation was present once it was accounted for in the model).

Table 8. Results for dispersion and zero-inflation. Note that Onshore recreation is a common enough activity that it was not zero-inflated.

Activity category	Distribution	Dispersion parameter	Dispersion residual p-value	Zero-Inflation parameter	Zero-inflation residual p-value
Onshore Recreation	nbinom1	29.67	0.608	NA	0.176
Offshore Recreation	nbinom1	18.48	0.264	-3.458	0.696
Onshore Fishing	nbinom1	3.098	0.576	-2.193	0.912
Offshore Fishing	nbinom2	0.3029	0.480	-17.92	0.464
Tidepools	nbinom1	14.18	0.448	-1.398	0.984
Recreational Boating	nbinom1	2.762	0.952	-2.001	0.992
Domestic Animals	nbinom2	1.023	0.056	-16.06	0.880
Hand Collection	nbinom1	2.117	0.128	0.4304	0.888

Qualitative patterns and observations

As we observed in the raw data, there were more nonconsumptive activities than consumptive activities statewide, regardless of MPA type. Note in Figs 3 and 4 that the order of magnitude of model predictions for nonconsumptive activities are much larger than the consumptive activities.

Some time patterns emerged from our analysis that could be more systematically studied. For example, onshore and offshore recreation increased from 2013 to the middle of the time period, and then increased again in 2020 (See ; possibly due to the COVID-19 pandemic and shelter-in-place orders). A study framed specifically around the pandemic could make use of more recent data and methods developed for modeling the different phases of the pandemic. Another possible pattern is the drop in counts in the fishing categories from 2013 to the middle of the time period for the some-take MPAs. It is difficult to assess whether this is due to implementation of MPA restrictions phasing in over that time because no systematic activity data were collected before implementation. However, an analysis that included a variable for 'how long has this area been protected' (which would be the longest for many of the no-take MPAs, which have been protected in various ways prior to the MLPA) might enable us to test some of these patterns that may be related to implementation.

Regionally, onshore recreation is high in the San Francisco Bay Area ("Bay Area") and in the Los Angeles and San Diego areas – but domestic animals are particularly prevalent in the Bay Area and tidepooling is higher in the Los Angeles and San Diego areas (though Duxbury Reef State Marine Conservation Area in the north, and Montara State Marine Reserve and Asilomar State Marine Reserve in the central coast are high, in addition to Crystal Cove State Marine Conservation Area in the South). Onshore fishing is more common in the center of the state and in the north, relative to the south,

while the reverse is true of offshore fishing.

There are particular hotspots of total activity in San Francisco (Ocean Beach), near Santa Barbara (Carpinteria), and in San Diego (Matlahuayl State Marine Reserve and Swami's State Marine Conservation Area). At Matlahuayl, this is driven largely by recreational boating and offshore recreation, and Carpenteria is popular for hand collection of biota (as is Tijuana River in San Diego, and Brazil beach just north of the San Francisco Bay Area).

Fig 3. Counts across all sites in California for all consumptive activities, predicted from our models under favorable conditions (sunny weather at midday, mid-summer, on a weekend day, with average tide level). Each point is the average prediction for all transects within that site, for one-hour, one-mile-long transects; sites are arranged from north at the left to south at the right (see Fig 1 for the actual geographical arrangement of sites, but certain locations in California are shown for reference: EU = Eureka, SF = San Francisco Bay Area, SB = Santa Barbara, LA = Los Angeles, SD = San Diego). A) Predictions for all activites on the survey sheet, shown for context with the other activities. Below the total activities are predictions for B) hand collection of biota (e.g. removing invertebrates from a tidepool), C) onshore fishing (using a variety of different gear), and D) Offshore fishing (using a variety of different types of gear; note that this is a prediction of number of boats, not individual people).

Fig 4. Counts across all sites in California for all non-consumptive activities, predicted from our models under favorable conditions (sunny weather at midday, mid-summer, on a weekend day, with average tide level). Each point is the average prediction for all transects within that site, for one-hour, one-mile-long transects; sites are arranged from north at the left to south at the right (see Fig 1 for the actual geographical arrangement of sites, but certain locations in California are shown for reference: EU = Eureka, SF = San Francisco Bay Area, SB = Santa Barbara, LA = Los Angeles, SD = San Diego). A) Predictions for all activites on the survey sheet, shown for context with the other activities. Below the total activities are predictions for B) onshore recreation, C) domestic animals (a count of the animals themselves, usually dogs; the humans associated with them are counted in onshore recreation), D) Tidepooling, C) Recreational boating (for example, kayaking; note that this is a prediction of number of boats, not individual people), and D) offshore recreation (for example, SCUBA diving or surfing).

Discussion

Complex community and citizen science data can be challenging to model but such efforts can be very fruitful when appropriate modeling approaches are selected. Our work with MPA Watch program and its data offers several key insights, stemming from both the collaborative process, and the results of the analysis.

First, the collaborative modeling process does add its own complexity and lengthens the timeline while also adding new needs for specific answers from partners, but also leads to modeling that is better founded in the contextual realities of the system, and results that are more relevant to stakeholders' questions. Iterative feedback loops between the community science network, analysts, and marine protected area managers, helped identify appropriate research questions, improved analytical approaches, and increased the utility of results in terms of management actions and potential program improvements.

Second, our analysis allows MPA Watch data to speak to important policy questions, e.g. that compliance with MPA regulations is high. Levels of consumptive activity in no-take MPAs are very low across the board, especially relative to overall activity levels. The vast majority of human activities along the coast – inside and outside of MPAs – are non-consumptive. These high-level takeaways, along with more granular results for specific sites or activities, can be of use to the broad array of actors engaged in marine conservation in coastal California.

Lessons learned working with large CCS datasets

Our earlier efforts to work with the MPA Watch data centered on an occupancy model [24], which revealed some patterns about human coastal use, but we were unable to accommodate survey-level information (because surveys were aggregated in order to estimate detection probability along with presence/absence of a given activity). This aggregation led to us only being able to model very broad patterns (e.g. whether at least one person was doing that activity at some point in a given year in a given location). We embraced the generalized linear mixed model framework in this analysis because it allowed us to include the abundance of activities, as well as variables like time of day, weather and tide conditions, and much more that was elided when the surveys were aggregated. And indeed, some of the results were good validation of the method (e.g. weather and time patterns were as expected), thus building confidence in the results that address the key questions about activities inside and outside different types of MPAs. We were also better able to show differences between sites and to make clearer statements about the potential violations in no-take MPAs.

The reason, however, that we had initially used an occupancy model was that some of the predictor variables (for example, visibility) could impact both detection as well as abundance, in which the surveyors may have more difficulty seeing an activity with poor visibility, but also people may choose not to do that activity when visibility is poor. Models that account for the separate impact of predictors on detection and abundance, like N-mixture models, are a potential option for this dataset; however, even more data would be required to parameterize detection via repeat measurements within a given timeframe. We attempted to create an N-mixture model for the dataset as it currently stands, but just as with the occupancy model, in order to estimate detection, we had to aggregate to a spatial unit that was too coarse to provide meaningful information, and as with the occupancy model, survey-level variables were hard to include. Future work could explore how much data might be necessary to estimate the effects of detection separately from abundance – perhaps there is a strategy that can incorporate the richness of the survey-level data but also allow for the estimation of the detection probability separately from the abundance.

Another potential direction for future work is to use causal inference methods to study whether MPA status causes differences in activity levels over time. MPA Watch constitutes a quasi-experiment [40]: sites were not assigned MPA status (the treatment) randomly. Because MPA Watch began after the establishment of MPAs, there are not pre-treatment data with which to measure a post-treatment effect of MPA assignment. However, each MPA has a different date of establishment, so creating a variable to represent that could be fruitful, and ongoing MPA Watch data collection does enable year-on-year comparisons in trends between MPA and non-MPA sites, as well as estimation of effects of policy interventions or events going forward, by methods such as difference-in-differences [41] or synthetic control [42]. For instance, one could use this approach to investigate the impact of the COVID-19 pandemic in 2020 on human activity at coastal sites. In addition to enabling new inferences, we believe taking a rigorous causal inference approach to our present findings would provide a valuable check on them. Moreover, asserting a causal model would potentially make results easier to interpret and guide tests for compatibility between the model and the data (e.g., placebo tests and simulation studies).

Collaborative modeling process

A key feature of this process was the collaborative approach. Co-production of science is widely acknowledged as a time- and resource-intensive approach to science, which can yield benefits such as increased trust among collaborators, improved shared understanding, and greater salience and utility of the resulting scientific products for management, decision making, and other purposes. [43–46] Our own anecdotal experience aligns with this broader literature. The technical and analytical components of this work would have been of far lower quality without iterative engagement with the organizations responsible for implementing MPA Watch, and far less useful without iterative engagement with both MPA Watch and MPA management partners. For example, our approach to analyzing potential violations is the result of many conversations. Some of these focused on the on-the-ground realities of conducting an MPA Watch survey, the variation in surveyor practices with respect to potential violations, and realities of how those are reflected on individual data sheets and in the centralized database. Other conversations focused on what kinds of specificity and uncertainty were useful or tolerable to state agencies with a direct interest in MPA enforcement and compliance. A program of this scale and complexity relies on a highly technical modeling approach to account for the inherent "messiness" of the data. But by the same token, the analysts rely on partners to guide them through an understanding of both the data, the context of its acquisition, and the nature of the system that we are attempting to describe through our analytical efforts.

The complexity of the data meant that it was difficult for the group collecting the data to engage effectively with it, particularly at a synthetic, statewide level. Even with an experienced collaborative modeling researcher, the data and the resulting model's complexity made grasping the results difficult. Each MPA Watch program produces a report twice a year showing their data, but our analysis is the first time the managers have engaged with the data across the state. The iterative process did mean that we often thought we had resolved a particular problem or made a modeling choice that we then found ourselves revisiting later, even up to the end of the analysis phase. And while the process of using the data raised some technical questions regarding the drift of the protocol over time as different programs have begun to train surveyors slightly differently, our ability to include random effects for site, transect, and program allowed us to absorb some of these differences. The process of using the data revealed these kinds of differences, offering the managers the opportunity to realign their protocols (a process they have engaged in already, but one that this analysis helps to underline the

importance of). These alignment discussions have also raised the point that the California coast is wildly varied from north to south, not just in terms of activities, population, and infrastructure but also in terms of geology, ecology, and weather. This emphasizes the challenges inherent in a statewide analysis.

Future modeling efforts will extend what we have done in terms of the statewide analysis, possibly addressing the patterns of coastal use associated with the COVID-19 pandemic and/or integrating other datasets on MLPA violation citations with the MPA Watch observations of potential violations. In addition, some modeling and data visualizations may focus on specific programs or regions, motivated by questions from managers and surveyors on the ground.

Implications for conservation

MPA Watch data demonstrates that the vast majority of activity on the California coast is non-consumptive, and although fishing is an important livelihood and pastime, it is not the dominant activity even in unprotected places. This result, while not surprising in broad strokes, lends a quantiative entrypoint for both understanding this relationship between consumptive and nonconsumptive uses, and monitoring it over time. Our results also show that people are recreating in MPAs at the same rates as in other non-MPA sites. This result implies while people are engaging with protected areas, there is no evident statewide correlation or effect of the protected areas on recreation patterns, which is directly relevant to the management goal of enhancing recreational opportunities. On the other hand, this result suggests that protecting the subtractable resource of the commons does not, at the statewide level, interfere with the non-subtractable uses. In another recent innovative use of MPA Watch data [47], we can see that some "charismatic" sites do appear to attract disproportionate numbers of visitors for certain biodiversity-related activities. When considering possible changes to the network of MPAs (as is currently happening in the case of California MPAs), MPA Watch data can thus yield helpful "big picture" perspectives, while also offering the ability to zoom in on very specific places or questions.

Compliance with MPA regulations is a major interest of state agencies and other partners working together to implement MPAs. For any discussion of compliance, it is important to note that MPA Watch data only reveal "potential" violations (volunteers are not trained in law enforcement, and as a rule do not investigate any potential violations they observe). With that said, our analysis suggests that overall compliance with the Marine Life Protection Act is quite high: the vast majority of counted activities do not appear to violate MPA regulations. However, there may be places where attention is warranted to more outreach and enforcement. Our analysis reveals particular locations where potential violations are high, either or relative to rates of other activities at that location, or relative to potential violation rates at other sites. Even in these cases, the scale of the problem is small relative to the overall coastal use at those locations, but these results may still be useful in shaping outreach, education, and enforcement activities.

Take, for example, Asilomar State Marine Reserve (Fig 5. The model predictions show approximately 154 people there (for all activities) on a day with good weather conditions in a popular time of year, and under those same conditions, the models predict 7 people tidepooling and only 0.4 people hand-collecting. It has one of the highest predictions for onshore fishing of a no-Take MPA, but this is only 0.4 people as well. So while further outreach and/or enforcement might be warranted, the scope of the problem is small relative to overall activities at these sites. This result also highlights that MPA Watch data can be used to help tailor management plans at specific sites: Generally speaking, comparing the different models for different activity categories reveals that some sites have particularly high counts for specific activities, and this

could be useful information for MPA managers or other decision makers – whether or not the site has legal restrictions. For example, results could help with discussion about signage, or the allocation of public education resources. The models enable quick understanding of dynamics related to compliance, potential impacts, and benefits of MPA sites for human visitors, set in a broader context (i.e., overall activity rates).

Fig 5. Counts across all activities for Asilomar, showing the relative proportion of different activities. (Recreational boating and offshore fishing not shown because they are counts of boats rather than people.)

With support from the California Ocean Protection Council the MPA Watch programs are developing a complementary intercept survey protocol to add to the existing activity count protocol. This intercept survey, now entering a second phase of testing, will allow surveyors to approach coastal users and ask them about their reasons for being at the coast, barriers they may experience in accessing these spaces, and their knowledge of MPAs in California and locally. This process allows managers to better understand motivations for a subset of the large number of people who come to California's coast, in order to inform a larger agenda of understanding the human dimensions of Marine Protected Areas.

Conclusion

Our analysis shows the power of both large-scale participatory science data collection and the use of sophisticated statistical models to analyze that data. Further, the analysis is enabled and enhanced by the involvement of the data collectors with the statistical analysis process. Through this iterative, collaborative process, we were able to demonstrate that within protected areas, very few consumptive activities occur but there is still robust non-consumptive human presence, and that non-consumptive activities are the majority of activities along the California coast more generally. These conclusions could not be drawn without the hard work of more than a decade of individuals contributing data, nor without the ability to apply the models to synthesize this rich-but-complex source of information. Our study supports the need for collaborations between modelers and statisticians and large-scale community and citizen science data collection efforts – underlining what is possible when we work together on these ambitious projects.

Supporting information

S1 Appendix. MPA Watch Manual. This appendix is the training manual used by MPA Watch programs throughout the state during the duration of data collection associated with this paper.

S2 Appendix. Additional Methods and Results. This appendix includes detailed descriptions of the predictor variables, alternative modeling approaches considered and tried, and detailed tables and figures of results for all activity categories.

Acknowledgments

We are deeply grateful to the MPA Watch program managers who worked with us to understand the data collection methods, to refine which variables to include in the models, and to interpret the model results, in particular Kirsten Lindquist, Lisa

Gilfillan, Michael Quill, Penny Owens, Ray Hiemstra, Angela Kemsley, Liz Parissenti, Leslie Adler-Ivanbrook, Lillie Mulligan, Erika Delemarre, Rosa Laucci, Ruthie Maloney, Natalie Johnston, Katie O'Donnell, and Emily Parker. The work relies completely on the staff and leadership of many nonprofits and agencies along the California coast, including Tolowa Dee'Ni' Nation, Heal the Bay, Los Angeles Waterkeeper, Environmental Action Committee of West Marin, Eagle Eyes of False Klamath Cove, WILDCOAST, Greater Farallones Association, Pacific Grove Museum of Natural History, Orange County Coastkeeper, Santa Barbara Channelkeeper, California State Parks, and the Otter Project – and on the almost 2,000 surveyors who have contributed to the dataset over the more than ten years of the program. The program benefited from input from the Ocean Science Trust, the California Ocean Protection Council, and the California Department of Fish and Wildlife when the protocol was first developed and systematized across the state. Green Info Network manages the online data archive, including the data entry portal, the web-based data display, and the export process. The project was funded through the Resources Legacy Fund using Once-Through Cooling funds from the state of California. The funder is very engaged in this work at a policy and community level and brought insights into the history of MPA Watch and its role in the broader management context. The funder had an active interest in finding ways to work with and analyze these data at a statewide level in order to advance its utility to management.

References

1. Nayak PK. Making Commons Dynamic: Understanding Change Through Commonisation and Decommonisation. Routledge Studies in Environment, Culture, and Society. Taylor & Francis; 2021. Available from: https://books.google.com/books?id=LFsXEAAAQBAJ.

2. Paniagua P, Rayamajhee V. Governing the global fisheries commons. Marine Policy. 2024;165:106182. doi:10.1016/j.marpol.2024.106182.

3. Taylor P. Planet ocean and marine protected areas: An Opportunity for Ecological Commons Governance. In: Ecological Integrity, Law and Governance. Routledge; 2018. p. 160–169.

4. Lubchenco J, Grorud-Colvert K. OCEAN. Making waves: The science and politics of ocean protection. Science. 2015;350(6259):382–383. doi:10.1126/science.aad5443.

5. Grorud-Colvert K, Sullivan-Stack J, Roberts C, Constant V, Horta E Costa B, Pike EP, et al. The MPA Guide: A framework to achieve global goals for the ocean. Science. 2021;373(6560):eabf0861. doi:10.1126/science.abf0861.

6. Brockington D, Wilkie D. Protected areas and poverty. Philosophical Transactions of the Royal Society B: Biological Sciences. 2015;370(1681):20140271. doi:10.1098/rstb.2014.0271.

7. Biber E. The challenge of collecting and using environmental monitoring data. Ecology and Society. 2013;18(4). doi:10.5751/ES-06117-180468.

8. Gill DA, Mascia MB, Ahmadia GN, Glew L, Lester SE, Barnes M, et al. Capacity shortfalls hinder the performance of marine protected areas globally. Nature. 2017;543(7647):665–669. doi:10.1038/nature21708.

9. Pike EP, MacCarthy JMC, Hameed SO, Harasta N, Grorud-Colvert K, Sullivan-Stack J, et al. Ocean protection quality is lagging behind quantity: Applying a scientific framework to assess real marine protected area progress against the 30 by 30 target. Conservation Letters. 2024;17(3):e13020. doi:10.1111/conl.13020.

10. Mast A, Gill D, Ahmadia GN, Darling ES, Andradi-Brown DA, Geldman J, et al. Shared governance increases marine protected area effectiveness. PLOS ONE. 2025;20(1):1–21. doi:10.1371/journal.pone.0315896.

11. Mutanga CN, Vengesayi S, Muboko N, Gandiwa E. Towards harmonious conservation relationships: A framework for understanding protected area staff-local community relationships in developing countries. Journal for Nature Conservation. 2015;25:8–16. doi:10.1016/j.jnc.2015.02.006.

12. Cigliano JA, Ballard HL, editors. Citizen Science for Coastal and Marine Conservation. 1st ed. Abingdon, Oxon : Routledge, 2017. | Series: Earthscan oceans: Routledge; 2017. Available from: `https://www.taylorfrancis.com/books/9781317272618`.

13. Aceves-Bueno E, Adeleye AS, Bradley D, Tyler Brandt W, Callery P, Feraud M, et al. Citizen Science as an Approach for Overcoming Insufficient Monitoring and Inadequate Stakeholder Buy-in in Adaptive Management: Criteria and Evidence. Ecosystems. 2015;18(3):493–506. doi:10.1007/s10021-015-9842-4.

14. McKinley DC, Miller-Rushing AJ, Ballard HL, Bonney R, Brown H, Evans DM, et al. Investing in citizen science can improve natural resource management and environmental protection. Issues in Ecology. 2015;2015(19):1–27.

15. Vohland K, Land-Zandstra A, Ceccaroni L, Lemmens R, Perelló J, Ponti M, et al., editors. The Science of Citizen Science. Cham: Springer International Publishing; 2021. Available from: `https://link.springer.com/10.1007/978-3-030-58278-4`.

16. Sullivan BL, Aycrigg JL, Barry JH, Bonney RE, Bruns N, Cooper CB, et al. The eBird enterprise: An integrated approach to development and application of citizen science. Biological Conservation. 2014;169:31–40. doi:10.1016/j.biocon.2013.11.003.

17. Johnston A, Matechou E, Dennis EB. Outstanding challenges and future directions for biodiversity monitoring using citizen science data. Methods in Ecology and Evolution. 2022; p. 2041–210X.13834. doi:10.1111/2041-210X.13834.

18. Chapman M, Goldstein BR, Schell CJ, Brashares JS, Carter NH, Ellis-Soto D, et al. Biodiversity monitoring for a just planetary future. Science. 2024;383(6678):34–36. doi:10.1126/science.adh8874.

19. Johnston A, Fink D, Hochachka WM, Kelling S. Estimates of observer expertise improve species distributions from citizen science data. Methods in Ecology and Evolution. 2018;9(1):88–97. doi:10.1111/2041-210X.12838.

20. Stoudt S, Goldstein BR, de Valpine P. Identifying engaging bird species and traits with community science observations. Proceedings of the National Academy of Sciences. 2022;119(16):7. doi:10.1073/pnas.2110156119.

21. Di Cecco GJ, Barve V, Belitz MW, Stucky BJ, Guralnick RP, Hurlbert AH. Observing the Observers: How Participants Contribute Data to iNaturalist and Implications for Biodiversity Science. BioScience. 2021;71(11):1179–1188. doi:10.1093/biosci/biab093.

22. Carlen EJ, Estien CO, Caspi T, Perkins D, Goldstein BR, Kreling SES, et al. A framework for contextualizing social-ecological biases in contributory science data. People and Nature. 2024;6(2):377–390. doi:10.1002/pan3.10592.

23. Freitag A, Meyer R, Whiteman L. Strategies Employed by Citizen Science Programs to Increase the Credibility of Their Data. Citizen Science: Theory and Practice. 2016;1(1). doi:10.5334/cstp.6.

24. Eitzel M, Meyer R, Ulle N, Oldach E, Lubell M, Dobbins M. Using MPA Watch Data to Analyze Human Activities Along the California Coast; 2022. Available from: https://doi.org/10.58076/C6WC7W.

25. Legislature CS. Marine Life Protection Act; 1999. Available from: https://leginfo.legislature.ca.gov/faces/codes_displayText.xhtml?lawCode=FGC&division=3.&title=&part=&chapter=10.5.&article=.

26. Kirlin J, Caldwell M, Gleason M, Weber M, Ugoretz J, Fox E, et al. California's Marine Life Protection Act Initiative: Supporting implementation of legislation establishing a statewide network of marine protected areas. Ocean & Coastal Management. 2013;74:3–13. doi:10.1016/j.ocecoaman.2012.08.015.

27. California Ocean Protection Council. The California Collaborative Approach: Marine Protected Areas Partnership Plan. Sacramento: California Ocean Protection Council; 2014. Available from: https://opc.ca.gov/webmaster/ftp/pdf/docs/mpa/APPROVED_FINAL_MPA_Partnership_Plan_12022014.pdf.

28. California Department of Fish and Wildlife. California's Marine Protected Area Network Decadal Management Review.; 2022. Available from: https://wildlife.ca.gov/Conservation/Marine/MPAs/Management/Decadal-Review%23566381264-2022-review-report.

29. Meyer R, Meyer E, Sievanen L, Freitag A. Using citizen science to inform ocean and coastal resource management. In: Citizen Science for Coastal and Marine Conservation. Routledge; 2017. p. 132–152.

30. Freiwald J, Meyer R, Caselle JE, Blanchette CA, Hovel K, Neilson D, et al. Citizen science monitoring of marine protected areas: Case studies and recommendations for integration into monitoring programs. Marine Ecology. 2018;39:e12470. doi:10.1111/maec.12470.

31. California Ocean Science Trust. Citizen Science & Ocean Resource Management in California: Guidance for forming productive partnerships. Oakland, CA: California Ocean Science Trust; 2014. Available from: https://education.ucdavis.edu/sites/main/files/ost_-_2014_-_citizen_science__ocean_resource_management_in_california_guidance_for_forming_productive_partnerships.pdf.

32. Meyer R, Korabik A, Harwell T, Petersen N, Ballard H. Examining the role of community and citizen science in marine protected area implementation; 2022. Available from: https://escholarship.org/uc/item/4f89v0b5.

33. Francisco S, Chen C, Planning M, Manager P, Steinback C. Monitoring Human Uses in the North Central Coast of California Ecotrust. 2011;.

34. Scholz A. Establishing Consumptive and Non-consumptive Human Use Baseline Indicators for MPAs in the South Coast of California; 2011.

35. Brooks ME, Kristensen K, van Benthem KJ, Magnusson A, Berg CW, Nielsen A, et al. glmmTMB Balances Speed and Flexibility Among Packages for Zero-inflated Generalized Linear Mixed Modeling. The R Journal. 2017;9(2):378–400. doi:10.32614/RJ-2017-066.

36. R Core Team. R: A Language and Environment for Statistical Computing; 2025. Available from: https://www.R-project.org/.

37. Hartig F. DHARMa: Residual Diagnostics for Hierarchical (Multi-Level / Mixed) Regression Models; 2024. Available from: https://CRAN.R-project.org/package=DHARMa.

38. Hothorn T, Bretz F, Westfall P. Simultaneous Inference in General Parametric Models. Biometrical Journal. 2008;50(3):346–363. doi:10.1002/bimj.200810425.

39. Benjamini Y, Hochberg Y. Controlling the False Discovery Rate: A Practical and Powerful Approach to Multiple Testing. Journal of the Royal Statistical Society: Series B (Methodological). 1995;57(1):289–300. doi:10.1111/j.2517-6161.1995.tb02031.x.

40. Shadish WR, Cook TD, Campbell DT. Experimental and Quasi-experimental Designs for Generalized Causal Inference. Houghton Mifflin; 2002.

41. Meyer BD. Natural and Quasi-Experiments in Economics. Journal of Business & Economic Statistics. 1995;13(2):151–161. doi:10.1080/07350015.1995.10524589.

42. Abadie A, Alexis D, Hainmueller J. Synthetic Control Methods for Comparative Case Studies: Estimating the Effect of California's Tobacco Control Program. Journal of the American Statistical Association. 2010;105(490):493–505. doi:10.1198/jasa.2009.ap08746.

43. Arnott JC, Lemos MC. Understanding knowledge use for sustainability. Environmental Science & Policy. 2021;120:222–230. doi:10.1016/j.envsci.2021.02.016.

44. Cash DW, Clark WC, Alcock F, Dickson NM, Eckley N, Guston DH, et al. Knowledge Systems for Sustainable Development. Proceedings of the National Academy of Sciences. 2003;100(14):8086–8091. doi:10.1073/pnas.1231332100.

45. Chambers JM, Wyborn C, Ryan ME, Reid RS, Riechers M, Serban A, et al. Six modes of co-production for sustainability. Nature Sustainability. 2021;4(11):983–996. doi:10.1038/s41893-021-00755-x.

46. Jagannathan K, Emmanuel G, Arnott J, Mach KJ, Bamzai-Dodson A, Goodrich K, et al. A research agenda for the science of actionable knowledge: Drawing from a review of the most misguided to the most enlightened claims in the science-policy interface literature. Environmental Science & Policy. 2023;144:174–186. doi:10.1016/j.envsci.2023.03.004.

47. Free CM, Smith JG, Lopazanski CJ, Brun J, Francis TB, Eurich JG, et al. If you build it, they will come: Coastal amenities facilitate human engagement in marine protected areas. People and Nature. 2023;5(5):1592–1609.

Fig1

Click here to access/download;Figure;Fig1.tif

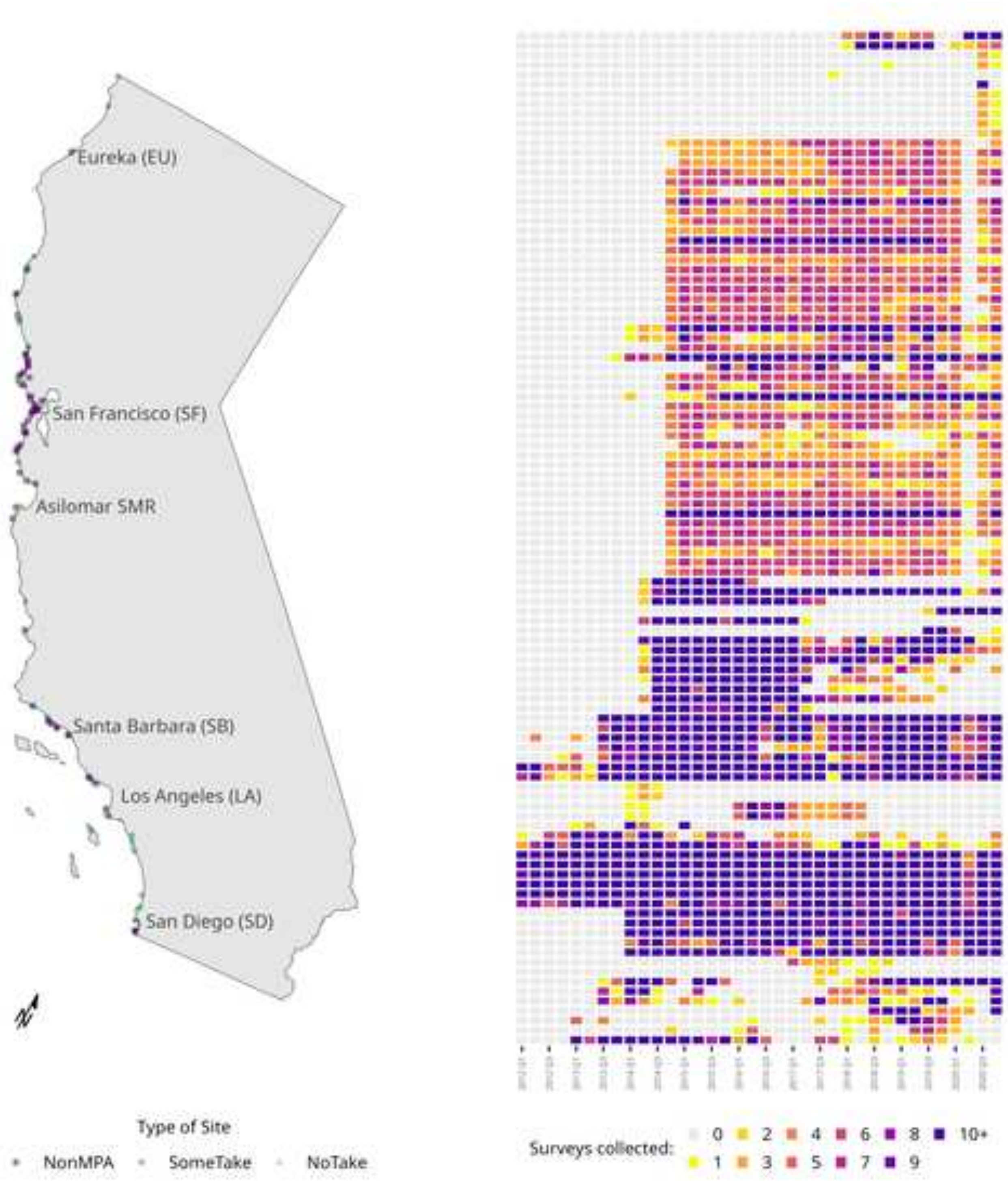

Fig2

Click here to access/download;Figure;Fig2.tif

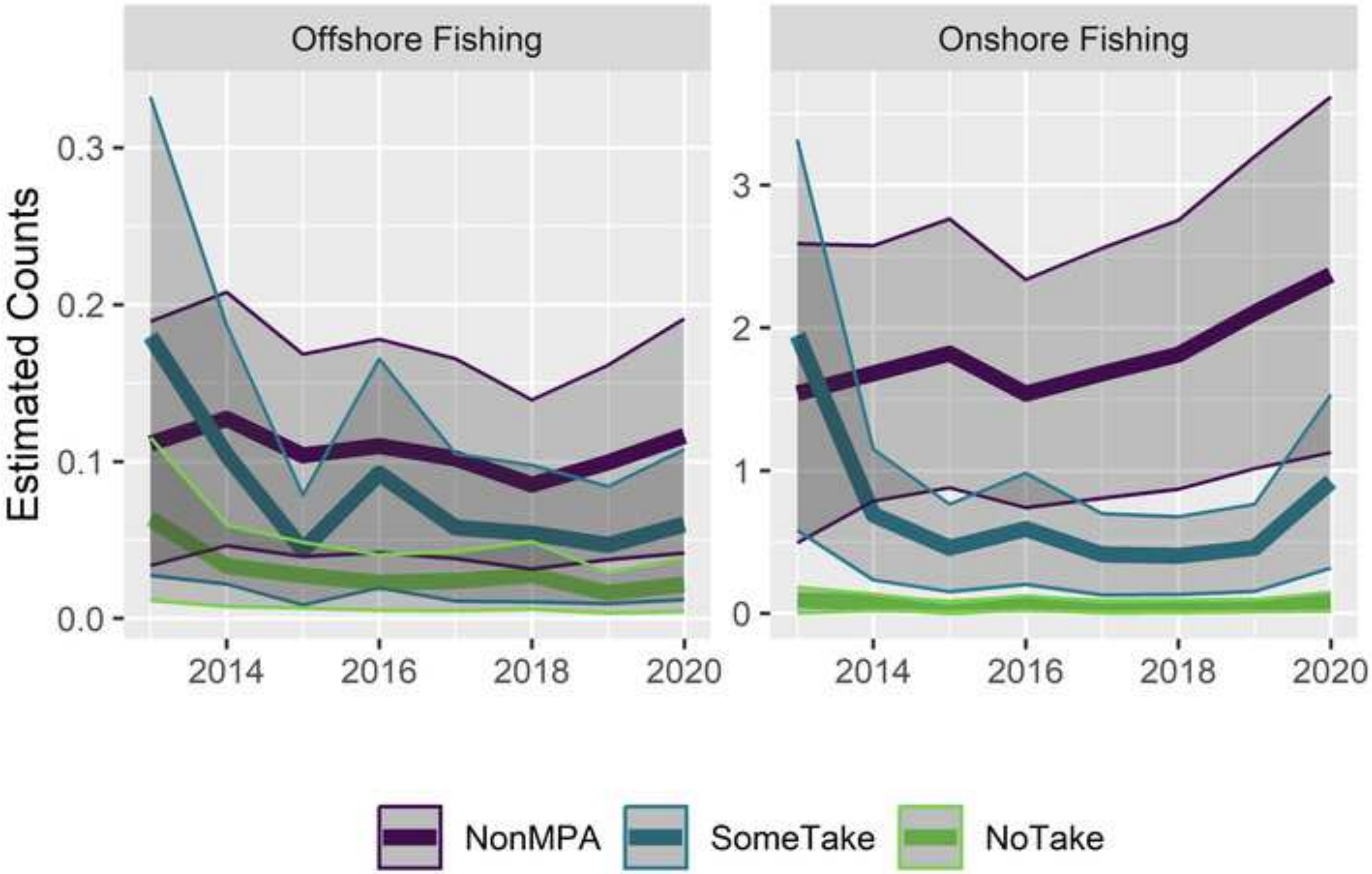

Fig3

Click here to access/download;Figure;Fig3.tif

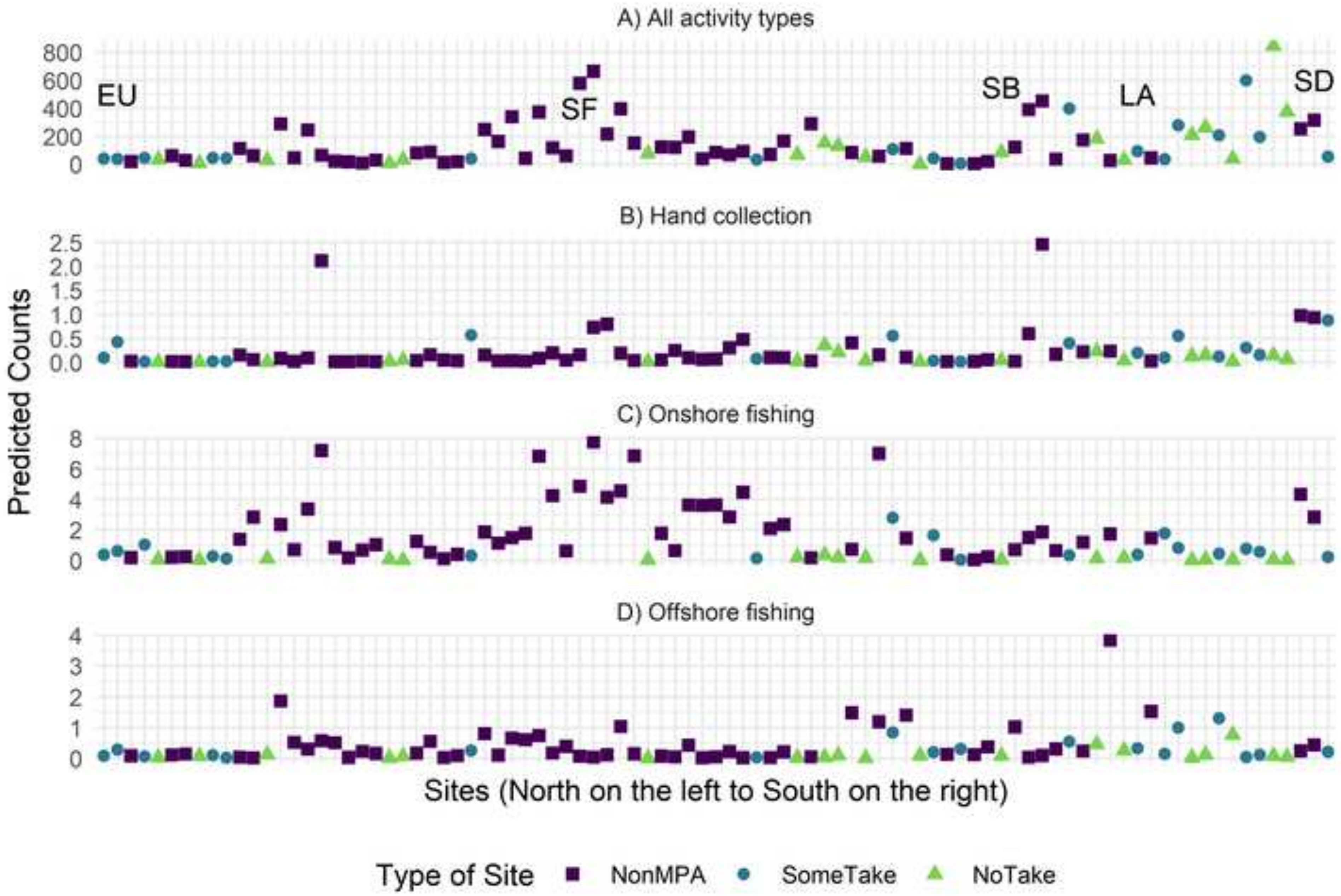

 Click here to access/download;Figure;Fig4.tif

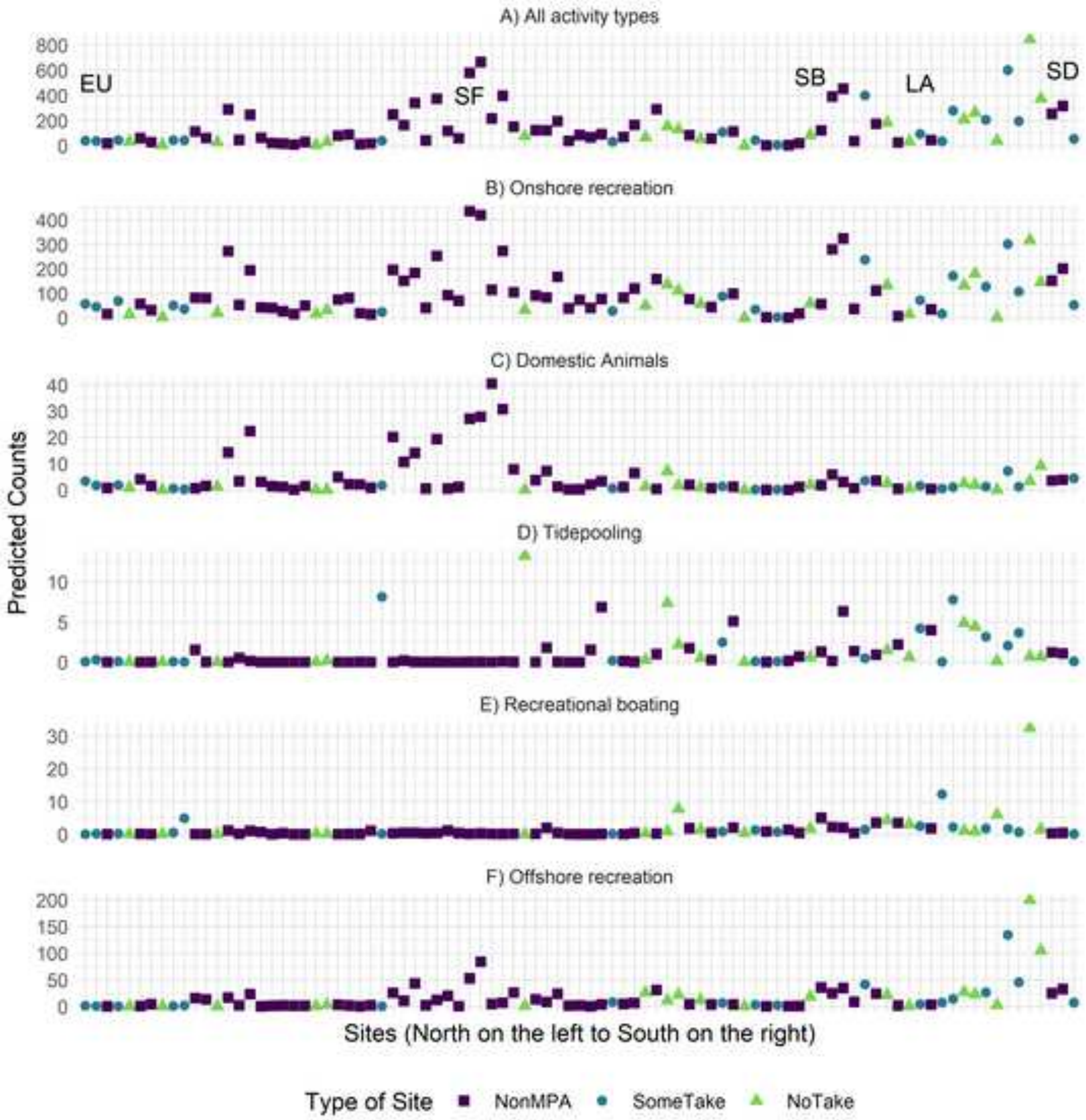

Fig5

Click here to access/download;Figure;Fig5.tif

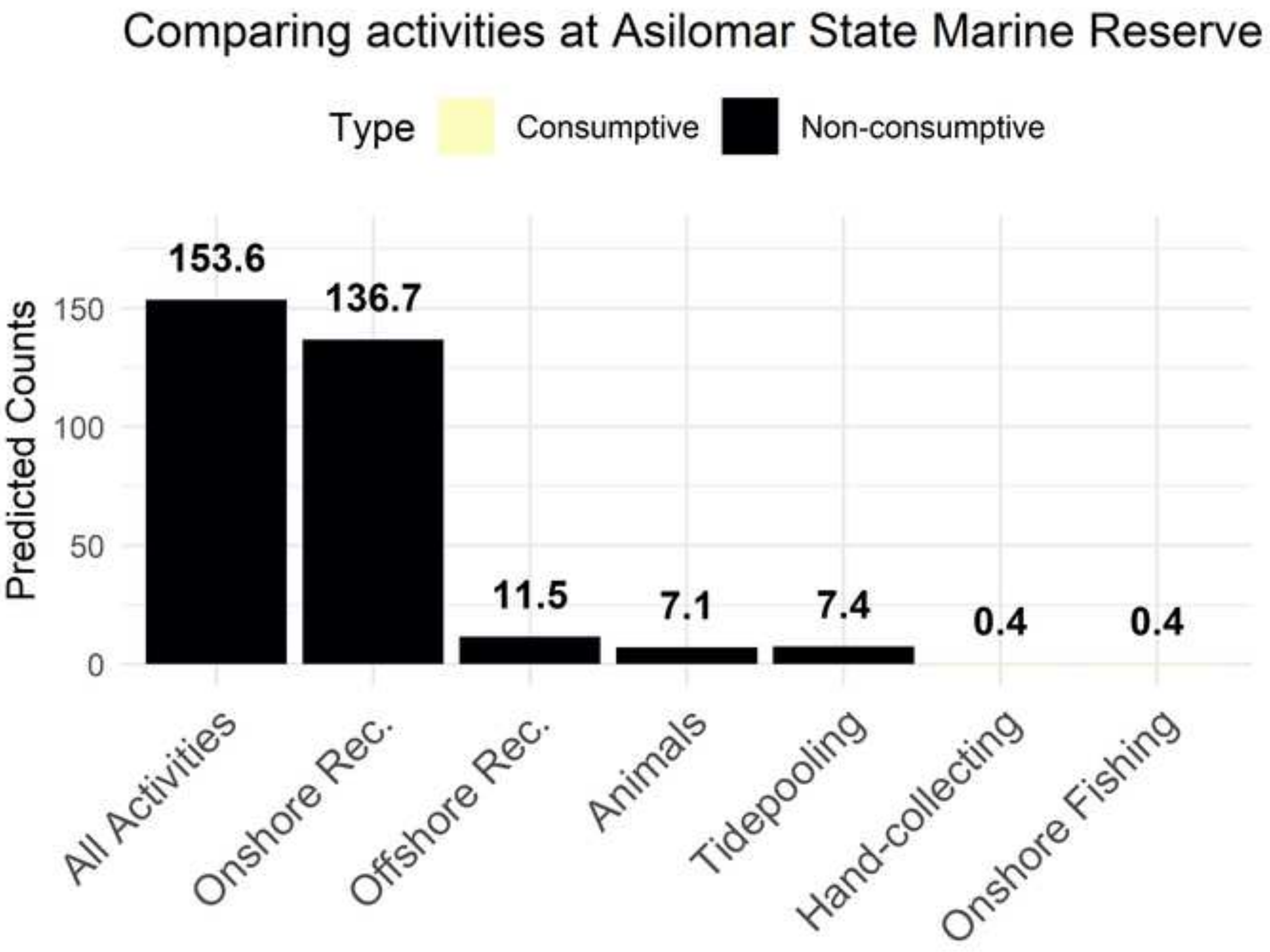

Click here to access/download
Supporting Information
Appendix_S1.pdf

Appendix S2

Click here to access/download
Supporting Information
Appendix_S2.pdf
