# Supplementary material for: Demonstrating conservation impacts in California Marine Protected Areas using large-scale participatory science data
Source: PLoS One. 2026 Jun 25;21(6):e0352449. doi: 10.1371/journal.pone.0352449 (PMC13298793; doi:10.1371/journal.pone.0352449)
Supplement: Attachment — Submitted filename: MPAWatch_PLoS_ResponseToReviewers.pdf [file pone.0352449.s007.pdf]

PONE-D-25-25720 Response to reviewers
Demonstrating conservation impacts in California Marine Protected Areas using sophisticated statistical analysis of large-scale participatory science data

Editor summary:

To strengthen the paper, please address the specific technical omissions and incomplete references identified in the reviewers' notes, while providing more clarity on your methodological choices regarding data omissions and violation proxies.

***

We have elaborated on these points in the new draft and corrected the missing references.

***

We also suggest expanding the discussion to better reflect the existing literature on COVID-19 and providing specific examples of how your collaborative process shaped the research.

***

We have expanded the discussion considerably, including implications for conservation, the potential for COVID-19 impacts, and have added examples of how the collaborative process shaped the research.

***

Finally, streamlining the dense tables and ensuring the language remains objective and consistent throughout will help make your interesting findings even more accessible to our readers.
***

We have moved the detailed tables to the appendix, streamlined the versions in the main manuscript, and have made adjustments to language throughout.

***
Please include the following items when submitting your revised manuscript:

- A letter that responds to each point raised by the academic editor and reviewer(s). You should upload this letter as a separate file labeled 'Response to Reviewers'.
- A marked-up copy of your manuscript that highlights changes made to the original version. You should upload this as a separate file labeled 'Revised Manuscript with Track Changes'.

***
Please note that we have provided the LaTeX-created pdf version of the ‘tracked changes’ as requested, however the tracking changes utility (latexdiff) was unable to show the tables that were changed or removed; therefore to look at table differences, the two versions will have to be looked at side by side.
***

- An unmarked version of your revised paper without tracked changes. You should upload this as a separate file labeled 'Manuscript'.

Journal Requirements:

When submitting your revision, we need you to address these additional requirements.

1. Please ensure that your manuscript meets PLOS ONE's style requirements, including those for file naming. The PLOS ONE style templates can be found at

https://journals.plos.org/ and

https://journals.plos.org/

***

We have downloaded the latest LaTeX template from https://journals.plos.org/plosone/s/latex (Version 3.7 Aug 2025), and reviewed the style requirements at the journal's webpage.

***

2. Thank you for stating the following in the Competing Interests section:

"The project involved close collaborations with funders, decision makers, and community groups that want to improve and publicize the effectiveness of marine conservation. Therefore many conversations focused on results that could be useful in advancing that objective. All authors were compensated from the grants supporting this work, but the funder did not exert undue influence that would downplay or bias against the presentation of neutral or negative results, which are also included in the manuscript, and positive claims about Marine Protected Area effectiveness were highly supported by the data."

Please confirm that this does not alter your adherence to all PLOS ONE policies on sharing data and materials, by including the following statement: "This does not alter our adherence to PLOS ONE policies on sharing data and materials." (as detailed online in our guide for authors http://journals.plos.org/)

Please include your updated Competing Interests statement in your cover letter; we will change the online submission form on your behalf.

***

We have included this statement in our cover letter; please update it in the article's metadata.

***

3. Please note that your Data Availability Statement is currently missing the DOI/accession number of each dataset OR a direct link to access each database. If your manuscript is accepted for publication, you will be asked to provide these details on a very short timeline. We therefore suggest that you provide this information now, though we will not hold up the peer review process if you are unable.

***

We have deposited our underlying data and code at the Environmental Data Initiative, available at: https://doi.org/10.6073/pasta/de206109a75392e0b53cadf9da541483

***

4-7. We note that Figure 1 and S1 Appendix in your submission contain map images which may be

copyrighted. All PLOS content is published under the Creative Commons Attribution License (CC BY 4.0), which means that the manuscript, images, and Supporting Information files will be freely available online, and any third party is permitted to access, download, copy, distribute, and use these materials in any way, even commercially, with proper attribution. For these reasons, we cannot publish previously copyrighted maps or satellite images created using proprietary data, such as Google software (Google Maps, Street View, and Earth). For more information, see our copyright guidelines: http://journals.plos.org/.

We require you to either (1) present written permission from the copyright holder to publish these figures specifically under the CC BY 4.0 license, or (2) remove the figures from your submission:

***

Regarding Figure 1, the state boundary is from the State of California open data repository. We have added a link to this in the figure caption. The city locations are determined from public data.

Regarding Appendix S1 – we will remove the material because it was produced by a different entity than the authors and we will be unable to secure any specific copyrights or photo permissions for the content. We have instead inserted a reference to the material as part of the dataset at the Environmental Data Initiative, anywhere it was referenced before as an appendix. Former Appendix S2 is now the only appendix (S1).

***

8. If the reviewer comments include a recommendation to cite specific previously published works, please review and evaluate these publications to determine whether they are relevant and should be cited. There is no requirement to cite these works unless the editor has indicated otherwise.

***

There were no specific citations suggested by reviewers.

***

5. Review Comments to the Author

Please use the space provided to explain your answers to the questions above. You may also include additional comments for the author, including concerns about dual publication, research ethics, or publication ethics. (Please upload your review as an attachment if it exceeds 20,000 characters)

Reviewer #1: MPA Watch Review

Too much marketing

***

Where appropriate, we have refined the language to be more conservative in stating the usefulness of the data/analysis. That said, because the analysis is in service of management applications, we do not believe that stating the data/analysis' use for these applications constitutes 'marketing.'

***

133-137—needs a clearer description of process, counting vs. interviews

***

The word 'survey' here was meant in the general way, not 'interviews' (which is actually a different social science method than a survey in any case). This paper only analyzes counts of individuals (or animals or boats) engaging in coastal activities, and does not make use of any interview or survey data (in the sense of a coastal participant filling out a form). We have removed the word survey and instead said "some programs also count human activities from a boat"

***

138—missing info

***

This, and the following notes from both reviewers, referred to a bad LaTeX reference to the appendices. We have removed Appendix S1 and have given a reference to the dataset which now includes the manual on the Environmental Data Initiative, and we have corrected the broken references to Appendix S2 (which is now the S1 appendix).

***

141—missing info

***

see above

***

143—145: need clarity on why this was done and how it impacts the sampling

***

We added the following clarification text:

"For example, combining multiple board sports (surfing, boogie-boarding, and 'general board sports') into the larger 'offshore recreation' category avoids inconsistencies between different programs categorizing these activities either more generally or more granularly. The effect of this aggregation is to increase the sample size for a broad category (e.g. 'offshore recreation') and for less-frequented coastal sites this can make it easier to statistically analyze the effect of predictors on activity counts; when there are few occurrences of an activity it can be difficult to estimate the effect of a given predictor."

***

180—missing info
***

see above

***

293—clarify “activities over some time periods.”
***

This section was meant to summarize the detailed results so we only give a brief example to clarify: “(for example, less onshore fishing in 2015-2019 than in 2013-2014 and 2020)”

***

299—there is a very large body of literature focusing on the role of COVID 19 on park and MPA use. This should be discussed in greater detail

***

We have included additional literature on COVID 19 and park/MPA use, but have put it in the discussion section (see the editor’s recommendations). We don’t feel our data support a more concrete or prominent description of the effects of COVID on the patterns we see in the data in the results section.

***

307-313—again, the authors use the term “some” too often, this is quite vague. Please be more specific.
***

We edited this to state the relevant years (e.g. 2016 and 2019, 2015 and 2017-2019) for each statement.

***

351—the point that on-water surveys observed more offshore fishing than shore-based surveys seems to be self-evident. Not sure why this would be even mentioned.
***

This is not necessarily self-evident, any more so than the other findings about e.g. weekends having more activity or summer having more activity. Finding results that feel ‘self-evident’ can help increase confidence in the results. In addition, surveyors typically use binoculars to observe offshore fishing from onshore and therefore can have reasonably similar counts. We have added a phrase reflecting this.

***

384-385—would there be literature to correspond to the authors’ assumptions
***

These statements were intended as hypotheses based on our experience doing the surveys. We have included a statement reflecting this in the section.

***

413—missing info
***

see above

***
438---discussion seems a bit superficial; should be expanded.
***

As per the guidance of Reviewer 2, we have expanded the discussion significantly in all three sections (see below for details).

***

451—Again, these few paragraphs do not adequately provide discussion. What important policy questions? Please list them. Cite applicable literature.
***

These paragraphs are intended as introduction to the larger discussion section and therefore are brief and do not include citations. We have included additional literature in the further sections, and added guidance text indicating that these two paragraphs are introducing the later sections. We have also reworded the example policy question to make it clearer.

***

509—in this section the authors do cite literature, this should be expanded to include other sections of the discussion.
***

We have expanded the other sections of the discussion and included more citations.

***

550--this paper was created from secondary data, so I am unclear how the authors can state that the vast majority of activity is non-consumptive acknowledging the critical role of fishing. Please expand the discussion and methods section to explain to the reader if this secondary data is representative of the overall use.

***

We are unclear on what the reviewer means by 'secondary' data. The MPA Watch data are collected directly by volunteers and uploaded into the online database, from which we drew the data. If the reviewer means that this data is human activities observed by other humans, rather than humans reporting their own activities, fishing records, or assessments of the fishery resources themselves, this is still considered primary data in social science. It is beyond the scope of this paper to directly compare surveyor observations to, for example, records of fishing permits or fisheries data. We have added a section to the discussion called "caveats and limitations" and make this point in that section.

We have also expanded the discussion section about non-consumptive uses, including referring to our earlier report which showed the raw data indicating nonconsumptive activity counts are overwhelmingly higher than consumptive ones, and have given a specific example of a non-protected site to ground this point in another example.

***

580—I feel more comfortable when the authors speak about specific marine reserves, rather than discussing broad use in California.

***

Of course, conclusions based on specific sites can be more easily checked, but one of the strengths of the statewide protocol is that the data can be combined across the state and can support conversations about broad use. This was in fact the desired outcome by the California Department of Fish and Wildlife. Appropriately corrected for and caveated, we believe this is a valid use for the data. That said, we added text to the 'caveats and limitations' section of the discussion to ensure that these caveats and corrections are noted by readers when we are discussing broad use. We have also explored a second specific site in our discussion to help provide additional contrasting sites with similar patterns. In addition, in that section we have deepened our discussion of the broad patterns we see across both the raw data and the modeling predictions.

***

Reviewer #2: Recommended specific revisions by Lines
Title & Abstract
Title: Consider making it more specific. E.g., "...using zero-inflated GLMMs to analyze..." or "...revealing patterns of compliance and recreation."
***

We did consider this edit, but after debating different additional details to include, we felt that 1) it made the title longer and more unwieldy, and 2) it might not reach as broad an audience if it were made more specific. Some of these more specific phrases will appear in keywords and are in the abstract, so hopefully the paper will still be found with respect to these when readers search for those topics.

***

Abstract: Looks excellent. It clearly states the problem, method, key results, and significance.
Introduction
***

Thank you; we appreciate the positive feedback.

***

Lines 13-17: The transition from tragedy of the commons to equity concerns is slightly abrupt. Consider a bridging sentence about how MPAs, as a governance tool, must balance ecological and social objectives.

***

Thank you for this suggestion – we have added a sentence to this effect smoothing the transition from commons to equity.

***

Lines 31-41: The description of CCS is good. Briefly mention the specific analytical challenge this paper addresses (the shift of credibility to the analytical phase, line 46) as a key motivation for your study.

***

We have now mentioned the motivation of the study in the sentence regarding credibility and the analytical phase, and also added a line at the end of the paragraph before 'in this study, we…' in order to emphasize that we want to overcome the complexities of the dataset in order to answer questions about human use of MPAs.

***

Lines 69-76: The five research questions are clear. Consider numbering them (Q1, Q2, etc.) for easy reference later in the manuscript.

***

We had already numbered them in the introduction (but have now added a Q) and have now referenced them by number elsewhere in the manuscript.

***

Materials and Methods

Lines 93-106 (Study System): This section is crucial for contextualizing the data's origin. It's well-described but could be more concise.

***

We've cut this paragraph (on the funding context) down by nearly 50% while preserving the crucial context.

***

Lines 146-156 (Potential Violations): This is a critical methodological choice. Justify more strongly why using observed consumptive activity in no-take zones as a "potential violation" proxy is preferred over the surveyors' assessments. Is it purely for consistency, or also because the surveyor assessment is subjective? This needs clarity.

***

It is for both reasons – consistency, and wider variation in surveyor assessment. We clarified this in the methods section, which now reads: "This is partly due to not all surveyors having filled out this category on the data sheet. In addition, surveyors may also have differed more widely in their assessment of whether an activity was a potential violation, because violations are determined by often-complex rules regarding what is allowed in that location (for example restrictions on specific species or fishing equipment, which may be difficult to see). Assessing a

potential violation directly therefore requires a different level of judgment for surveyors that can vary more widely than for other categories. Therefore we focus on the definition of a potential violation based on activity category and MPA type because we were able to consistently apply it across the dataset. ”

***

Lines 158-162 (Non-MPA sites): Acknowledge this as a study limitation more explicitly. The lack of paired design limits the strength of comparisons. This should be reiterated in the Discussion.

***

We have added language in methods and discussion more explicitly acknowledging this limitation (the discussion now contains a section titled “caveats and limitations”).

***

Lines 184-187 (Data Omission): State how many records were omitted (2,265 for land-based; 2,312 overall) and the percentage (~7.7%). Discuss briefly if this omission could introduce bias (e.g., are surveys with missing weather data systematic in time/space?).

***

Change accepted. In looking closely at the omitted data, we confirmed that these missing values were largely in the weather condition variables (especially ``visibility''), and were mostly in the first two years of the project (2012 and 2013) and at particular sites (e.g. Abalone Cove SMCA). The likely explanation is that when the program was starting up, not everyone was implementing the protocol consistently. Some of these differences will be captured in the program, site, and transect random effects, as well as the separate factors for each year, and these statistical features will control for differences to some degree. This does mean that the interpretation of the meaning of the random effects should include this, as well as reducing the strength of our claims on the results for the first two years.

We have added the following text to the methods section: “These omitted records (2,265 for land-based; 2,312 overall; around 7.7\% of surveys) were largely in the first year (2012) and for particular sites (e.g. Abalone Cove), and the missing variables tended to be the weather variables -- likely due to early efforts to align protocols between sites and programs. This omission is likely to reduce the precision of early-year data and/or estimates for particular sites by reducing the sample size, but other data in those years and for those sites can be used to compensate, and random effects for sites and separate factors for the year will also help control for these differences. ” And we have made notes on these interpretations in the results for years and for the program random effect.

***

Lines 197-285 (Model Implementation): The description is detailed and appropriate for an ecological/modeling journal. For a broader audience, consider moving some of the highly technical details (e.g., specific DHARMa checks, polynomial term selection) to a supplement and keeping the main text focused on the rationale.

***

We have moved all the technical detail regarding statistical model implementation to the Appendix, and simplified (and shortened) the language describing these details, with an emphasis on explaining the concepts and rationale behind the choices, aimed at a more general audience.

***

Line 274-277 (Multiple Testing): Good. Specify that FDR correction was applied within each activity category's set of tests, not across all 624 tests globally.

***

We did state this, but clarified the language to read: "Because this resulted in 624 tests total for all analyses, we applied a multiple testing correction to all p-values WITHIN a given activity category (not across all 624 tests) using the Benjamini-Hochberg ``false discovery rate'' correction."

***

Results
Tables 3-8: These are very dense. Consider simplifying for the main text (e.g., showing only key predictors for key questions) and providing full tables in an appendix. The current presentation is hard to digest.

***

We have moved tables 3-8 as originally written to the Appendix. We have now made modified versions that report in prose the highlights for each activity category in Tables 3, 5, and 7, supporting the summaries given in their corresponding results prose sections. Tables 4, 6, and 8 are adequately described in their respective prose sections of the Results so we have omitted them in the main manuscript since they are now included in the Appendix. (Note that the new tables have been renumbered 3-5)

***

Lines 294-300 (Qualitative Patterns): As noted above, integrate these better. The COVID-19 signal is fascinating and deserves a dedicated sentence in the main results, not just a "pattern for future study."

***

There are a few sentences in the more detailed results section (lines 412-416 in the originally submitted draft) elaborating on this point. We do not feel our study merits more concrete interpretation of these qualitative patterns – a future study could explicitly design the model to answer questions about the pandemic. That said, we have elaborated on the potential for this future study as well as citing the appropriate literature in the discussion section (see above note in response to Reviewer 1 and the editor's suggestion to expand on the potential pandemic effects in the discussion section).

***

Figures 3 & 4: Concept is good. Ensure axis labels are extremely clear. In the caption or on the figure, explicitly state that these are model predictions under standardized conditions, not raw counts. This is vital.

***

The captions explicitly do state that they are model predictions, but we modified the first sentence of the caption to read “Model-predicted counts across all sites in California for all consumptive activities, under standardized favorable conditions (sunny weather at midday, mid-summer, on a weekend day, with average tide level) -- note that these are NOT raw counts from the data.” This will hopefully keep this crucial information prominent to a reader.

***

Discussion

Lines 438-457 (Opening Paragraphs): Strong start, effectively summarizing the process and high-level findings.

***

Thank you for the positive feedback.

***

Lines 458-503 (Lessons Learned): This is an excellent and reflexive section that adds great value. The discussion of occupancy vs. GLMM vs. N-mixture models is particularly useful for other researchers.

***

Glad to hear this feedback; thank you for the perspective.

***

Lines 488-503 (Causal Inference): This paragraph is outstanding. It correctly identifies limitations and points to fruitful future directions. Consider adding a sentence on how even without formal causal inference, the consistent patterns across space (MPA vs. non-MPA) and the declining trends in some consumptive activities provide compelling evidence for a treatment effect.

***

Thank you for this suggestion – we have adopted the Reviewer’s language at the end of the paragraph. It provides a nice connection back to the current results.

***

Lines 504-548 (Collaborative Process): This is a major strength of the manuscript. Be more specific about what was learned from partners that changed the modeling. Give 1-2 concrete examples (e.g., "Partners explained that variable X was recorded inconsistently, leading us to aggregate it...").

***

We have elaborated on the question of the surveyors’ identification of violations and how it led to using the MPA type to identify potential violations. We also added an example regarding the

hand-collection activity and how it came about that we included it, and how including it improved the analysis.

***

Lines 549-603 (Implications for Conservation): Good, but could be tighter.

***

This section has been substantially reorganized and the logical flow should now be improved – first two paragraphs talk about the context for social science research in coastal conservation, the next paragraph discusses nonconsumptive behavior being more than consumptive, and recreation not being deterred. The fourth paragraph focuses on compliance and protection, leading into site-specific advice, and then the fifth paragraph focuses on Asilomar example. Then the final paragraph both develops this idea more fully (re: site specific advice) and then scopes back out to the intercept survey and the broader utility of all these data for both statewide and local planning.

***

Lines 554-560: The point about non-consumptive use being unaffected is critical. Frame it as a key management success: protection achieved without deterring public enjoyment.

***

Change implemented (using the reviewer's language). This is now the last sentence of the paragraph, so should help emphasize the point.

***

Lines 567-579 (Compliance): Excellent. The nuance about "potential" violations and the scale being small is perfectly stated.

***

Thank you for the positive feedback.

***

Lines 580-594 (Asilomar Example): This is very effective. Consider adding a second, contrasting example (e.g., a site with very high recreation but zero predicted violations) to show the utility of the approach across contexts.

***

Thank you for the positive feedback. In the process of drafting the paper, we did create an example of a site that had no predicted violations (because it was a non-MPA site) and had very high predicted total activity counts. We attempted to create a comparative figure but the visual was not very compelling. That said, we have introduced more material on this site (Carpenteria) in the discussion section when talking about the overall pattern of higher non-consumptive uses, and contrasted it with Asilomar. We have also added the figures comparing the two to the appendix.

***

Lines 595-603 (Intercept Survey): This future direction is relevant but feels a bit tacked on. Integrate it better, perhaps as an example of how this analysis has directly spurred the next phase of the program's development.

***

We have edited this discussion section to more clearly indicate how the intercept survey works with the count protocol; the paragraph now has a context that includes both protocols in a more wholistic framing.

***

Minor Corrections & Suggestions
Line 4: "oft-disproven" – consider a more academic phrasing like "widely debated" or "critiqued."

***

We have elected just to omit 'oft-disproven' and leave the sentence at 'so-called tragedy of the commons' which already indicates that it's critiqued.

***

Line 75: Type: "actvity" -> "activity".
Line 122: Type: "actvities" -> "activities".

***

Thank you for catching these. We've corrected them.

***

Line 137: "for the MPA Watch training manual" – this appears to be an incomplete hyperlink or citation placeholder. Fix.

Line 180: "See for additional detail" – another incomplete reference.
Line 304: "See for tables" – incomplete reference.
***

see above re:Reviewer 1; references to Appendix S1 have been corrected.

***

Consistency: Check consistency in terminology: "CCS" vs. "participatory science" vs. "community science." Choose one primary term and use it throughout, defining it early on.

***

Done; we now use 'participatory science' throughout.

***
