# Supplementary material for: Demonstrating conservation impacts in California Marine Protected Areas using large-scale participatory science data
Source: PLoS One. 2026 Jun 25;21(6):e0352449. doi: 10.1371/journal.pone.0352449 (PMC13298793; doi:10.1371/journal.pone.0352449)
Supplement: Attachment — Submitted filename: MPAWatch_PLoS_ResponseToReviewers_SecondRevision.pdf [file pone.0352449.s008.pdf]

Response to editorial and reviewer comments for PONE-D-25-25720R1

Your manuscript has merit but requires specific revisions to meet PLOS ONE's publication criteria. Please submit a revised version that strengthens the results with quantitative data. You must also significantly shorten the discussion, and move descriptive text into table captions.

The quantitative specifics of our results are completely detailed in the appendix and are summarized qualitatively in the main manuscript at the request of Reviewer #2. We agree with the reviewer's point that this makes the very complex model more accessible to a broader audience, and those who are interested in the specifics can drill down into the appendix sections for quantitative summaries and then specific parameter estimates, as desired.

We have shortened the discussion and moved descriptive text into the table captions. Our responses to the reviewers' specific comments are below.

Reviewers' comments:

Reviewer #1: The authors have successfully completed the revisions requested by the editorial team. Please accept.

Thank you for your feedback.

Reviewer #3: OVERALL COMMENTS
The manuscript investigates human activities in California MPAs using large-scale participatory data collection. The manuscript is well-written, the analyses are robust, and the results provide advances on spatial and temporal trends in human use on a network of MPAs. Authors have done a good job dealing with reviewer comments; however, I have some additional comments related mainly to the presentation of results. Please, see more details below.

Thank you for your comments. We have addressed your specific recommendations below.

SPECIFIC COMMENTS

Title: I recommend changing the title to: Demonstrating conservation impacts in California Marine Protected Areas using large-scale participatory science data

We made this change.

L2-14: Add a definition for protected areas and contextualize readers that is part of area-based conservation strategies.
We made this change.

L100-101: The acronym MPA was described and used in the introduction; there is no need to describe it here again.
We made this change.

L294-302: Add quantitative values to inform readers.
This paragraph is meant as an introduction to the entirety of the results section. The statements made here are general statements that summarize groups of the results that are elaborated below in more detail. We have added a sentence to the end of the paragraph that reads "See below for more detailed, activity category specific results on each research question, and refer to the Appendix for specific numerical values." We agree with Reviewer 2's comments that keeping the large number of numerical results in the appendix makes the main manuscript/paper more understandable for a wider audience.

The term "some-take" is unusual. Consider replacing "partially-protected", the usual term for MPAs that allow extractive activities.

We made this change.

L328-329: The description of the table should be in the table caption. Instead of adding the description in the text, add the summarized description of the results presented in the table and cite the table at the end of the sentence.

We kept the paragraph as is, and added the following summary to the caption: "Sandy beaches had more recreation and domestic animals, many activities had higher counts for sites with more amenities/access, tidepooling was higher for known tidepooling sites, higher population density increased counts in recreational boating and domestic animals, and adjacency to a State Park increased onshore fishing but decreased offshore fishing."

In addition, I miss quantitative values to describe such results in the text and table.
The same for the other sections of the results. Authors do not add quantitative values. I strongly recommend adding quantitative values or results of the texts in the main text. Results need to have values to improve the reader's interpretation.

With all due respect for this suggestion, our initial submission included these quantitative results and Reviewer #2 requested that we move these details to the appendix: "Consider simplifying for the main text (e.g., showing only key predictors for key questions) and providing full tables in an appendix. The current presentation is hard to digest." We agreed with Reviewer #2, undertook a major revision and we believe that as a result, the paper is easier to read for a more generalized audience with the numerical results available in the appendix for the subset of the audience that seeks that level of detail. We would prefer to keep it as-is for this reason.

The discussion is too long (20 paragraphs) and includes 1 figure; I recommend that the authors reduce its length. I recommend reducing the second and third subsections.

We have edited the discussion section to reduce repetition and simplify language. (Note that the length of the discussion is in response to the excellent requests from Reviewers #1 and #2 from the prior revision, and we would like to include the material we developed in response to their comments.)
