# Supplementary material for: Demonstrating conservation impacts in California Marine Protected Areas using large-scale participatory science data
Source: PLoS One. 2026 Jun 25;21(6):e0352449. doi: 10.1371/journal.pone.0352449 (PMC13298793; doi:10.1371/journal.pone.0352449)
Supplement: Attachment — Submitted filename: MPAWatch_PLoS_ResponseToReviewers_ThirdRevision.pdf [file pone.0352449.s009.pdf]

Response to editorial and reviewer comments for PONE-D-25-25720R1

Thank you for submitting your manuscript to PLOS ONE. After careful consideration, there is only one minor revision that needs to be made before your work can be accepted. Please review the comments regarding your table captions and ensure that all empirical results and interpretations are moved into the main text rather than left in the titles.

We have done as Reviewer 3 requested.

Reviewers' comments:

Reviewer #3: The authors make a great home work. I have only a minor comment that may improve text organization and the flow of results. Please, see the specific comment below.

Line 336-339: This sentence is just the description of Table 3, not a result. I recommend that authors show the table explanation in the caption. In the results, explain the core results presented in the table and cite the table. I also noted that the authors presented the table results in the table caption (first sentence). Move such a result to the main text or delete it. In Table 4, the authors also move results to the table description. I also recommend moving to the results or deleting. The table caption needs to provide just enough information for the readers' interpretation; results and insights should be described in the main text.

The same for the other Tables. Describe the result and cite the table. Remove the sentences that describe the table but do not present results, such as “Table 4 shows results for temporal variables”, “Table 5 summarizes the results for tide level, cloud cover, precipitation, and visibility.”

We included these summaries in the Table titles in response to your request to put more information into the table captions; we have clearly misunderstood your original comment. We have removed this information from the captions, removed the description of the table from the results, and moved the table reference to the first sentence of each results section.
