# Supplementary material for: Demonstrating conservation impacts in California Marine Protected Areas using large-scale participatory science data
Source: PLoS One. 2026 Jun 25;21(6):e0352449. doi: 10.1371/journal.pone.0352449 (PMC13298793; doi:10.1371/journal.pone.0352449)
Supplement: Attachment — Submitted filename: MPA Watch Review.docx [file pone.0352449.s002.docx]

MPA Watch Review

Too much marketing

133-137—needs a clearer description of process, counting vs. interviews

138—missing info

141—missing info

143—145: need clarity on why this was done and how it impacts the sampling

180—missing info

293—clarify “activities over *some* time periods.”

299—there is a very large body of literature focusing on the role of COVID 19 on park and MPA use. This should be discussed in greater detail

307-313—again, the authors use the term “some” too often, this is quite vague. Please be more specific.

351—the point that on-water surveys observed more offshore fishing than shore-based surveys seems to be self-evident. Not sure why this would be even mentioned.

384-385—would there be literature to correspond to the authors’ assumptions

413—missing info

438---discussion seems a bit superficial; should be expanded.

451—Again, these few paragraphs do not adequately provide discussion. What important policy questions? Please list them. Cite applicable literature.

509—in this section the authors do cite literature, this should be expanded to include other sections of the discussion.

550--this paper was created from secondary data, so I am unclear how the authors can state that the vast majority of activity is non-consumptive acknowledging the critical role of fishing. Please expand the discussion and methods section to explain to the reader if this secondary data is representative of the overall use.

580—I feel more comfortable when the authors speak about specific marine reserves, rather than discussing broad use in California.
