# Supplementary material for: Demonstrating conservation impacts in California Marine Protected Areas using large-scale participatory science data
Source: PLoS One. 2026 Jun 25;21(6):e0352449. doi: 10.1371/journal.pone.0352449 (PMC13298793; doi:10.1371/journal.pone.0352449)
Supplement: Attachment — Submitted filename: Reviewer Attachment from Dr. Abdus Samad.docx [file pone.0352449.s004.docx]

**Reviewer’s comments**

**Recommended specific revisions by Lines**

**Title & Abstract**

**Title:** Consider making it more specific. E.g., "...using zero-inflated GLMMs to analyze..." or "...revealing patterns of compliance and recreation."

**Abstract:**  Looks excellent. It clearly states the problem, method, key results, and significance.

**Introduction**

**Lines 13-17:** The transition from tragedy of the commons to equity concerns is slightly abrupt. Consider a bridging sentence about how MPAs, as a governance tool, must balance ecological and social objectives.

**Lines 31-41:** The description of CCS is good. Briefly mention the specific analytical challenge this paper addresses (the shift of credibility to the analytical phase, line 46) as a key motivation for your study.

**Lines 69-76:** The five research questions are clear. Consider numbering them (Q1, Q2, etc.) for easy reference later in the manuscript.

**Materials and Methods**

**Lines 93-106 (Study System):** This section is crucial for contextualizing the data's origin. It's well-described but could be more concise.

**Lines 146-156 (Potential Violations):** This is a critical methodological choice. Justify more strongly why using observed consumptive activity in no-take zones as a "potential violation" proxy is preferred over the surveyors' assessments. Is it purely for consistency, or also because the surveyor assessment is subjective? This needs clarity.

**Lines 158-162 (Non-MPA sites):** Acknowledge this as a study limitation more explicitly. The lack of paired design limits the strength of comparisons. This should be reiterated in the Discussion.

**Lines 184-187 (Data Omission):** State how many records were omitted (2,265 for land-based; 2,312 overall) and the percentage (~7.7%). Discuss briefly if this omission could introduce bias (e.g., are surveys with missing weather data systematic in time/space?).

**Lines 197-285 (Model Implementation):** The description is detailed and appropriate for an ecological/modeling journal. For a broader audience, consider moving some of the highly technical details (e.g., specific DHARMa checks, polynomial term selection) to a supplement and keeping the main text focused on the rationale.

**Line 274-277 (Multiple Testing):** Good. Specify that FDR correction was applied **within** each activity category's set of tests, not across all 624 tests globally.

**Results**

**Tables 3-8:** These are very dense. Consider simplifying for the main text (e.g., showing only key predictors for key questions) and providing full tables in an appendix. The current presentation is hard to digest.

**Lines 294-300 (Qualitative Patterns):** As noted above, integrate these better. The COVID-19 signal is fascinating and deserves a dedicated sentence in the main results, not just a "pattern for future study."

**Figures 3 & 4:** Concept is good. Ensure axis labels are extremely clear. In the caption or on the figure, explicitly state that these are **model predictions under standardized conditions**, not raw counts. This is vital.

**Discussion**

**Lines 438-457 (Opening Paragraphs):** Strong start, effectively summarizing the process and high-level findings.

**Lines 458-503 (Lessons Learned):** This is an excellent and reflexive section that adds great value. The discussion of occupancy vs. GLMM vs. N-mixture models is particularly useful for other researchers.

**Lines 488-503 (Causal Inference):** This paragraph is outstanding. It correctly identifies limitations and points to fruitful future directions. Consider adding a sentence on how even without formal causal inference, the consistent patterns across space (MPA vs. non-MPA) and the declining trends in some consumptive activities provide compelling evidence for a treatment effect.

**Lines 504-548 (Collaborative Process):** This is a major strength of the manuscript. Be more specific about what was learned from partners that changed the modeling. Give 1-2 concrete examples (e.g., "Partners explained that variable X was recorded inconsistently, leading us to aggregate it...").

**Lines 549-603 (Implications for Conservation):** Good, but could be tighter.

**Lines 554-560:** The point about non-consumptive use being unaffected is critical. Frame it as a key **management success**: protection achieved without deterring public enjoyment.

**Lines 567-579 (Compliance):** Excellent. The nuance about "potential" violations and the scale being small is perfectly stated.

**Lines 580-594 (Asilomar Example):** This is very effective. Consider adding a second, contrasting example (e.g., a site with very high recreation but zero predicted violations) to show the utility of the approach across contexts.

**Lines 595-603 (Intercept Survey):** This future direction is relevant but feels a bit tacked on. Integrate it better, perhaps as an example of how this analysis has directly spurred the next phase of the program's development.

**Minor Corrections & Suggestions**

**Line 4:** "oft-disproven" – consider a more academic phrasing like "widely debated" or "critiqued."

**Line 75:** Type: "actvity" -> "activity".

**Line 122:** Type: "actvities" -> "activities".

**Line 137:** "for the MPA Watch training manual" – this appears to be an incomplete hyperlink or citation placeholder. Fix.

**Line 180:** "See for additional detail" – another incomplete reference.

**Line 304:** "See for tables" – incomplete reference.

**Consistency:** Check consistency in terminology: "CCS" vs. "participatory science" vs. "community science." Choose one primary term and use it throughout, defining it early on.

Final remarks: As a reviewer of this manuscript, I would recommend that the revisions will fundamentally be required for clarity, flow, and emphasis. By improving the narrative, better integrating results, and emphasizing the concrete management implications, this prospective research article has got significant research effects likely to make contribution to conservation science and practice in particular to USA as well as overseas.
