# Supplementary material for: Demonstrating conservation impacts in California Marine Protected Areas using large-scale participatory science data
Source: PLoS One. 2026 Jun 25;21(6):e0352449. doi: 10.1371/journal.pone.0352449 (PMC13298793; doi:10.1371/journal.pone.0352449)
Supplement: Peer review history

# Peer review: Decision Letter 0

**DOI:** 10.1371/journal.pone.0352449.r001

**Authors:** Chih-Cheng Lin

26 Dec 2025

PONE-D-25-25720

Demonstrating conservation impacts in California Marine Protected Areas using sophisticated statistical analysis of large-scale participatory science data

PLOS One

Dear Dr. Eitzel,

Thank you for submitting your manuscript to PLOS ONE. After careful consideration, we feel that it has merit but does not fully meet PLOS ONE’s publication criteria as it currently stands; therefore, we invite you to submit a revised version of the manuscript that addresses the points raised during the review process.

To strengthen the paper, please address the specific technical omissions and incomplete references identified in the reviewers' notes, while providing more clarity on your methodological choices regarding data omissions and violation proxies. We also suggest expanding the discussion to better reflect the existing literature on COVID-19 and providing specific examples of how your collaborative process shaped the research. Finally, streamlining the dense tables and ensuring the language remains objective and consistent throughout will help make your interesting findings even more accessible to our readers.

Please submit your revised manuscript by Feb 09 2026 11:59PM. If you will need more time than this to complete your revisions, please reply to this message or contact the journal office at plosone@plos.org. When you're ready to submit your revision, log on to https://www.editorialmanager.com/pone/ and select the 'Submissions Needing Revision' folder to locate your manuscript file.

Please include the following items when submitting your revised manuscript:

- A letter that responds to each point raised by the academic editor and reviewer(s). You should upload this letter as a separate file labeled 'Response to Reviewers'.
- A marked-up copy of your manuscript that highlights changes made to the original version. You should upload this as a separate file labeled 'Revised Manuscript with Track Changes'.
- An unmarked version of your revised paper without tracked changes. You should upload this as a separate file labeled 'Manuscript'.

If you would like to make changes to your financial disclosure, please include your updated statement in your cover letter. Guidelines for resubmitting your figure files are available below the reviewer comments at the end of this letter.

If applicable, we recommend that you deposit your laboratory protocols in protocols.io to enhance the reproducibility of your results. Protocols.io assigns your protocol its own identifier (DOI) so that it can be cited independently in the future. For instructions see: https://journals.plos.org/plosone/s/submission-guidelines#loc-laboratory-protocols. Additionally, PLOS ONE offers an option for publishing peer-reviewed Lab Protocol articles, which describe protocols hosted on protocols.io. Read more information on sharing protocols at https://plos.org/protocols?utm_medium=editorial-email&utm_source=authorletters&utm_campaign=protocols.

We look forward to receiving your revised manuscript.

Kind regards,

Chih-Cheng Lin, Ph.D.

Academic Editor

PLOS One

**Journal Requirements:**

When submitting your revision, we need you to address these additional requirements.

1. Please ensure that your manuscript meets PLOS ONE's style requirements, including those for file naming. The PLOS ONE style templates can be found at

https://journals.plos.org/plosone/s/file?id=wjVg/PLOSOne_formatting_sample_main_body.pdf and

https://journals.plos.org/plosone/s/file?id=ba62/PLOSOne_formatting_sample_title_authors_affiliations.pdf

2. Thank you for stating the following in the Competing Interests section:

“The project involved close collaborations with funders, decision makers, and community groups that want to improve and publicize the effectiveness of marine conservation. Therefore many conversations focused on results that could be useful in advancing that objective.  All authors were compensated from the grants supporting this work, but the funder did not exert undue influence that would downplay or bias against the presentation of neutral or negative results, which are also included in the manuscript, and positive claims about Marine Protected Area effectiveness were highly supported by the data.”

Please confirm that this does not alter your adherence to all PLOS ONE policies on sharing data and materials, by including the following statement: "This does not alter our adherence to  PLOS ONE policies on sharing data and materials.” (as detailed online in our guide for authors http://journals.plos.org/plosone/s/competing-interests).  If there are restrictions on sharing of data and/or materials, please state these. Please note that we cannot proceed with consideration of your article until this information has been declared.

Please include your updated Competing Interests statement in your cover letter; we will change the online submission form on your behalf.

3. Please note that your Data Availability Statement is currently missing the DOI/accession number of each dataset OR a direct link to access each database. If your manuscript is accepted for publication, you will be asked to provide these details on a very short timeline. We therefore suggest that you provide this information now, though we will not hold up the peer review process if you are unable.

4. We note that Figure 1 and S1 Appendix in your submission contain map images which may be copyrighted. All PLOS content is published under the Creative Commons Attribution License (CC BY 4.0), which means that the manuscript, images, and Supporting Information files will be freely available online, and any third party is permitted to access, download, copy, distribute, and use these materials in any way, even commercially, with proper attribution. For these reasons, we cannot publish previously copyrighted maps or satellite images created using proprietary data, such as Google software (Google Maps, Street View, and Earth). For more information, see our copyright guidelines: http://journals.plos.org/plosone/s/licenses-and-copyright.

We require you to either (1) present written permission from the copyright holder to publish these figures specifically under the CC BY 4.0 license, or (2) remove the figures from your submission:

1. You may seek permission from the original copyright holder of Figure 1 and S1 Appendix to publish the content specifically under the CC BY 4.0 license.

We recommend that you contact the original copyright holder with the Content Permission Form (http://journals.plos.org/plosone/s/file?id=7c09/content-permission-form.pdf) and the following text:

“I request permission for the open-access journal PLOS ONE to publish XXX under the Creative Commons Attribution License (CCAL) CC BY 4.0 (http://creativecommons.org/licenses/by/4.0/). Please be aware that this license allows unrestricted use and distribution, even commercially, by third parties. Please reply and provide explicit written permission to publish XXX under a CC BY license and complete the attached form.”

Please upload the completed Content Permission Form or other proof of granted permissions as an "Other" file with your submission.

In the figure caption of the copyrighted figure, please include the following text: “Reprinted from [ref] under a CC BY license, with permission from [name of publisher], original copyright [original copyright year].”

2. If you are unable to obtain permission from the original copyright holder to publish these figures under the CC BY 4.0 license or if the copyright holder’s requirements are incompatible with the CC BY 4.0 license, please either i) remove the figure or ii) supply a replacement figure that complies with the CC BY 4.0 license. Please check copyright information on all replacement figures and update the figure caption with source information. If applicable, please specify in the figure caption text when a figure is similar but not identical to the original image and is therefore for illustrative purposes only.

The following resources for replacing copyrighted map figures may be helpful:

USGS National Map Viewer (public domain): http://viewer.nationalmap.gov/viewer/

The Gateway to Astronaut Photography of Earth (public domain): http://eol.jsc.nasa.gov/sseop/clickmap/

Maps at the CIA (public domain): https://www.cia.gov/library/publications/the-world-factbook/index.html and https://www.cia.gov/library/publications/cia-maps-publications/index.html

NASA Earth Observatory (public domain): http://earthobservatory.nasa.gov/

Landsat: http://landsat.visibleearth.nasa.gov/

USGS EROS (Earth Resources Observatory and Science (EROS) Center) (public domain): http://eros.usgs.gov/#

Natural Earth (public domain): http://www.naturalearthdata.com/

7. We note that S1 Appendix in your submission contain copyrighted images. All PLOS content is published under the Creative Commons Attribution License (CC BY 4.0), which means that the manuscript, images, and Supporting Information files will be freely available online, and any third party is permitted to access, download, copy, distribute, and use these materials in any way, even commercially, with proper attribution. For more information, see our copyright guidelines: http://journals.plos.org/plosone/s/licenses-and-copyright.

We require you to either (1) present written permission from the copyright holder to publish these figures specifically under the CC BY 4.0 license, or (2) remove the figures from your submission:

1. You may seek permission from the original copyright holder of S1 Appendix to publish the content specifically under the CC BY 4.0 license.

We recommend that you contact the original copyright holder with the Content Permission Form (http://journals.plos.org/plosone/s/file?id=7c09/content-permission-form.pdf) and the following text:

“I request permission for the open-access journal PLOS ONE to publish XXX under the Creative Commons Attribution License (CCAL) CC BY 4.0 (http://creativecommons.org/licenses/by/4.0/). Please be aware that this license allows unrestricted use and distribution, even commercially, by third parties. Please reply and provide explicit written permission to publish XXX under a CC BY license and complete the attached form.”

Please upload the completed Content Permission Form or other proof of granted permissions as an "Other" file with your submission.

In the figure caption of the copyrighted figure, please include the following text: “Reprinted from [ref] under a CC BY license, with permission from [name of publisher], original copyright [original copyright year].”

2. If you are unable to obtain permission from the original copyright holder to publish these figures under the CC BY 4.0 license or if the copyright holder’s requirements are incompatible with the CC BY 4.0 license, please either i) remove the figure or ii) supply a replacement figure that complies with the CC BY 4.0 license. Please check copyright information on all replacement figures and update the figure caption with source information. If applicable, please specify in the figure caption text when a figure is similar but not identical to the original image and is therefore for illustrative purposes only.

8. If the reviewer comments include a recommendation to cite specific previously published works, please review and evaluate these publications to determine whether they are relevant and should be cited. There is no requirement to cite these works unless the editor has indicated otherwise.

[Note: HTML markup is below. Please do not edit.]

**Comments to the Author**

1. Is the manuscript technically sound, and do the data support the conclusions?

The manuscript must describe a technically sound piece of scientific research with data that supports the conclusions. Experiments must have been conducted rigorously, with appropriate controls, replication, and sample sizes. The conclusions must be drawn appropriately based on the data presented.

Reviewer #1: Partly

Reviewer #2: Yes

**********

2. Has the statistical analysis been performed appropriately and rigorously?

Reviewer #1: Yes

Reviewer #2: Yes

**********

3. Have the authors made all data underlying the findings in their manuscript fully available?

The PLOS Data policy requires authors to make all data underlying the findings described in their manuscript fully available without restriction, with rare exception (please refer to the Data Availability Statement in the manuscript PDF file). The data should be provided as part of the manuscript or its supporting information, or deposited to a public repository. For example, in addition to summary statistics, the data points behind means, medians and variance measures should be available. If there are restrictions on publicly sharing data—e.g. participant privacy or use of data from a third party—those must be specified.

Reviewer #1: Yes

Reviewer #2: Yes

**********

4. Is the manuscript presented in an intelligible fashion and written in standard English?

PLOS ONE does not copyedit accepted manuscripts, so the language in submitted articles must be clear, correct, and unambiguous. Any typographical or grammatical errors should be corrected at revision, so please note any specific errors here.

Reviewer #1: Yes

Reviewer #2: Yes

**********

5. Review Comments to the Author

Please use the space provided to explain your answers to the questions above. You may also include additional comments for the author, including concerns about dual publication, research ethics, or publication ethics. (Please upload your review as an attachment if it exceeds 20,000 characters)

Reviewer #1: MPA Watch Review

Too much marketing

133-137—needs a clearer description of process, counting vs. interviews

138—missing info

141—missing info

143—145: need clarity on why this was done and how it impacts the sampling

180—missing info

293—clarify “activities over some time periods.”

299—there is a very large body of literature focusing on the role of COVID 19 on park and MPA use. This should be discussed in greater detail

307-313—again, the authors use the term “some” too often, this is quite vague. Please be more specific.

351—the point that on-water surveys observed more offshore fishing than shore-based surveys seems to be self-evident. Not sure why this would be even mentioned.

384-385—would there be literature to correspond to the authors’ assumptions

413—missing info

438---discussion seems a bit superficial; should be expanded.

451—Again, these few paragraphs do not adequately provide discussion. What important policy questions? Please list them. Cite applicable literature.

509—in this section the authors do cite literature, this should be expanded to include other sections of the discussion.

550--this paper was created from secondary data, so I am unclear how the authors can state that the vast majority of activity is non-consumptive acknowledging the critical role of fishing. Please expand the discussion and methods section to explain to the reader if this secondary data is representative of the overall use.

580—I feel more comfortable when the authors speak about specific marine reserves, rather than discussing broad use in California.

Reviewer #2: Recommended specific revisions by Lines

Title & Abstract

Title: Consider making it more specific. E.g., "...using zero-inflated GLMMs to analyze..." or "...revealing patterns of compliance and recreation."

Abstract: Looks excellent. It clearly states the problem, method, key results, and significance.

Introduction

Lines 13-17: The transition from tragedy of the commons to equity concerns is slightly abrupt. Consider a bridging sentence about how MPAs, as a governance tool, must balance ecological and social objectives.

Lines 31-41: The description of CCS is good. Briefly mention the specific analytical challenge this paper addresses (the shift of credibility to the analytical phase, line 46) as a key motivation for your study.

Lines 69-76: The five research questions are clear. Consider numbering them (Q1, Q2, etc.) for easy reference later in the manuscript.

Materials and Methods

Lines 93-106 (Study System): This section is crucial for contextualizing the data's origin. It's well-described but could be more concise.

Lines 146-156 (Potential Violations): This is a critical methodological choice. Justify more strongly why using observed consumptive activity in no-take zones as a "potential violation" proxy is preferred over the surveyors' assessments. Is it purely for consistency, or also because the surveyor assessment is subjective? This needs clarity.

Lines 158-162 (Non-MPA sites): Acknowledge this as a study limitation more explicitly. The lack of paired design limits the strength of comparisons. This should be reiterated in the Discussion.

Lines 184-187 (Data Omission): State how many records were omitted (2,265 for land-based; 2,312 overall) and the percentage (~7.7%). Discuss briefly if this omission could introduce bias (e.g., are surveys with missing weather data systematic in time/space?).

Lines 197-285 (Model Implementation): The description is detailed and appropriate for an ecological/modeling journal. For a broader audience, consider moving some of the highly technical details (e.g., specific DHARMa checks, polynomial term selection) to a supplement and keeping the main text focused on the rationale.

Line 274-277 (Multiple Testing): Good. Specify that FDR correction was applied within each activity category's set of tests, not across all 624 tests globally.

Results

Tables 3-8: These are very dense. Consider simplifying for the main text (e.g., showing only key predictors for key questions) and providing full tables in an appendix. The current presentation is hard to digest.

Lines 294-300 (Qualitative Patterns): As noted above, integrate these better. The COVID-19 signal is fascinating and deserves a dedicated sentence in the main results, not just a "pattern for future study."

Figures 3 & 4: Concept is good. Ensure axis labels are extremely clear. In the caption or on the figure, explicitly state that these are model predictions under standardized conditions, not raw counts. This is vital.

Discussion

Lines 438-457 (Opening Paragraphs): Strong start, effectively summarizing the process and high-level findings.

Lines 458-503 (Lessons Learned): This is an excellent and reflexive section that adds great value. The discussion of occupancy vs. GLMM vs. N-mixture models is particularly useful for other researchers.

Lines 488-503 (Causal Inference): This paragraph is outstanding. It correctly identifies limitations and points to fruitful future directions. Consider adding a sentence on how even without formal causal inference, the consistent patterns across space (MPA vs. non-MPA) and the declining trends in some consumptive activities provide compelling evidence for a treatment effect.

Lines 504-548 (Collaborative Process): This is a major strength of the manuscript. Be more specific about what was learned from partners that changed the modeling. Give 1-2 concrete examples (e.g., "Partners explained that variable X was recorded inconsistently, leading us to aggregate it...").

Lines 549-603 (Implications for Conservation): Good, but could be tighter.

Lines 554-560: The point about non-consumptive use being unaffected is critical. Frame it as a key management success: protection achieved without deterring public enjoyment.

Lines 567-579 (Compliance): Excellent. The nuance about "potential" violations and the scale being small is perfectly stated.

Lines 580-594 (Asilomar Example): This is very effective. Consider adding a second, contrasting example (e.g., a site with very high recreation but zero predicted violations) to show the utility of the approach across contexts.

Lines 595-603 (Intercept Survey): This future direction is relevant but feels a bit tacked on. Integrate it better, perhaps as an example of how this analysis has directly spurred the next phase of the program's development.

Minor Corrections & Suggestions

Line 4: "oft-disproven" – consider a more academic phrasing like "widely debated" or "critiqued."

Line 75: Type: "actvity" -> "activity".

Line 122: Type: "actvities" -> "activities".

Line 137: "for the MPA Watch training manual" – this appears to be an incomplete hyperlink or citation placeholder. Fix.

Line 180: "See for additional detail" – another incomplete reference.

Line 304: "See for tables" – incomplete reference.

Consistency: Check consistency in terminology: "CCS" vs. "participatory science" vs. "community science." Choose one primary term and use it throughout, defining it early on.

**********

6. PLOS authors have the option to publish the peer review history of their article (what does this mean?). If published, this will include your full peer review and any attached files.

If you choose “no”, your identity will remain anonymous but your review may still be made public.

**Do you want your identity to be public for this peer review?**   For information about this choice, including consent withdrawal, please see our Privacy Policy.

Reviewer #1: No

Reviewer #2: **Yes:**  Dr. Md. Abdus Samad, Associate Professor, Department of Fisheries and Marine Bioscience, Jashore University of Science and Technology (JUST), Bangladesh

**********

[NOTE: If reviewer comments were submitted as an attachment file, they will be attached to this email and accessible via the submission site. Please log into your account, locate the manuscript record, and check for the action link "View Attachments". If this link does not appear, there are no attachment files.]

To ensure your figures meet our technical requirements, please review our figure guidelines: https://journals.plos.org/plosone/s/figures

You may also use PLOS’s free figure tool, NAAS, to help you prepare publication quality figures: https://journals.plos.org/plosone/s/figures#loc-tools-for-figure-preparation.

NAAS will assess whether your figures meet our technical requirements by comparing each figure against our figure specifications.

Attachment Submitted filename: MPA Watch Review.docx

Attachment Submitted filename: PONE-D-25-25720.pdf

Attachment Submitted filename: Reviewer Attachment from Dr. Abdus Samad.docx

Attachment Submitted filename: Reviewer Attachment.docx

# Author comment: Author response to Decision Letter 1

**DOI:** 10.1371/journal.pone.0352449.r002

26 Feb 2026

Please see attached pdf with responses.

Attachment Submitted filename: MPAWatch_PLoS_ResponseToReviewers.pdf

# Peer review: Decision Letter 1

**DOI:** 10.1371/journal.pone.0352449.r003

**Authors:** Chih-Cheng Lin, Chih-Cheng Lin

4 May 2026

PONE-D-25-25720R1

Demonstrating conservation impacts in California Marine Protected Areas using sophisticated statistical analysis of large-scale participatory science data

PLOS One

Dear Dr. Eitzel,

Your manuscript has merit but requires specific revisions to meet PLOS ONE’s publication criteria. Please submit a revised version that strengthens the results with quantitative data. You must also significantly shorten the discussion, and move descriptive text into table captions.

Please submit your revised manuscript by Jun 18 2026 11:59PM. If you will need more time than this to complete your revisions, please reply to this message or contact the journal office at plosone@plos.org. When you're ready to submit your revision, log on to https://www.editorialmanager.com/pone/ and select the 'Submissions Needing Revision' folder to locate your manuscript file.

Please include the following items when submitting your revised manuscript:

- A letter that responds to each point raised by the academic editor and reviewer(s). You should upload this letter as a separate file labeled 'Response to Reviewers'.
- A marked-up copy of your manuscript that highlights changes made to the original version. You should upload this as a separate file labeled 'Revised Manuscript with Track Changes'.
- An unmarked version of your revised paper without tracked changes. You should upload this as a separate file labeled 'Manuscript'.

If you would like to make changes to your financial disclosure, please include your updated statement in your cover letter. Guidelines for resubmitting your figure files are available below the reviewer comments at the end of this letter.

If applicable, we recommend that you deposit your laboratory protocols in protocols.io to enhance the reproducibility of your results. Protocols.io assigns your protocol its own identifier (DOI) so that it can be cited independently in the future. For instructions see: https://journals.plos.org/plosone/s/submission-guidelines#loc-laboratory-protocols. Additionally, PLOS ONE offers an option for publishing peer-reviewed Lab Protocol articles, which describe protocols hosted on protocols.io. Read more information on sharing protocols at https://plos.org/protocols?utm_medium=editorial-email&utm_source=authorletters&utm_campaign=protocols.

As the corresponding author, your ORCID iD is verified in the submission system and will appear in the published article. PLOS supports the use of ORCID, and we encourage all coauthors to register for an ORCID iD and use it as well. Please encourage your coauthors to verify their ORCID iD within the submission system before final acceptance, as unverified ORCID iDs will not appear in the published article. *Only*   the individual author can complete the verification step; PLOS staff *cannot*   verify ORCID iDs on behalf of authors.

We look forward to receiving your revised manuscript.

Kind regards,

Chih-Cheng Lin, Ph.D.

Academic Editor

PLOS One

Journal Requirements:

1. If the reviewer comments include a recommendation to cite specific previously published works, please review and evaluate these publications to determine whether they are relevant and should be cited. There is no requirement to cite these works unless the editor has indicated otherwise.

2. Please review your reference list to ensure that it is complete and correct. If you have cited papers that have been retracted, please include the rationale for doing so in the manuscript text, or remove these references and replace them with relevant current references. Any changes to the reference list should be mentioned in the rebuttal letter that accompanies your revised manuscript. If you need to cite a retracted article, indicate the article’s retracted status in the References list and also include a citation and full reference for the retraction notice.

[Note: HTML markup is below. Please do not edit.]

Reviewers' comments:

Reviewer's Responses to Questions

**Comments to the Author**

1. If the authors have adequately addressed your comments raised in a previous round of review and you feel that this manuscript is now acceptable for publication, you may indicate that here to bypass the “Comments to the Author” section, enter your conflict of interest statement in the “Confidential to Editor” section, and submit your "Accept" recommendation.

Reviewer #1: All comments have been addressed

Reviewer #3: All comments have been addressed

**********

2. Is the manuscript technically sound, and do the data support the conclusions?

The manuscript must describe a technically sound piece of scientific research with data that supports the conclusions. Experiments must have been conducted rigorously, with appropriate controls, replication, and sample sizes. The conclusions must be drawn appropriately based on the data presented.

Reviewer #1: Yes

Reviewer #3: Yes

**********

3. Has the statistical analysis been performed appropriately and rigorously?

Reviewer #1: Yes

Reviewer #3: Yes

**********

4. Have the authors made all data underlying the findings in their manuscript fully available?

The PLOS Data policy requires authors to make all data underlying the findings described in their manuscript fully available without restriction, with rare exception (please refer to the Data Availability Statement in the manuscript PDF file). The data should be provided as part of the manuscript or its supporting information, or deposited to a public repository. For example, in addition to summary statistics, the data points behind means, medians and variance measures should be available. If there are restrictions on publicly sharing data—e.g. participant privacy or use of data from a third party—those must be specified.

Reviewer #1: Yes

Reviewer #3: Yes

**********

5. Is the manuscript presented in an intelligible fashion and written in standard English?

PLOS ONE does not copyedit accepted manuscripts, so the language in submitted articles must be clear, correct, and unambiguous. Any typographical or grammatical errors should be corrected at revision, so please note any specific errors here.

Reviewer #1: Yes

Reviewer #3: Yes

**********

6. Review Comments to the Author

Please use the space provided to explain your answers to the questions above. You may also include additional comments for the author, including concerns about dual publication, research ethics, or publication ethics. (Please upload your review as an attachment if it exceeds 20,000 characters)

Reviewer #1: The authors have successfully completed the revisions requested by the editorial team. Please accept.

Reviewer #3: OVERALL COMMENTS

The manuscript investigates human activities in California MPAs using large-scale participatory data collection. The manuscript is well-written, the analyses are robust, and the results provide advances on spatial and temporal trends in human use on a network of MPAs. Authors have done a good job dealing with reviewer comments; however, I have some additional comments related mainly to the presentation of results. Please, see more details below.

SPECIFIC COMMENTS

Title: I recommend changing the title to: Demonstrating conservation impacts in California Marine Protected Areas using large-scale participatory science data

L2-14: Add a definition for protected areas and contextualize readers that is part of area-based conservation strategies.

L100-101: The acronym MPA was described and used in the introduction; there is no need to describe it here again.

L294-302: Add quantitative values to inform readers.

The term “some-take” is unusual. Consider replacing “partially-protected”, the usual term for MPAs that allow extractive activities.

L328-329: The description of the table should be in the table caption. Instead of adding the description in the text, add the summarized description of the results presented in the table and cite the table at the end of the sentence. In addition, I miss quantitative values to describe such results in the text and table.

The same for the other sections of the results. Authors do not add quantitative values. I strongly recommend adding quantitative values or results of the texts in the main text. Results need to have values to improve the reader's interpretation.

The discussion is too long (20 paragraphs) and includes 1 figure; I recommend that the authors reduce its length. I recommend reducing the second and third subsections.

**********

7. PLOS authors have the option to publish the peer review history of their article (what does this mean?). If published, this will include your full peer review and any attached files.

If you choose “no”, your identity will remain anonymous but your review may still be made public.

**Do you want your identity to be public for this peer review?**   For information about this choice, including consent withdrawal, please see our Privacy Policy.

Reviewer #1: No

Reviewer #3: No

**********

[NOTE: If reviewer comments were submitted as an attachment file, they will be attached to this email and accessible via the submission site. Please log into your account, locate the manuscript record, and check for the action link "View Attachments". If this link does not appear, there are no attachment files.]

To ensure your figures meet our technical requirements, please review our figure guidelines: https://journals.plos.org/plosone/s/figures

You may also use PLOS’s free figure tool, NAAS, to help you prepare publication quality figures: https://journals.plos.org/plosone/s/figures#loc-tools-for-figure-preparation.

NAAS will assess whether your figures meet our technical requirements by comparing each figure against our figure specifications.

# Author comment: Author response to Decision Letter 2

**DOI:** 10.1371/journal.pone.0352449.r004

9 May 2026

We have made the additional revisions requested by Reviewer #3, with the exception of adding the quantitative results back in; we agreed with Reviewer #2 that the draft was clearer for a broader audience with those results included in the appendix instead.

See Attached for detailed review response.

Attachment Submitted filename: MPAWatch_PLoS_ResponseToReviewers_SecondRevision.pdf

# Peer review: Decision Letter 2

**DOI:** 10.1371/journal.pone.0352449.r005

**Authors:** Chih-Cheng Lin, Chih-Cheng Lin, Chih-Cheng Lin

26 May 2026

PONE-D-25-25720R2

Demonstrating conservation impacts in California Marine Protected Areas using large-scale participatory science data

PLOS One

Dear Dr. Eitzel,

Thank you for submitting your manuscript to PLOS ONE. After careful consideration, there is only one minor revision that needs to be made before your work can be accepted. Please review the comments regarding your table captions and ensure that all empirical results and interpretations are moved into the main text rather than left in the titles.

Please submit your revised manuscript by Jul 10 2026 11:59PM. If you will need more time than this to complete your revisions, please reply to this message or contact the journal office at plosone@plos.org. When you're ready to submit your revision, log on to https://www.editorialmanager.com/pone/ and select the 'Submissions Needing Revision' folder to locate your manuscript file.

Please include the following items when submitting your revised manuscript:

- A letter that responds to each point raised by the academic editor and reviewer(s). You should upload this letter as a separate file labeled 'Response to Reviewers'.
- A marked-up copy of your manuscript that highlights changes made to the original version. You should upload this as a separate file labeled 'Revised Manuscript with Track Changes'.
- An unmarked version of your revised paper without tracked changes. You should upload this as a separate file labeled 'Manuscript'

If you would like to make changes to your financial disclosure, please include your updated statement in your cover letter. Guidelines for resubmitting your figure files are available below the reviewer comments at the end of this letter.

If applicable, we recommend that you deposit your laboratory protocols in protocols.io to enhance the reproducibility of your results. Protocols.io assigns your protocol its own identifier (DOI) so that it can be cited independently in the future. For instructions see: https://journals.plos.org/plosone/s/submission-guidelines#loc-laboratory-protocols. Additionally, PLOS ONE offers an option for publishing peer-reviewed Lab Protocol articles, which describe protocols hosted on protocols.io. Read more information on sharing protocols at https://plos.org/protocols?utm_medium=editorial-email&utm_source=authorletters&utm_campaign=protocols.

As the corresponding author, your ORCID iD is verified in the submission system and will appear in the published article. PLOS supports the use of ORCID, and we encourage all coauthors to register for an ORCID iD and use it as well. Please encourage your coauthors to verify their ORCID iD within the submission system before final acceptance, as unverified ORCID iDs will not appear in the published article. *Only*   the individual author can complete the verification step; PLOS staff *cannot*   verify ORCID iDs on behalf of authors.

We look forward to receiving your revised manuscript.

Kind regards,

Chih-Cheng Lin, Ph.D.

Academic Editor

PLOS One

Journal Requirements:

If the reviewer comments include a recommendation to cite specific previously published works, please review and evaluate these publications to determine whether they are relevant and should be cited. There is no requirement to cite these works unless the editor has indicated otherwise.

Please review your reference list to ensure that it is complete and correct. If you have cited papers that have been retracted, please include the rationale for doing so in the manuscript text, or remove these references and replace them with relevant current references. Any changes to the reference list should be mentioned in the rebuttal letter that accompanies your revised manuscript. If you need to cite a retracted article, indicate the article’s retracted status in the References list and also include a citation and full reference for the retraction notice.

[Note: HTML markup is below. Please do not edit.]

Reviewers' comments:

Reviewer's Responses to Questions

**Comments to the Author**

1. If the authors have adequately addressed your comments raised in a previous round of review and you feel that this manuscript is now acceptable for publication, you may indicate that here to bypass the “Comments to the Author” section, enter your conflict of interest statement in the “Confidential to Editor” section, and submit your "Accept" recommendation.

Reviewer #1: All comments have been addressed

Reviewer #3: All comments have been addressed

**********

2. Is the manuscript technically sound, and do the data support the conclusions?

The manuscript must describe a technically sound piece of scientific research with data that supports the conclusions. Experiments must have been conducted rigorously, with appropriate controls, replication, and sample sizes. The conclusions must be drawn appropriately based on the data presented.

Reviewer #1: Yes

Reviewer #3: Yes

**********

3. Has the statistical analysis been performed appropriately and rigorously?

Reviewer #1: Yes

Reviewer #3: Yes

**********

4. Have the authors made all data underlying the findings in their manuscript fully available?

The PLOS Data policy requires authors to make all data underlying the findings described in their manuscript fully available without restriction, with rare exception (please refer to the Data Availability Statement in the manuscript PDF file). The data should be provided as part of the manuscript or its supporting information, or deposited to a public repository. For example, in addition to summary statistics, the data points behind means, medians and variance measures should be available. If there are restrictions on publicly sharing data—e.g. participant privacy or use of data from a third party—those must be specified.

Reviewer #1: Yes

Reviewer #3: Yes

**********

5. Is the manuscript presented in an intelligible fashion and written in standard English?

PLOS ONE does not copyedit accepted manuscripts, so the language in submitted articles must be clear, correct, and unambiguous. Any typographical or grammatical errors should be corrected at revision, so please note any specific errors here.

Reviewer #1: Yes

Reviewer #3: Yes

**********

6. Review Comments to the Author

Please use the space provided to explain your answers to the questions above. You may also include additional comments for the author, including concerns about dual publication, research ethics, or publication ethics. (Please upload your review as an attachment if it exceeds 20,000 characters)

Reviewer #1: The manuscript has been adequately revised and is ready for publication. I have no concerns regarding dual publication, research ethics or publication ethics.

Reviewer #3: The authors make a great home work. I have only a minor comment that may improve text organization and the flow of results. Please, see the specific comment below.

**Line 336-339: This sentence is just the description of Table 3, not a result. I recommend that authors show the table explanation in the caption. In the results, explain the core results presented in the table and cite the table. I also noted that the authors presented the table results in the table caption (first sentence). Move such a result to the main text or delete it. In Table 4, the authors also move results to the table description. I also recommend moving to the results or deleting. The table caption needs to provide just enough information for the readers' interpretation; results and insights should be described in the main text.**

**The same for the other Tables. Describe the result and cite the table. Remove the sentences that describe the table but do not present results, such as “Table 4 shows results for temporal variables”, “Table 5 summarizes the results for tide level, cloud cover, precipitation, and visibility.”**

**********

7. PLOS authors have the option to publish the peer review history of their article (what does this mean?). If published, this will include your full peer review and any attached files.

If you choose “no”, your identity will remain anonymous but your review may still be made public.

**Do you want your identity to be public for this peer review?**   For information about this choice, including consent withdrawal, please see our Privacy Policy.

Reviewer #1: No

Reviewer #3: No

**********

[NOTE: If reviewer comments were submitted as an attachment file, they will be attached to this email and accessible via the submission site. Please log into your account, locate the manuscript record, and check for the action link "View Attachments". If this link does not appear, there are no attachment files.]

To ensure your figures meet our technical requirements, please review our figure guidelines: https://journals.plos.org/plosone/s/figures

You may also use PLOS’s free figure tool, NAAS, to help you prepare publication quality figures: https://journals.plos.org/plosone/s/figures#loc-tools-for-figure-preparation.

NAAS will assess whether your figures meet our technical requirements by comparing each figure against our figure specifications.

# Author comment: Author response to Decision Letter 3

**DOI:** 10.1371/journal.pone.0352449.r006

29 May 2026

See attached. We have completed the requested minor revision.

Attachment Submitted filename: MPAWatch_PLoS_ResponseToReviewers_ThirdRevision.pdf

# Peer review: Decision Letter 3

**DOI:** 10.1371/journal.pone.0352449.r007

**Authors:** Chih-Cheng Lin, Chih-Cheng Lin, Chih-Cheng Lin, Chih-Cheng Lin

10 Jun 2026

Demonstrating conservation impacts in California Marine Protected Areas using large-scale participatory science data

PONE-D-25-25720R3

Dear Dr. Eitzel,

We’re pleased to inform you that your manuscript has been judged scientifically suitable for publication and will be formally accepted for publication once it meets all outstanding technical requirements.

Within one week, you’ll receive an e-mail detailing the required amendments. When these have been addressed, you’ll receive a formal acceptance letter and your manuscript will be scheduled for publication.

An invoice will be generated when your article is formally accepted. Please note, if your institution has a publishing partnership with PLOS and your article meets the relevant criteria, all or part of your publication costs will be covered. Please make sure your user information is up-to-date by logging into Editorial Manager at Editorial Manager® and clicking the ‘Update My Information' link at the top of the page. For questions related to billing, please contact billing support.

If your institution or institutions have a press office, please notify them about your upcoming paper to help maximize its impact. If they’ll be preparing press materials, please inform our press team as soon as possible -- no later than 48 hours after receiving the formal acceptance. Your manuscript will remain under strict press embargo until 2 pm Eastern Time on the date of publication. For more information, please contact onepress@plos.org.

Kind regards,

Chih-Cheng Lin, Ph.D.

Academic Editor

PLOS One

Additional Editor Comments (optional):

Reviewers' comments:

Reviewer's Responses to Questions

**Comments to the Author**

1. If the authors have adequately addressed your comments raised in a previous round of review and you feel that this manuscript is now acceptable for publication, you may indicate that here to bypass the “Comments to the Author” section, enter your conflict of interest statement in the “Confidential to Editor” section, and submit your "Accept" recommendation.

Reviewer #1: All comments have been addressed

Reviewer #3: All comments have been addressed

**********

2. Is the manuscript technically sound, and do the data support the conclusions?

The manuscript must describe a technically sound piece of scientific research with data that supports the conclusions. Experiments must have been conducted rigorously, with appropriate controls, replication, and sample sizes. The conclusions must be drawn appropriately based on the data presented.

Reviewer #1: Yes

Reviewer #3: Yes

**********

3. Has the statistical analysis been performed appropriately and rigorously?

Reviewer #1: Yes

Reviewer #3: Yes

**********

4. Have the authors made all data underlying the findings in their manuscript fully available?

The PLOS Data policy requires authors to make all data underlying the findings described in their manuscript fully available without restriction, with rare exception (please refer to the Data Availability Statement in the manuscript PDF file). The data should be provided as part of the manuscript or its supporting information, or deposited to a public repository. For example, in addition to summary statistics, the data points behind means, medians and variance measures should be available. If there are restrictions on publicly sharing data—e.g. participant privacy or use of data from a third party—those must be specified.

Reviewer #1: Yes

Reviewer #3: Yes

**********

5. Is the manuscript presented in an intelligible fashion and written in standard English?

PLOS ONE does not copyedit accepted manuscripts, so the language in submitted articles must be clear, correct, and unambiguous. Any typographical or grammatical errors should be corrected at revision, so please note any specific errors here.

Reviewer #1: Yes

Reviewer #3: Yes

**********

6. Review Comments to the Author

Please use the space provided to explain your answers to the questions above. You may also include additional comments for the author, including concerns about dual publication, research ethics, or publication ethics. (Please upload your review as an attachment if it exceeds 20,000 characters)

Reviewer #1: The manuscript has been adequately revised, thank you to the authors. The minor changes have been accomplished.

Reviewer #3: Authors addressed my comments properly. I have no more comments and recommend the acceptance of the current version.

Best regards

**********

7. PLOS authors have the option to publish the peer review history of their article (what does this mean?). If published, this will include your full peer review and any attached files.

If you choose “no”, your identity will remain anonymous but your review may still be made public.

**Do you want your identity to be public for this peer review?**   For information about this choice, including consent withdrawal, please see our Privacy Policy.

Reviewer #1: No

Reviewer #3: **Yes:**  Vinicius Giglio

**********

# Editor report: Acceptance letter

**DOI:** 10.1371/journal.pone.0352449.r008

**Authors:** Chih-Cheng Lin, Chih-Cheng Lin, Chih-Cheng Lin, Chih-Cheng Lin

PONE-D-25-25720R3

PLOS One

Dear Dr. Eitzel,

I'm pleased to inform you that your manuscript has been deemed suitable for publication in PLOS One. Congratulations! Your manuscript is now being handed over to our production team.

At this stage, our production department will prepare your paper for publication. This includes ensuring the following:

* All references, tables, and figures are properly cited

* All relevant supporting information is included in the manuscript submission,

* There are no issues that prevent the paper from being properly typeset

You will receive further instructions from the production team, including instructions on how to review your proof when it is ready. Please keep in mind that we are working through a large volume of accepted articles, so please give us a few days to review your paper and let you know the next and final steps.

Lastly, if your institution or institutions have a press office, please let them know about your upcoming paper now to help maximize its impact. If they'll be preparing press materials, please inform our press team within the next 48 hours. Your manuscript will remain under strict press embargo until 2 pm Eastern Time on the date of publication. For more information, please contact onepress@plos.org.

You will receive an invoice from PLOS for your publication fee after your manuscript has reached the completed accept phase. If you receive an email requesting payment before acceptance or for any other service, this may be a phishing scheme. Learn how to identify phishing emails and protect your accounts at https://explore.plos.org/phishing.

If we can help with anything else, please email us at customercare@plos.org.

Thank you for submitting your work to PLOS ONE and supporting open access.

Kind regards,

PLOS ONE Editorial Office Staff

on behalf of

Dr. Chih-Cheng Lin

Academic Editor

PLOS One
